# HIV epidemiology among female sex workers and their clients in the Middle East and North Africa: systematic review, meta-analyses, and meta-regressions

**DOI:** 10.1186/s12916-019-1349-y

**Published:** 2019-06-24

**Authors:** Hiam Chemaitelly, Helen A. Weiss, Clara Calvert, Manale Harfouche, Laith J. Abu-Raddad

**Affiliations:** 1 Infectious Disease Epidemiology Group, Weill Cornell Medicine-Qatar, Cornell University, Qatar Foundation–Education City, P.O. Box 24144, Doha, Qatar; 2 MRC Tropical Epidemiology Group, London School of Hygiene and Tropical Medicine, London, UK; 3 Department of Infectious Disease Epidemiology, Faculty of Epidemiology and Population Health, London School of Hygiene and Tropical Medicine, London, UK; 4 Department of Healthcare Policy & Research, Weill Cornell Medicine, Cornell University, New York, NY USA; 5 College of Health and Life Sciences, Hamad bin Khalifa University, Doha, Qatar

**Keywords:** HIV, Sexually transmitted infections, Sex workers, Sex work, Prevalence, Incidence, Population size, Risk group size, Middle East and North Africa

## Abstract

**Background:**

HIV epidemiology among female sex workers (FSWs) and their clients in the Middle East and North Africa (MENA) region is poorly understood. We addressed this gap through a comprehensive epidemiological assessment.

**Methods:**

A systematic review of population size estimation and HIV prevalence studies was conducted and reported following PRISMA guidelines. Risk of bias (ROB) assessments were conducted for all included studies using various quality domains, as informed by Cochrane Collaboration guidelines. The pooled mean HIV prevalence was estimated using random-effects meta-analyses. Sources of heterogeneity and temporal trends were identified through meta-regressions.

**Results:**

We identified 270 size estimation studies in FSWs and 42 in clients, and 485 HIV prevalence studies in 287,719 FSWs and 69 in 29,531 clients/proxy populations. Most studies had low ROB in multiple quality domains. The median proportion of reproductive-age women reporting current/recent sex work was 0.6% (range = 0.2–2.4%) and of men reporting currently/recently buying sex was 5.7% (range = 0.3–13.8%). HIV prevalence ranged from 0 to 70% in FSWs (median = 0.1%) and 0–34.6% in clients (median = 0.4%). The regional pooled mean HIV prevalence was 1.4% (95% CI = 1.1–1.8%) in FSWs and 0.4% (95% CI = 0.1–0.7%) in clients. Country-specific pooled prevalence was < 1% in most countries, 1–5% in North Africa and Somalia, 17.3% in South Sudan, and 17.9% in Djibouti. Meta-regressions identified strong subregional variations in prevalence. Compared to Eastern MENA, the adjusted odds ratios (AORs) ranged from 0.2 (95% CI = 0.1–0.4) in the Fertile Crescent to 45.4 (95% CI = 24.7–83.7) in the Horn of Africa. There was strong evidence for increasing prevalence post-2003; the odds increased by 15% per year (AOR = 1.15, 95% CI = 1.09–1.21). There was also a large variability in sexual and injecting risk behaviors among FSWs within and across countries. Levels of HIV testing among FSWs were generally low. The median fraction of FSWs that tested for HIV in the past 12 months was 12.1% (range = 0.9–38.0%).

**Conclusions:**

HIV epidemics among FSWs are emerging in MENA, and some have reached stable endemic levels, although still some countries have limited epidemic dynamics. The epidemic has been growing for over a decade, with strong regionalization and heterogeneity. HIV testing levels were far below the service coverage target of “UNAIDS 2016–2021 Strategy.”

**Electronic supplementary material:**

The online version of this article (10.1186/s12916-019-1349-y) contains supplementary material, which is available to authorized users.

## Background

The Middle East and North Africa (MENA) is one of only two regions where HIV incidence and AIDS-related mortality are rising [1]. Between 2000 and 2015, the increase in the number of new infections was estimated at over a third, while that of AIDS-related deaths, at over threefold [1–3]. MENA has been described as “a real hole in terms of HIV/AIDS epidemiological data” [4], with unknown status and scale of epidemics in multiple countries [5–7].

Despite recent progress in HIV research and surveillance in MENA [8], including the conduct of integrated bio-behavioral surveillance surveys (IBBSS) [5, 9], many of these data are, at best, published in country-level reports, or never analyzed. Since 2007, the “MENA HIV/AIDS Epidemiology Synthesis Project” has maintained an active regional HIV database [6]. The first systematic syntheses of HIV data documented concentrated and emerging epidemics among men who have sex with men (MSM) [10] and people who inject drugs (PWID) [11]. The majority of these epidemics emerged within the last two decades [10, 11].

Although the size of commercial heterosexual sex networks is expected to be much larger than the risk networks of MSM and PWID [6, 7], estimates for the population proportion of female sex workers (FSWs), volume of clients they serve, and geographic and temporal trends in infection remain to be established. This evidence gap was highlighted in the latest gap report by the Joint United Nations Programme on HIV/AIDS (UNAIDS) [3], indicating “a lack of data on the burden of HIV among sex workers in the region” and stressing that “the epidemic among them is poorly understood” though “HIV in every country is expected to disproportionately affect sex workers” [3].

This study characterizes HIV epidemiology among FSWs and their clients in MENA by (1) systematically reviewing and synthesizing all available published and unpublished records documenting population size estimates, population proportions, HIV incidence, and HIV prevalence (including in proxy populations of clients such as male sexually transmitted infection (STI) clinic attendees); (2) estimating, for each population, the pooled mean HIV prevalence per country and regionally; (3) identifying the regional-level associations with prevalence, sources of heterogeneity, and temporal trends; and (4) synthesizing the key measures of sexual and injecting risk behaviors.

## Methods

### Search strategy and selection criteria

Evidence for population size estimate, population proportion, HIV incidence, and HIV prevalence in FSWs and clients was systematically reviewed as per Cochrane’s Collaboration guidelines [12]. Findings were reported following the Preferred Reporting Items for Systematic Reviews and Meta-analyses (PRISMA) guidelines [13] (checklist in Additional file 1: Table S1). MENA definition here includes 23 countries extending from Pakistan to Morocco (Additional file 1: Figure S1), based on the convention in HIV research [6, 7, 10, 11] and on World Health Organization (WHO), UNAIDS, and World Bank definitions [6]. MENA was also classified by subregion comprising Eastern MENA (Afghanistan, Iran, Pakistan), the Fertile Crescent (Egypt, Iraq, Jordan, Lebanon, Palestine, Syria), the Gulf (Bahrain, Kuwait, Oman, Qatar, Saudi Arabia, United Arab Emirates, Yemen), the Horn of Africa (Djibouti, Somalia, recently independent South Sudan), and North Africa (Algeria, Libya, Morocco, Sudan, Tunisia).

Systematic searches were performed, up to July 29, 2018, on ten international-, regional-, and country-level databases; abstract archives of International AIDS Society conferences [14]; and Synthesis Project database which includes country-level and international organizations’ reports and routine data reporting [6, 7] (Additional file 1: Box S1). No language or year restrictions were used.

Titles and abstracts of unique citations were screened for relevance, and full texts of relevant/potentially relevant citations were retrieved for further screening. Any document/report including outcomes of interest based on primary data was eligible for inclusion. Case reports, case series, editorials, commentaries, and studies in populations (such as “vulnerable women”) where overlap with FSWs is implied but engagement in sex work is not explicitly indicated were excluded. Reference lists of reviews and all relevant documents were hand searched for eligible reports.

In this article, the term *study* refers to a specific outcome measure (population size estimate, incidence, or prevalence) in a specific population. Therefore, one report could contribute multiple studies, and one study could be published in different reports. Duplicate study results were included only once using the more detailed report.

### Data extraction and synthesis

Data extraction was performed by HC and double extraction by MH, with discrepancies settled by consensus or by contacting authors. Data were extracted from full texts by native speakers (extraction list in Additional file 1: Box S2).

Population size estimates and population proportions were grouped based on being of national coverage or for specific subnational settings, and distinguishing between current FSWs/clients and history of sex work/ex-client. For FSWs, population proportion is defined as the proportion of all reproductive-age women that are engaged in sex work, that is the exchange of sex for money (sex work as a profession) [15, 16], and for clients, as the proportion of men buying sex from FSWs using money. Studies with mixed or non-representative samples (samples biased towards oversampling FSWs with no estimate adjustment) were excluded.

Due to the paucity of studies directly looking at HIV prevalence in clients of FSW, HIV prevalence studies in male STI clinic attendees, or mixed-sex samples of predominantly men (> 60%), were used as a proxy for HIV prevalence in clients of FSWs [17, 18].

Based on meta-analysis results for the pooled HIV prevalence in FSWs, epidemics were classified as *concentrated* (prevalence > 5%), *intermediate-intensity* (prevalence between 1 and 5%), and *low-level* (prevalence < 1%), as informed by epidemiological relevance and existing conventions [19–21].

HIV incidence studies were identified and reported. Additional contextual information was extracted from FSW studies included in the review. These include age, age at sexual debut, age at sex work initiation, sex work duration, marital status, and HIV/AIDS knowledge and perception of risk, as well as behavioral measures of condom use, injecting drug use, sexual partnerships, and HIV testing.

Data were summarized using medians and ranges.

### Quality assessment

Risk of bias (ROB) assessments for population size estimates/population proportions and for HIV prevalence were conducted as informed by Cochrane Collaboration guidelines [12] (criteria in Additional file 1: Table S2). Briefly, size estimation studies were classified as having “low” versus “high” ROB on each of the three domains assessing the (1) validity of sex work definition/engagement in paid sex (clear/valid definition; otherwise), (2) rigor of estimation methodology (likely-to-yield representative estimate; otherwise), and (3) response rate (≥ 60%; < 60%).

Prevalence studies were similarly classified on each of the four domains assessing the (1) validity of sex work definition/engagement in paid sex (clear/valid definition; otherwise), (2) rigor of sampling methodology (probability-based; non-probability-based), (3) response rate (≥ 60% or ≥ 60% of target sample size reached for studies using respondent-driven or time-location sampling; < 60%), and (4) type of HIV ascertainment (biological assays; self-report).

Studies with missing information for a specific domain were classified as having “unclear” ROB for that domain. Measures only extracted from routine databases were considered of unknown quality, as original reports were not available for assessing ROB, and were not included in the quality assessment. The impact of quality domains on observed prevalence was examined in meta-regression (described below).

### Meta-analyses

Pooled mean HIV prevalence in FSWs and client populations were estimated using random-effects meta-analyses, by country and for the whole region. Variances were stabilized using Freeman-Tukey-type arcsine square-root transformation [22, 23]. Weighting was performed using the inverse-variance method [23, 24]. Pooling was performed using Dersimonian-Laird random-effects models to allow for sampling variation and true heterogeneity [25, 26]. Overall prevalence measures were replaced by their stratified measures where applicable.

Heterogeneity was assessed using Cochran’s *Q* statistic to confirm the existence of heterogeneity, *I*^2^ to estimate the magnitude of between-study variation, and prediction intervals to estimate the 95% interval of distribution of true effect sizes [26, 27].

Meta-analyses were implemented in R version 3.4.2 [28].

### Meta-regression analyses

Random-effects meta-regression analyses were conducted to identify the regional-level associations with HIV prevalence in FSWs, sources of between-study heterogeneity, and temporal trend. Independent variables considered a priori were country/subregion, FSW population type, sample size, median year of data collection, sampling methodology, response rate, validity of sex work definition, and HIV ascertainment (details in Additional file 1: Table S3). The same factors (as applicable) were considered for clients’ meta-regression analyses.

To avoid the exclusion of studies with zero prevalence, an increment of 0.1 was added to the number of events in all studies to calculate the log-transformed odds, that is prevalence/(1 − prevalence), and corresponding variance [29]. Factors showing strong evidence for an association with the odds (*p* value ≤ 0.10) in univariable analysis were included in the multivariable analysis.

Meta-regressions were implemented in Stata/SE v.15.1 [30].

## Results

### Search results and scope of evidence

**Fig. 1:**
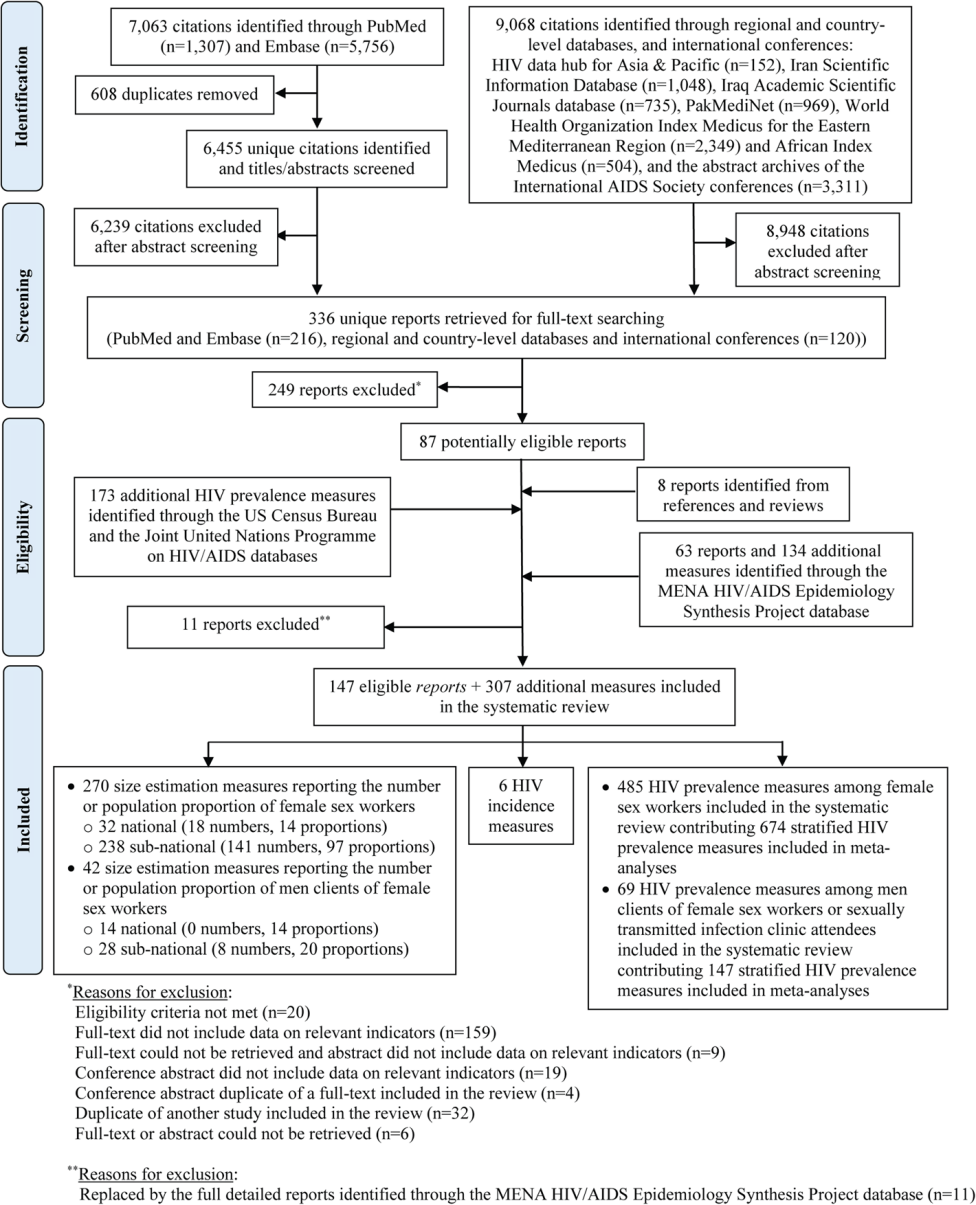
Flow chart of the study selection process in the systematic review following PRISMA guidelines [13]

Figure 1 shows the study selection process. A total of 16,131 citations were identified through databases. After excluding duplicates and title and abstract screening, full texts of 336 unique citations were screened, and 87 reports were eligible for inclusion. Hand-searching of reference lists of relevant reports yielded eight additional eligible reports. Searching US Census Bureau and UNAIDS databases yielded 173 additional measures. Sixty-three detailed country-level reports, 11 of which replaced eligible articles, and 134 additional measures were further identified through Synthesis Project database. In sum, data from 147 eligible reports and 307 additional measures were included. These yielded in total 312 size estimation, 6 HIV incidence, and 554 HIV prevalence measures in FSWs and clients.

Evidence for population size and/or population proportion of FSWs was available for 12 out of 23 MENA countries (270 studies). Population size/population proportion of clients was available in 42 studies from 10 countries. All 6 HIV incidence studies were among FSWs. A total of 485 HIV prevalence studies were identified in 287,719 FSWs from 17 countries and 69 HIV prevalence studies in 29,531 clients (or proxy populations) from 10 countries. Prevalence measures in FSWs and clients contributed respectively 674 and 147 stratified measures for the meta-analyses (overall prevalence measures were replaced by their strata in meta-analyses). For all types of measures, there was a high heterogeneity in data availability across countries.

### Population size estimates and population proportions of FSWs and clients

Table 1 and Additional file 1: Table S4 show the population size estimate and population proportion studies for FSWs and clients at the national and subnational levels, respectively. At the national level, the median number of current/recent FSWs (engaged in sex work in the past year) was 58,934 (range = 2218 in Djibouti to 167,501 in Pakistan), and the median population proportion (out of reproductive-age women aged 15–49 years) was 0.6% (range across studies = 0.2% in Egypt to 2.4% in Iran). The median population proportion of current/recent clients (buying sex from FSWs in the past year) based on diverse samples of general population men was 5.7% (range across studies = 0.3% in Sudan to 13.8% in Lebanon).

**Table 1:** Estimates of some national representation for the number and population proportion of FSWs, and the number and population proportion of clients of FSWs, in the Middle East and North Africa (MENA) reported by identified studies

	Country	Author, year [citation]	Year(s) of data collection	Estimation methodology	Sample type	Reported size estimate				
						Time frame	*N*	Range	%*	Range*
FSWs	Egypt	Bahaa, 2010 [31]	2004–2008	Convenience sample (self-report)	Women seeking VCT testing	NR	NR	NR	0.4	NR
		Jacobsen, 2014 [32]	2014	Enumeration (time-location geographical mapping)	FSWs in urban locations	Current	22,986	6460–26,792	0.24	NR
	Djibouti	WHO, 2011 [33]	2009	NR	FSWs	NR	1000	NR	NR	NR
		WHO, 2011 [33]	2011	Capture-recapture	FSWs	Current	2218	NR	NR	NR
	Iran	WHO, 2011 [33]	2010	Network scale-up	General pop	Current	80,000	NR	NR	NR
		Sharifi, 2017 [34]	2015	Multiplier unique object	FSWs	Current	19,800	10,900–38,100	0.31	0.17–0.58
		Sharifi, 2017 [34]	2015	Network scale-up	General pop	Current	98,500	87,000–109,400	1.54	1.36–1.71
		Sharifi, 2017 [34]	2015	Wisdom of the crowds	FSWs	Current	152,200	93,400–21,4300	2.38	1.46–3.35
	Lebanon	Kahhaleh, 2009 [35]	1996	Pop-based survey (self-report)	General pop (15–49 years)	Past 12 M	NR	NR	0.54	NR
		Kahhaleh, 2009 [35]	2004	Pop-based survey (self-report)	General pop (15–49 years)	Past 12 M	NR	NR	0.53	NR
	Morocco	WHO, 2011 [33]	2010	NR	FSWs	Current	67,000	NR	NR	NR
		Bennani, 2013 [36]	2011	Multiplier unique object	FSWs	Past 6 M	85,000	NR	NR	NR
		MOH, 2013 [37]	2013	Pop-based survey (self-report)	Young women (15–24 years)	Lifetime	NR	NR	6.9	NR
		MOH, 2013 [37]	2013	Pop-based survey (self-report)	Young women (15–24 years)	Current	NR	NR	2.4	NR
	Pakistan	NACP, 2005 [38] (round I)	2005	Enumeration (time-location geographical mapping)	Brothel, kothikhana, home, and street-based FSWs	Current	35,050	30,300–39,800	0.78	NR
		Emmanuel, 2010 [39] (round II)	2006	Enumeration (time-location geographical mapping)	Brothel, kothikhana, home, and street-based FSWs	Current	167,501	NR	0.44	NR
		Emmanuel, 2013 [40, 41] (round IV)	2011–2012	Enumeration (time-location geographical mapping)	Brothel, kothikhana, home, and street-based FSWs	Current	89,178	78,778–99,592	0.72	NR
		NACP, 2017 [42] (round V)	2016–2017	Enumeration (time-location geographical mapping)	Brothel, kothikhana, home, and street-based FSWs	Current	64,829	57,734–70,428	NR	NR
	Sudan	AFROCENTER Group, 2005 [43]	2005	Self-report (convenience sample)	Young women	NR	NR	NR	0.4	NR
	Syria	WHO, 2011 [33]	2011	NR	FSWs	Current	50,000	NR	NR	NR
	Tunisia	WHO, 2011 [33]	2005	NR	FSWs	Current	NR	1000–5000	NR	NR
		WHO, 2011 [33]	2009	NR	FSWs	Current	10,000	NR	NR	NR
		WHO, 2011 [33]	2011	NR	FSWs	Current	25,500	NR	NR	NR
	Yemen	MOH, 2010 [44]	NR	Enumeration (time-location geographical mapping)	FSWs	Current	58,934	NR	NR	1.16–2.10
Clients of FSWs	Afghanistan	Todd, 2007 [45]	2005–2006	Pop-based survey (self-report)	TB patients receiving treatment	Lifetime	NR	NR	3.57	NR
		Todd, 2012 [46]	2010–2011	Pop-based survey (self-report)	Army recruits	Lifetime	NR	NR	12.5	NR
	Egypt	Bahaa, 2010 [31]	2004–2008	Convenience sample (self-report)	Men seeking VCT testing	NR	NR	NR	0.9	NR
	Lebanon	Kahhaleh, 2009 [35]	1996	Pop-based survey (self-report)	General pop (15–49 years)	Past 12 M	NR	NR	9.7	NR
		Adib, 2002 [47]	1999	Pop-based survey (self-report)	Military conscripts	Past 12 M	NR	NR	13.84	NR
		Kahhaleh, 2009 [35]	2004	Pop-based survey (self-report)	General pop (15–49 years)	Past 12 M	NR	NR	5.65	NR
	Morocco	MOH, 2007 [48]	2007	Pop-based survey (self-report)	Young men (15–24 years)	Lifetime	NR	NR	35.3	NR
		MOH, 2007 [48]	2007	Pop-based survey (self-report)	Young men (15–24 years)	Current	NR	NR	2	NR
		MOH, 2013 [37]	2013	Pop-based survey (self-report)	Young men (15–24 years)	Lifetime	NR	NR	10.5	NR
		MOH, 2013 [37]	2013	Pop-based survey (self-report)	Young men (15–24 years)	Current	NR	NR	0.3	NR
	Pakistan	Mir, 2013 [49]	2007	Pop-based survey (self-report)	Urban men (16–45 years)	Lifetime	NR	NR	11.9	NR
		Mir, 2013 [49]	2007	Pop-based survey (self-report)	Urban men (16–45 years)	Past 12 M	NR	NR	5.8	NR
	Sudan	NACP, 2004 [50]	2004	Convenience sample (self-report)	Military personnel	NR	NR	NR	0.3	NR
		AFROCENTER Group, 2005 [43]	2005	Convenience sample (self-report)	Young men	NR	NR	NR	0.5	NR

The table is sorted by year(s) of data collection

*Abbreviations*: *FSWs* female sex workers, *M* months, *MOH* Ministry of Health, *NACP* National AIDS Control Programme, *NR* not reported, *Pop* population, *TB* tuberculosis, *VCT* voluntary counseling and testing, *WHO* World Health Organization

*The decimal places of the population proportion figures are as reported in the original reports

With high heterogeneity in estimation methodology, time frame, and scope between and within countries, it was deemed not meaningful to generate country-specific or regional-pooled estimates for the size/population proportions.

### HIV incidence overview

There were six incidence studies among FSWs (three from each of Somalia and Djibouti; data not shown). Three studies reported zero seroconversions [51, 52]. One study from Somalia reported a cumulative incidence of 2.6% after 6 months of follow-up [51]. The other two from Djibouti—among predominantly Ethiopian FSWs (91%)—reported a cumulative incidence of 3.4% [51] and 11.6% [51] after 3 and 9 months of follow-up, respectively. All incidence studies were conducted before the year 2000 and were limited in scale and scope.

### HIV prevalence overview

HIV prevalence in FSWs ranged from 0 to 70%, with a median of 0.1% (Tables 2 and 3 and Additional file 1: Table S5). There was a high heterogeneity, with almost half of the studies (46.8%) reporting zero prevalence. The median prevalence was 0% (range = 0–14%), 2.0% (range = 0–47.1%), and 18.8% (range = 0–70%) in countries with low-level (prevalence < 1%), intermediate-intensity (prevalence 1–5%), and concentrated epidemics (prevalence > 5%), respectively (epidemic classification based on the results of meta-analyses; see below and Table 5). Ranges indicated pockets of higher HIV prevalence, even in countries with low-level and intermediate-intensity epidemics.

**Table 2:** HIV prevalence in FSWs in the Middle East and North Africa (MENA), as reported in studies using probability-based sampling

Country	Author, year [citation]	Year(s) of data collection	City/province	Study site	Sampling	Population	Sample size	HIV prevalence*	
								%	95% CI
Afghanistan	SAR AIDS HDS, 2008 [53]	2006–2007	Jalalabad	Community	TLS	FSWs	45	0	NR
	SAR AIDS HDS, 2008 [53]	2006–2007	Mazar-i-Sharif	Community	TLS	FSWs	87	0	NR
	NACP, 2010 [54] (round I)	2009	Kabul	Community	RDS	FSWs	368	0	NR
	NACP, 2012 [55] (round II)	2012	Herat	Community	RDS	FSWs	344	0.9	NR
	NACP, 2012 [55] (round II)	2012	Kabul	Community	RDS	FSWs	333	0	NR
	NACP, 2012 [55] (round II)	2012	Mazar-i-Sharif	Community	RDS	FSWs	355	0	NR
Egypt	MOH, 2006 [56] (round I)	2006	Cairo	Community	Conv**	FSWs	118	0.8	NR
	MOH, 2010 [57] (round II)	2010	Cairo	Community	Conv**	FSWs	200	0	NR
Iran	Navadeh, 2012 [58]	2010	Kerman	Community	RDS	FSWs	139	0	NR
	Sajadi, 2013 [59] (round I)	2010	National	Facilities serving vulnerable women	MCS	FSWs	817	4.5	NR
	Kazerooni, 2014 [60]	2010–2011	Shiraz	Community	RDS	FSWs	278	4.7	NR
	Moaeyedi-Nia, 2016 [61]	2012–2013	Tehran	Community	RDS	FSWs	161	5	NR
	Mirzazadeh, 2016 [62] (round II)	2015	National	Facilities serving vulnerable women	MCS	FSWs	1337	2.1	0.9–4.6
	Karami, 2017 [63]	2016	Tehran	Community	TLS	FSWs	369	4.6	NR
Jordan	WHO, 2011 [33] (round I)	2009	National	Community	RDS	FSWs	225	0	NR
	MOH, 2014 [64] (round II)	2013	Amman	Community	RDS	FSWs	358	0.6	NR
	MOH, 2014 [64] (round II)	2013	Irbid	Community	RDS	FSWs	102	0	NR
	MOH, 2014 [64] (round II)	2013	Zarqa	Community	RDS	FSWs	212	0.5	NR
Lebanon	Mahfoud, 2010 [65]	2007–2008	Greater Beirut	Community	RDS	FSWs	95	0	NR
Libya	Valadez, 2013 [66] (round I)	2010–2011	Tripoli	Community	RDS	FSWs	69	15.7	3.2–32.6
Morocco	MOH, 2012 [67]	2011–2012	Agadir	Community	RDS	FSWs	364	5.1	2.1–8.6
	MOH, 2012 [67]	2011–2012	Fes	Community	RDS	FSWs	359	1.8	0–2.1
	MOH, 2012 [67]	2011–2012	Rabat	Community	RDS	FSWs	392	0	NR
	MOH, 2012 [67]	2011–12	Tanger	Community	RDS	FSWs	319	1.4	0.4–3.3
Pakistan	Bokhari, 2007 [68]	2004	Lahore	Red-light district	SyCS	FSWs	378	0.5	NR
	NACP, 2005 [38] (round I)	2005	Faisalabad	Community	RDS and TLS	Kothikhana, home, and street-based FSWs	400	0	NR
	NACP, 2005 [38] (round I)	2005	Hyderabad	Community	SyRS, RDS, and TLS	Brothel, kothikhana, home, and street-based FSWs	400	0	NR
	NACP, 2005 [38] (round I)	2005	Karachi	Community	SyRS, RDS, and TLS	Brothel, kothikhana, home, and street-based FSWs	400	0.8	NR
	NACP, 2005 [38] (round I)	2005	Lahore	Community	SyRS, RDS, and TLS	Brothel, kothikhana, home, and street-based FSWs	400	0	NR
	NACP, 2005 [38] (round I)	2005	Multan	Community	Conv (take all), RDS, and TLS	Brothel, kothikhana, home, and street-based FSWs	400	0	NR
	NACP, 2005 [38] (round I)	2005	Peshawar	Community	MCS	Kothikhana, home, and street-based FSWs	359	0	NR
	NACP, 2005 [38] (round I)	2005	Quetta	Community	RDS and MCS	Kothikhana, home, and street-based FSWs	411	0.7	NR
	NACP, 2005 [38] (round I)	2005	Sukkur	Community	RDS and TLS	Kothikhana, home, and street-based FSWs	368	0	NR
	NACP, 2007 [69] (round II)	2006	Bannu	Community	SyRS and MCS	Kothikhana, home, and street-based FSWs	194	0	NR
	NACP, 2007 [69] (round II)	2006	Faisalabad	Community	SyRS and MCS	Kothikhana, home, and street-based FSWs	400	0	NR
	NACP, 2007 [69] (round II)	2006	Gujranwala	Community	SyRS and MCS	Kothikhana, home, and street-based FSWs	400	0	NR
	NACP, 2007 [69] (round II)	2006	Hyderabad	Community	SyRS and MCS	Brothel, kothikhana, home, and street-based FSWs	398	0.3	NR
	NACP, 2007 [69] (round II)	2006	Karachi	Community	SyRS and MCS	Brothel, kothikhana, home, and street-based FSWs	403	0	NR
	NACP, 2007 [69] (round II)	2006	Lahore	Community	SyRS and MCS	Brothel, kothikhana, home, and street-based FSWs	425	0.02	NR
	NACP, 2007 [69] (round II)	2006	Larkana	Community	SyRS and MCS	Brothel, kothikhana, home, and street-based FSWs	400	0	NR
	NACP, 2007 [69] (round II)	2006	Multan	Community	SyRS and MCS	Brothel, kothikhana, home, and street-based FSWs	400	0	NR
	NACP, 2007 [69] (round II)	2006	Peshawar	Community	SyRS and MCS	Kothikhana, home, street-based, and other FSWs	423	0	NR
	NACP, 2007 [69] (round II)	2006	Quetta	Community	SyRS and MCS	Kothikhana, home, street-based, and other FSWs	398	0	NR
	NACP, 2007 [69] (round II)	2006	Sargodha	Community	SyRS and MCS	Kothikhana, home, street-based, and other FSWs	400	0	NR
	NACP, 2007 [69] (round II)	2006	Sukkur	Community	SyRS and MCS	Kothikhana, home, street-based, and other FSWs	400	0	NR
	Hawkes, 2009 [70]	2007	Abbottabad	Community	RDS	FSWs	107	0	NR
	Hawkes, 2009 [70]	2007	Rawalpindi	Community	RDS	FSWs	426	0	NR
	Khan, 2011 [71]	2007	Lahore	Community	RDS	FSWs	730	0.7	NR
	NACP, 2010 [72] (special IBBSS among FSWs)	2009	Punjab and Sindh	Community	SyRS and MCS	FSWs	2197	1.0	NR
	NACP, 2012 [40] (round IV)	2012	DG Khan	Community	SyRS and MCS	Kothikhana, home, street-based, and other FSWs	375	0.5	0.1–1.9
	NACP, 2012 [40] (round IV)	2012	Faisalabad	Community	SyRS and MCS	Kothikhana, home, street-based, and other FSWs	376	0	NR
	NACP, 2012 [40] (round IV)	2012	Haripur	Community	SyRS and MCS	Kothikhana, home, street-based, and other FSWs	211	0.9	0.3–3.4
	NACP, 2012 [40] (round IV)	2012	Karachi	Community	SyRS and MCS	Brothel, kothikhana, home, street-based, and other FSWs	377	1.9	0.9–3.8
	NACP, 2012 [40] (round IV)	2012	Lahore	Community	SyRS and MCS	Brothel, kothikhana, home, street-based, and other FSWs	375	0.5	0.1–1.9
	NACP, 2012 [40] (round IV)	2012	Larkana	Community	SyRS and MCS	Brothel, kothikhana, home, street-based, and other FSWs	375	1.9	0.9–3.8
	NACP, 2012 [40] (round IV)	2012	Multan	Community	SyRS and MCS	Brothel, kothikhana, home, street-based, and other FSWs	375	0.3	0.05–1.5
	NACP, 2012 [40] (round IV)	2012	Peshawar	Community	SyRS and MCS	Kothikhana, home, street-based, and other FSWs	367	0	NR
	NACP, 2012 [40] (round IV)	2012	Quetta	Community	SyRS and MCS	Kothikhana, home, street-based, and other FSWs	345	0	NR
	NACP, 2012 [40] (round IV)	2012	Rawalpindi	Community	SyRS and MCS	Kothikhana, home, street-based, and other FSWs	375	0	NR
	NACP, 2012 [40] (round IV)	2012	Sargodha	Community	SyRS and MCS	Brothel, kothikhana, home, street-based, and other FSWs	345	0.3	0.05–1.6
	NACP, 2012 [40] (round IV)	2012	Sukkur	Community	SyRS and MCS	Kothikhana, home, street-based, and other FSWs	375	0.8	0.3–2.3
	NACP, 2017 [42] (round V)	2016–2017	Bahawalpur	Community	SyRS and MCS	Kothikhana, home, street-based, and other FSWs	351	0	NR
	NACP, 2017 [42] (round V)	2016–2017	Bannu	Community	SyRS and MCS	Kothikhana, home, street-based, and other FSWs	196	1.5	1–4.4
	NACP, 2017 [42] (round V)	2016–2017	DG Khan	Community	SyRS and MCS	Kothikhana, home, street-based, and other FSWs	364	0.8	0.3–2.4
	NACP, 2017 [42] (round V)	2016–2017	Gujranwala	Community	SyRS and MCS	Kothikhana, home, street-based, and other FSWs	304	0.7	0.2–2.4
	NACP, 2017 [42] (round V)	2016–2017	Gujrat	Community	SyRS and MCS	Kothikhana, home, street-based, and other FSWs	250	0.4	0.1–2.2
	NACP, 2017 [42] (round V)	2016–2017	Hyderabad	Community	SyRS and MCS	Kothikhana, home, street-based, and other FSWs	364	2.2	1.1–4.3
	NACP, 2017 [42] (round V)	2016–2017	Karachi	Community	SyRS and MCS	Brothel, kothikhana, home, street-based, and other FSWs	387	2.6	1.4–4.7
	NACP, 2017 [42] (round V)	2016–2017	Kasur	Community	SyRS and MCS	Kothikhana, home, street-based, and other FSWs	364	0	NR
	NACP, 2017 [42] (round V)	2016–2017	Larkana	Community	SyRS and MCS	Brothel, kothikhana, home, street-based, and other FSWs	364	4.1	2.5–6.7
	NACP, 2017 [42] (round V)	2016–2017	Mirpurkhas	Community	SyRS and MCS	Kothikhana, home, street-based, and other FSWs	364	4.1	2.5–6.7
	NACP, 2017 [42] (round V)	2016–2017	Nawabshah	Community	SyRS and MCS	Kothikhana, home, street-based, and other FSWs	364	3.8	2.3–6.4
	NACP, 2017 [42] (round V)	2016–2017	Peshawar	Community	SyRS and MCS	Kothikhana, home, street-based, and other FSWs	265	3	1.5–5.8
	NACP, 2017 [42] (round V)	2016–2017	Quetta	Community	SyRS and MCS	Kothikhana, home, street-based, and other FSWs	364	0	NR
	NACP, 2017 [42] (round V)	2016–2017	Rawalpindi	Community	SyRS and MCS	Kothikhana, home, street-based, and other FSWs	364	0.3	0.1–1.5
	NACP, 2017 [42] (round V)	2016–2017	Sheikhupura	Community	SyRS and MCS	Kothikhana, home, street-based, and other FSWs	363	1.7	1.1–4.9
	NACP, 2017 [42] (round V)	2016–2017	Sialkot	Community	SyRS and MCS	Kothikhana, home, street-based, and other FSWs	193	0	NR
	NACP, 2017 [42] (round V)	2016–2017	Sukkur	Community	SyRS and MCS	Kothikhana, home, street-based, and other FSWs	364	8.8	6.3–12.2
	NACP, 2017 [42] (round V)	2016–2017	Turbat	Community	SyRS and MCS	Kothikhana, home, street-based, and other FSWs	72	0	NR
Somalia	Testa, 2008 [73] (round I)	2008	Hargeisa	Community	RDS	FSWs	237	5.2	2.5–8.5
	IOM, 2017 [74] (round II)	2014	Hargeisa	Community	RDS	FSWs	96	4.8	0.2–9.3
Sudan	Elkarim, 2002 [75]	2002	National	Community	MSysRS	FSWs	367	4.4	NR
	Abdelrahim, 2010 [76]	2008	Khartoum	Community	RDS	FSWs	321	0.9	0.1–2.2
	NACP, 2010 [77]	2008–09	Gezira	Community	RDS	FSWs	267	0.1	NR
	NACP, 2012 [78]	2011	Alshamalia	Community	RDS	FSWs	305	0.3	0–1
	NACP, 2012 [78]	2011	Blue Nile	Community	RDS	FSWs	279	1.5	0–3
	NACP, 2012 [78]	2011	Gadarif	Community	RDS	FSWs	282	0.6	0–1
	NACP, 2012 [78]	2011	Gezira	Community	RDS	FSWs	296	0.7	0–1
	NACP, 2012 [78]	2011	Kassala	Community	RDS	FSWs	288	5.0	2–8
	NACP, 2012 [78]	2011	Khartoum	Community	RDS	FSWs	287	0	NR
	NACP, 2012 [78]	2011	North Darfur	Community	RDS	FSWs	303	0.7	0–3
	NACP, 2012 [78]	2011	North Kordofan	Community	RDS	FSWs	296	1	0–3
	NACP, 2012 [78]	2011	Red Sea	Community	RDS	FSWs	293	7.7	4–12
	NACP, 2012 [78]	2011	River Nile	Community	RDS	FSWs	291	0.7	0–2
	NACP, 2012 [78]	2011	Sinnar	Community	RDS	FSWs	303	0.7	0–2
	NACP, 2012 [78]	2011	South Darfur	Community	RDS	FSWs	299	0.2	0–1
	NACP, 2012 [78]	2011	West Darfur	Community	RDS	FSWs	284	1	0–3
	NACP, 2012 [78]	2011	White Nile	Community	RDS	FSWs	288	1.3	0–3
	MOH, 2016 [79]	2015–2016	Juba, South Sudan	Community	RDS	FSWs	835	37.9	33.6–42.2
Tunisia	Hsairi, 2012 [80]	2009	Tunis, Sfax, and Sousse	Community	RDS	Street-based FSWs	703	0.4	NR
	Hsairi, 2012 [80]	2011	Tunis	Community	TLS	Street-based FSWs	357	0.6	0–1.3
	Hsairi, 2012 [80]	2011	Sfax	Community	TLS	Street-based FSWs	284	0	NR
	Hsairi, 2012 [80]	2011	Sousse	Community	TLS	Street-based FSWs	347	1.2	0.02–2.3
Yemen	Stulhofer, 2008 [81] (round I)	2008	Aden	Community	RDS	FSWs	244	1.3	0–2.9
	MOH, 2014 [82] (round I)	2010–2011	Hodeida	Community	RDS	FSWs	301	0	NR

The table is sorted by year(s) of data collection

*Abbreviations*: *CI* confidence interval, *Conv* convenience, *FSWs* female sex workers, *IBBSS* integrated bio-behavioral surveillance survey, *IOM* International Organization for Migration, *MCS* multistage cluster sampling, *MOH* Ministry of Health, *MSyRS* multistage systematic random sampling, *NACP* National AIDS Control Programme, *NR* not reported, *RDS* respondent-driven sampling, *SAR AIDS HDS* South Asia Region AIDS Human Development Sector, *SyCS* systematic cluster sampling, *SyRS* systematic random sampling, *TLS* time-location sampling, *WHO* World Health Organization

*The decimal places of the prevalence figures are as reported in the original reports, but prevalence figures with more than one decimal places were rounded to one decimal place, with the exception of those below 0.1%. Most studies did not report the 95% CIs associated with prevalence

**Integrated bio-behavioral surveillance survey with sampling initially planned as respondent-driven but ended up being a convenience for logistical reasons

**Table 3:** HIV prevalence in FSWs in the Middle East and North Africa (MENA), as reported in studies using non-probability sampling

Country	Author, year [citation]	Year(s) of data collection	City/province	Study site	Sampling	Population	Sample size	HIV prevalence*	
								%	95% CI
Afghanistan	Todd, 2010 [83]	2006–2008	Jalalabad, Kabul, and Mazar-i-Sharif	Community and NGO	Conv	FSWs	520	0.2	0.01–1.1
Djibouti	Rodier, 1993 [84]	1987	Djibouti	STI clinic	Conv	Street-based FSWs	66	4.6	NR
	Rodier, 1993 [84]	1987	Djibouti	STI clinic	Conv	Bar hostesses	221	1.4	NR
	Constantine, 1992 [52]	1988	Djibouti	NR	Conv	FSWs	33	18.2	NR
	Rodier, 1993 [84]	1988	Djibouti	STI clinic	Conv	Street-based FSWs	78	9.0	NR
	Rodier, 1993 [84]	1988	Djibouti	STI clinic	Conv	Bar hostesses	255	2.7	NR
	Rodier, 1993 [84]	1990	Djibouti	STI clinic	Conv	Street-based FSWs	116	41.7	NR
	Rodier, 1993 [84]	1990	Djibouti	STI clinic	Conv	Bar hostesses	180	5.0	NR
	Couzineau, 1991 [85]	1991	Djibouti	STI clinic	Conv	Street-based FSWs	300	43	NR
	Couzineau, 1991 [85]	1991	Djibouti	STI clinic	Conv	Bar girls	397	13.1	NR
	Rodier, 1993 [84]	1991	Djibouti	STI clinic and residences	Conv	Street-based FSWs	292	36.0	NR
	Rodier, 1993 [84]	1991	Djibouti	STI clinic and residences	Conv	Bar hostesses	360	15.3	NR
	Philippon, 1997 [86]	1995	Djibouti	STI clinic	Conv	Street-based FSWs	176	49	NR
	Marcelin, 2002 [87]	1998–1999	Djibouti	STI clinics	Conv	Street-based FSWs	43	70	NR
	Marcelin, 2002 [87]	1998–1999	Djibouti	STI clinics	Conv	FSWs working in luxury bars	123	7	NR
Egypt	Sheba, 1988 [88]	1986–1987	Multiple cities	NR	Conv	FSWs	87	0	NR
	Watts, 1993 [89]	1986–1990	Urban areas	Medical facilities	Conv	FSWs	349	0	NR
	Kabbash, 2012 [90]	2009–2010	Greater Cairo	Community	Conv	FSWs	431	0	NR
Iran	Jahani, 2005 [91]	2002	NR	Detainment center/prison	Conv	FSWs detained by the police	149	0	NR
	Kassaian, 2012 [92]	2009–2010	Isfahan	Prison, drop-in centers, and community	Conv	FSWs	91	0	NR
	Taghizadeh, 2015 [93]	2014	Sari, Mazandaran	Drop-in center	Conv	FSWs at a drop-in center	184	4	NR
	Asadi-Ali, 2018 [94]	2015	Northern Iran	Counseling center, drop-in center, and community	Conv	FSWs	133	1.5	NR
Lebanon	Naman, 1989 [95]	1985–1987	NR	NR	Conv	FSWs	291	0.3	NR
Morocco	MOH, 2008 [96]	2007	Agadir, Rabat/Sale, Tanger	NGO clinic	Conv	FSWs presenting for consultation	141	1.4	0.1–2.5
Pakistan	Iqbal, 1996 [97]	1987–1994	Lahore	Hospital	Conv	FSWs	21	0	NR
	Baqi, 1998 [98]	1993–1994	Karachi	VCT	Conv	FSWs in red-light district	77	0	NR
	Anwar, 1998 [99]	NR	Lahore	NR	NR	FSWs	103	1.9	NR
	Bokhari, 2007 [68]	2004	Karachi	Community	Snowball	FSWs in red-light district	421	0	NR
	Shah, 2004 [100]	2004	Hyderabad	Community	Conv	FSWs	157	0	NR
	Shah, 2004 [101]	2004	Sindh	Sentinel surveillance	Conv	FSWs	163	1.2	NR
	Akhtar, 2008 [102]	2007	Faisalabad	Community	NR	FSWs	246	0	NR
	Raza, 2015 [103]	2014	Rawalpindi	Clinics	Conv	FSWs	NR	0	NR
Somalia	Jama, 1987 [104]	1985–1986	Mogadishu	Camp	Conv	FSWs attending health education program	85	0	NR
	Burans, 1990 [105]	NR	Mogadishu	NR	Conv	FSWs	89	0	NR
	Scott, 1991 [106]	1989	Merka, Kismayu	NR	Conv	FSWs	57	0	NR
	Corwin, 1991 [107]	1990	Chismayu, Merca, Mogadishu	NR	Conv	FSWs	302	3	NR
	Jama Ahmed, 1991 [51]	1991	Mogadishu	PHC	Conv	FSWs	155	0.6	NR
Sudan	Burans, 1990 [108]	1987	Port Sudan	NR	Conv	FSWs	203	0	NR
	McCarthy, 1995 [109]	NR	Juba, South Sudan	NR	Conv	FSWs	50	16	NR
Tunisia	Bchir, 1988 [110]	1987	Sousse	NR	Conv	FSWs	42	0	NR
	Hassen, 2003 [111]	NR	Sousse	PHC	Conv	Legal FSWs	51	0	NR
	Znazen, 2010 [112]	2007	Tunis, Sousse, and Gabes	Medical facilities	Conv	Legal FSWs undergoing routine testing	183	0	NR

The table is sorted by year(s) of data collection or year of publication if the year of data collection was not reported

*Abbreviations*: *CI* confidence interval, *Conv* convenience, *FSWs* female sex workers, *MOH* Ministry of Health, *NGO* non-governmental organization, *NR* not reported, *PHC* primary healthcare centers, *STI* sexually transmitted infection, *VCT* voluntary counseling and testing

*The decimal places of the prevalence figures are as reported in the original reports, but prevalence figures with more than one decimal places were rounded to one decimal place, with the exception of those below 0.1%. Most studies did not report the 95% CIs associated with prevalence

In clients/male STI clinic attendees, HIV prevalence ranged from 0 to 34.6%, with a median of 0.4% (Table 4). Studies also showed high heterogeneity with 37.7% reporting zero prevalence. The median prevalence was 0% (range = 0–1.1%), 0.6% (range = 0–9.6%), and 7.4% (range = 0.8–34.6%) in countries with low-level, intermediate-intensity, and concentrated epidemics, respectively. Ranges indicated pockets of higher HIV prevalence in countries with intermediate-intensity epidemics.

**Table 4:** HIV prevalence in clients of FSWs (or proxy populations of clients of FSWs such as male STI clinic attendees) in the Middle East and North Africa (MENA)

Country	Author, year [citation]	Year(s) of data collection	City/province	Study site	Sampling	Population	Sample size	HIV prev*		Sexual contacts
								%	95% CI	
Algeria	MOH, 2009 [113]	2004	Oran	Sent. surv.	Conv	STI clinic attendees	41	4.9	NR	NR
	MOH, 2009 [113]	2004	Tamanrasset	Sent. surv.	Conv	STI clinic attendees	105	0	0	NR
	MOH, 2009 [113]	2004	Tizi-Ouzou	Sent. surv.	Conv	STI clinic attendees	11	9.1	NR	NR
	MOH, 2009 [113]	2007	National	Sent. surv.	Conv	STI clinic attendees	571	3.3	NR	NR
Djibouti	Rodier, 1993 [84]	1987	Djibouti	STI clinic	Conv	STI clinic attendees	252	0.8	NR	NR
	Rodier, 1993 [84]	1988	Djibouti	STI clinic	Conv	STI clinic attendees	249	0.8	NR	NR
	Fox, 1989 [114]	NR	NR	NR	Conv	Clients of FSWs	105	1.0	NR	Clients of FSWs
	Rodier, 1993 [84]	1990	Djibouti	STI clinic	Conv	STI clinic attendees	106	1.9	NR	NR
	OMS, 2001 [115]	1990	NR	STI clinic	Conv	STI clinic attendees	NR	2.2	NR	NR
	Rodier, 1993 [84]	1991	Djibouti	STI clinic	Conv	STI clinic attendees	193	10.4	NR	NR
	OMS, 2001 [115]	1991	NR	STI clinic	Conv	STI clinic attendees	NR	9.2	NR	NR
	MOH, 1993 [116]	1992	NR	Sent. surv.	Conv	STI clinic attendees	NR	11.6	NR	NR
	MOH, 1993 [116]	1993	NR	Sent. surv.	Conv	STI clinic attendees	411	14.4	NR	NR
	MOH, 2002 [117]	2001–2002	Djibouti	STI clinic	Conv	STI clinic attendees	237	34.6	NR	NR
	Bortolotti, 2007 [6, 118]	2006	Djibouti	STI clinic	Conv	STI clinic attendees	72	5.6	1.5–13.6	NR
Egypt	Sheba, 1988 [88]	1986–1987	Multiple cities	STI clinic	Conv	STI clinic attendees	302	0	NR	NR
	Sadek, 1991 [119]	1987–1988	Cairo	STI clinic	Conv	STI clinic attendees	140	0.7	NR	NR
	Sadek, 1991 [119]	1989–1990	Cairo	STI clinic	Conv	STI clinic attendees	125	0.8	NR	NR
	Fox, 1994 [120]	1993	Alexandria	STI clinic	Conv	STI clinic attendees	200	0	NR	NR
	Fox, 1994 [120]	1993	Cairo	STI clinic	Conv	STI clinic attendees	300	0.3	NR	NR
	Saleh, 2000 [121]	1998–2000	Alexandria	STI clinic	Conv	STI clinic attendees	295	0	NR	NR
Kuwait	NAP, 1999 [122]	1984–1998	Sabah, Kuwait	STI clinic	Conv	STI clinic attendees	3097	0.02	NR	NR
	Murzi, 1989 [123]	1988	Kuwait	STI clinic	Conv	STI clinic attendees	305	0	NR	NR
	Al-Owaish, 2000 [124]	1996–1997	Kuwait	STI clinic	SyRS	STI clinic attendees (Kuwaiti)	617	0	NR	23% reported contact with FSWs, 1% with MSWs, 35% with girlfriend, 12% with a mix of the above
	Al-Owaish, 2000 [124]	1996–1997	Kuwait	STI clinic	SyRS	STI clinic attendees (non-Kuwaiti)	1367	0	NR	61% reported contact with FSWs, 0.5% with MSWs, 28.5% with girlfriend, 3% with a mix of the above
	Al-Owaish, 2002 [125]	2002	Kuwait	STI clinic	Conv	STI clinic attendees (non-Kuwaiti)	599	0	NR	NR
	Al-Mutairi, 2007 [126]	2003–2004	Kuwait	STI clinic	Conv	STI clinic attendees (predom. men)	520	0	NR	79% reported contact with FSWs
Morocco	Heikel, 1999 [127]	1992–1996	Casablanca	STI clinic	Conv	STI clinic attendees	1131	0.9	NR	NR
	Manhart, 1996 [128]	1996	Agadir, Tanger, and Marrakech	STI clinic	Conv	STI clinic attendees	223	1.4	NR	NR
	Alami, 2002 [129]	2001	Rabat, Sale, Beni Mellal, and Marrakech	Sent. surv.	Conv	STI clinic attendees	422	0	NR	70.7% reported new sexual partner, 47% multiple sexual partners in the past 3 months
	MOH, 2001 [130]	2001	Marrakech, Beni Mellal, and Rabat, Sale	Sent. surv.	Conv	STI clinic attendees	422	0	NR	NR
	Khattabi, 2005 [131]	2004	National	Sent. surv.	Conv	STI clinic attendees	NR	0.4	NR	NR
	MOH, 2013 [132]	2006	National	Sent. surv.	Conv	STI clinic attendees	1180	0.2	NR	NR
	MOH, 2013 [132]	2007	National	Sent. surv.	Conv	STI clinic attendees	986	0.4	NR	NR
	MOH, 2013 [132]	2008	National	Sent. surv.	Conv	STI clinic attendees	1237	0.5	NR	NR
	MOH, 2013 [132]	2009	National	Sent. surv.	Conv	STI clinic attendees	1103	0.3	NR	NR
	MOH, 2013 [132]	2010	National	Sent. surv.	Conv	STI clinic attendees	1181	0.7	NR	NR
	MOH, 2013 [133]	2011	Fes, Meknes, and Laayoune Boujdour	VCT	Conv	STI clinic attendees	88	2.3	NR	NR
	MOH, 2013 [132]	2012	National	Sent. surv.	Conv	STI clinic attendees	1070	0.3	NR	NR
	MOH, 2013 [133]	2012	National	VCT and STI clinic	Conv	STI clinic attendees	1297	0.4	NR	NR
Pakistan	Mujeeb, 1993 [134]	NR	Karachi	STI clinic	Conv	STI clinic attendees	32	0	NR	NR
	Memon, 1997 [135]	1994–1995	Hyderabad	STI clinic	Conv	STI clinic attendees (predom. men)	50	0	NR	NR
	NAP, 1996 [136]	1995	Karachi	STI clinic	Conv	STI clinic attendees (predom. men)	402	0	NR	NR
	NAP, 1996 [136]	1995	Lahore	STI clinic	Conv	STI clinic attendees (predom. men)	295	0	NR	NR
	Rehan, 2003 [137]	1999	Karachi	STI clinic	Conv	STI clinic attendees	138	0	NR	43% reported contact with FSWs, 12% with casual heterosexual contact, 11.6% with MSM, 18.4% reported bisexuality
	Rehan, 2003 [137]	1999	Lahore	STI clinic	Conv	STI clinic attendees	148	0	NR	NR
	Rehan, 2003 [137]	1999	Peshawar	STI clinic	Conv	STI clinic attendees	93	1.1	NR	NR
	Rehan, 2003 [137]	1999	Quetta	STI clinic	Conv	STI clinic attendees	86	0	NR	NR
	Bhutto, 2011 [138]	2000–2009	Larkana	STI clinic	Conv	STI clinic attendees	4288	0.06	NR	83% reported a history of contact with FSWs
	Bokhari, 2007 [68]	2004	Karachi	Trucking agencies	SRS	Truck driver clients of FSWs	120	0	NR	Subsample including only clients of FSWs
	Razvi, 2014 [139]	2010–2014	Abbottabad	STI clinic	Conv	STI clinic attendees	465	1.1	NR	8% refused to answer, 70% of the rest reported contact with FSWs, 21% with MSM, 7.5% with married women
	NAP, 2012 [140]	2011	Balochistan	Mines	SRS	Mine workers clients of FSWs	381	0	NR	Subsample including only men reporting contact with FSWs at last sex
Somalia	Ismail, 1990 [141]	1986	Mogadishu	STI clinic	Conv	STI clinic attendees	101	0	NR	54% reported contact with FSWs
	Scott, 1991 [106]	1989	Mogadishu	STI clinic	Conv	STI clinic attendees	50	0	NR	NR
	Burans, 1990 [105]	NR	Mogadishu	NR	Conv	STI clinic attendees (80% soldiers)	45	0	NR	40% reported contact with FSWs
	Corwin, 1991 [107]	1990	Chismayu, Merca, and Mogadishu	NR	Conv	Partners of FSWs	26	0	NR	Partners of FSWs
	Duffy, 1999 [142]	1999	Hargeisa	Sent. surv.	Conv	STI clinic attendees	106	0.9	NR	NR
	WHO, 2005 [143]	2004	Bossasso	Sent. surv.	Conv	STI clinic attendees	78	1.3	NR	NR
	WHO, 2005 [143]	2004	Hargeisa	Sent. surv.	Conv	STI clinic attendees	52	9.6	NR	NR
	WHO, 2005 [143]	2004	Mogadishu	Sent. surv.	Conv	STI clinic attendees	46	4.4	NR	NR
	UNHCR, 2007 [144]	2006–2007	Dadaab refugee camp	STI clinic	Conv	STI clinic attendees	199	0.5	NR	NR
	Ismail, 2007 [145]	2007	Hargeisa	STI clinic	Conv	STI clinic attendees	108	7.4	NR	NR
	NAP, 2010 [146]	2007	Puntland	Sent. surv.	Conv	STI clinic attendees	NR	1.5	NR	NR
Sudan	McCarthy, 1989 [147]	1987	Port Sudan and Suakin	NR	Conv	Clients of FSWs	157	0	NR	Subsample including only clients of FSWs
	McCarthy, 1989 [148]	1987–1988	Gederef, Port Sudan, Kassala, Omdurman, and Juba	Outpatient military clinics	Conv	Soldiers clients of FSWs	398	2.5	NR	Subsample including only soldiers reporting a history of contact with FSWs
	McCarthy, 1995 [109]	NR	Juba, South Sudan	STI clinics	Conv	STI clinic attendees clients of FSWs	37	13.5	NR	Subsample including only men reporting contact with FSWs in the past 10 years
	US Cens. Bureau, 2017 [149]	2004	Khartoum	Sent. surv.	Conv	STI clinic attendees	72	1.4	NR	NR
	US Cens. Bureau, 2017 [149]	2004	Red Sea	Sent. surv.	Conv	STI clinic attendees	164	1.8	NR	NR
Yemen	Abdol-Quauder, 1993 [150]	1992	Sanaa	STI clinic	Conv	STI clinic attendees	30	0	NR	NR

The table is sorted by year(s) of data collection or year of publication if the year of data collection was not reported

*Abbreviations*: *Cens* Census, *CI* confidence interval, *Conv* convenience, *FSWs* female sex workers, *MENA HIV ESP* MENA HIV/AIDS Epidemiology Synthesis Project, *MOH* Ministry of Health, *NAP* National AIDS Program, *NR* not reported, *OMS* Organisation Mondiale de la Sante, *Predom.* predominantly, *Prev* prevalence, *Sent. surv.* sentinel surveillance, *SRS* simple random sampling, *STI* sexually transmitted infection, *SyRS* systematic random sampling, *UNHCR* United Nations Higher Commission for Refugees, *VCT* voluntary counseling and testing, *WHO* World Health Organization

*The decimal places of the prevalence figures are as reported in the original reports, but prevalence figures with more than one decimal places were rounded to one decimal place, with the exception of those below 0.1%. Most studies did not report the 95% CIs associated with prevalence

### Quality assessment

Additional file 1: Tables S6-S9 show the summarized and study-specific quality assessments for the size estimation and HIV prevalence studies in FSWs and clients. Almost all size estimation studies used clear/valid sex work definitions, and > 70% used rigorous size estimation methodologies. Similarly, > 70% of prevalence studies in FSWs used clear/valid sex work definitions and probability-based sampling for participants’ recruitment. Meanwhile, > 85% of prevalence studies in clients used convenience sampling.

Overall, studies were of reasonable quality. The majority of size estimation studies in FSWs and clients had low ROB on ≥ 2 quality domains (94.4% and 82.1%, respectively), and none had high ROB on ≥ 2 domains. Similarly, 85.0% of prevalence studies in FSWs and 39.4% of studies in clients had low ROB on ≥ 2 domains (studies among STI clinic attendees mostly used convenience sampling, and few reported on contact with FSWs), while 0.7% and 6.1% had high ROB on ≥ 2 domains, respectively.

### Pooled mean HIV prevalence

The pooled mean HIV prevalence for the MENA region was 1.4% (95% confidence interval (CI) = 1.1–1.8%) in FSWs and 0.4% (95% CI = 0.1–0.7%) in clients (Table 5). A difference was observed between the median prevalence and the pooled mean prevalence due to the high clustering of prevalence measures close to zero.

**Table 5:** Results of meta-analyses on studies reporting HIV prevalence in FSWs and their clients (or proxy populations of clients such as male STI clinic attendees) in the Middle East and North Africa (MENA) by epidemic type

		Country	Studies (*N*)	Samples		HIV prevalence		Pooled mean HIV prevalence**		Heterogeneity measures		
				Tested	HIV positive	Median* (%)	Range* (%)	%	95% CI	*Q* (*p* value)^†^	*I*^2‡^ (%; 95% CI)	Prediction interval^£^ (95%)
FSWs	Low-level^⁑^	Afghanistan	9	3578	7	0	0–0.90	0.03	0.00–0.18	7.59 (*p* = 0.4744)	0.0 (0.0–62.9)	0.00–0.22
		Bahrain	1	724	6	0.83	–	0.83^¥^	0.30–1.80	–	–	–
		Egypt	33	7222	16	0	0–1.49	0.03	0.00–0.14	36.26 (*p* = 0.2765)	12.8 (0.0–43.4)	0.00–0.34
		Iran	32	17,277	211	0.02	0–14.00	0.99	0.34–1.88	569.63 (*p* < 0.0001)	94.6 (93.2–95.6)	0.00–8.84
		Iraq	29	15,852	1	0	0–0.07	0.00	0.00–0.00	6.24 (*p* = 1.0000)	0.0 (0.0–0.0)	0.00–0.00
		Jordan	7	1024	4	0	0–1.33	0.00	0.00–0.31	3.43 (*p* = 0.7537)	0.0 (0.0–48.9)	0.00–0.48
		Lebanon	11	11,589	12	0.07	0–2.40	0.00	0.00–0.07	18.82 (*p* = 0.0426)	46.9 (0.0–73.6)	0.00–0.33
		Pakistan	81	26,678	217	0	0–8.80	0.35	0.18–0.57	368.57 (*p* < 0.0001)	78.3 (73.3–82.3)	0.00–3.06
		Syria	56	97,071	12	0	0–0.20	0.00	0.00–0.00	32.37 (*p* = 0.9936)	0.0 (0.0–0.0)	0.00–0.00
		Tunisia	53	22,224	59	0	0–2.30	0.02	0.00–0.11	124.81 (*p* < 0.0001)	58.3 (43.6–69.2)	0.00–0.89
		Yemen	10	1767	34	0.25	0–7.00	0.82	0.00–2.91	63.01 (*p* < 0.0001)	85.7 (75.6–91.7)	0.00–11.67
	Intermediate-intensity^⁑^	Algeria	33	4241	179	2.00	0–20.00	2.39	1.02–4.15	215.22 (*p* < 0.0001)	85.1 (80.1–88.9)	0.00–15.05
		Libya	4	1249	28	8.43	1.08–18.18	4.86	0.81–11.37	34.41 (*p* < 0.0001)	91.3 (80.8–96.0)	0.00–47.09
		Morocco	200	40,507	804	1.07	0–52.90	1.11	0.83–1.41	851.66 (*p* < 0.0001)	76.6 (73.3–79.6)	0.00–5.98
		Somalia	17	2015	57	0.35	0–47.06	1.64	0.42–3.39	61.50 (*p* < 0.0001)	74.0 (57.7–83.8)	0.00–10.24
		Sudan^€^	22	7207	128	0.95	0–7.70	1.30	0.76–1.96	98.06 (*p* < 0.0001)	78.6 (68.1–85.6)	0.00–5.26
	Concentrated ^⁑^	Djibouti	68	22,028	4618	18.75	0–70.00	17.89	13.62–22.60	5127.36 (*p* < 0.0001)	98.7 (98.6–98.8)	0.00–63.91
		South Sudan	8	5466	1108	18.50	2.82–37.90	17.32	8.66–28.14	554.81 (*p* < 0.0001)	98.7 (98.3–99.1)	0.00–61.99
		All countries	674	287,719	7501	0.26	0–70.00	1.44	1.14–1.76	24,605.29 (*p* < 0.0001)	97.3 (97.2–97.4)	0.00–16.49
Clients of FSWs	Low-level^⁑^	Egypt	6	1362	3	0.17	0–0.80	0.09	0.00–0.42	4.82 (*p* = 0.4386)	0.0 (0.0–73.7)	0.00–0.60
		Kuwait	6	6505	1	0	0–0.02	0.00	0.00–0.04	0.36 (*p* = 0.9963)	0.0 (0.0–0.0)	0.00–0.07
		Pakistan	12	6498	9	0	0–1.10	0.00	0.00–0.10	14.93 (*p* = 0.1857)	26.3 (0.0–62.6)	0.00–0.42
		Yemen	1	30	0	0	–	0.00^¥^	0.00–11.57	–	–	–
	Intermediate-intensity^⁑^	Algeria	7	728	22	7.29	0–25.80	3.51	0.32–8.90	39.79 (*p* < 0.0001)	84.9 (70.8–92.2)	0.00–27.63
		Morocco	84	10,348	47	0	0–8.00	0.00	0.00–0.05	76.30 (*p* = 0.6854)	0.0 (0.0–19.9)	0.00–0.05
		Somalia	11	1010	21	0.94	0–9.62	1.38	0.25–3.11	25.74 (*p* = 0.0041)	61.1 (25.0–79.9)	0.00–8.46
		Sudan^€^	4	791	14	1.61	0–2.51	1.22	0.16–2.97	7.02 (*p* = 0.0711)	57.3 (0.0–85.8)	0.00–11.65
	Concentrated^⁑^	Djibouti	15	2222	217	2.20	0–34.60	5.36	1.53–10.81	244.98 (*p* < 0.0001)	94.3 (92.0–95.9)	0.00–35.23
		South Sudan	1	37	5	13.5	–	13.5^¥^	4.54–28.77	–	–	–
		All countries	147	29,531	339	0	0–34.60	0.38	0.14–0.71	977.96 (*p* < 0.0001)	85.1 (82.9–87.0)	0.00–6.60

*Abbreviations*: *CI* confidence interval, *FSWs* female sex workers

*These medians and ranges are calculated on the stratified HIV prevalence measures

**Missing sample sizes for measures (or their strata) were imputed using median sample size calculated from studies with available information. Analyses excluding these studies had no impact on study findings

^†^*Q*—the Cochran’s *Q* statistic is a measure assessing the existence of heterogeneity in effect size (here, HIV prevalence) across studies

^‡^*I*^2^—a measure assessing the magnitude of between-study variation that is due to the differences in effect size (here, HIV prevalence) across studies rather than chance

^£^Prediction interval—a measure estimating the 95% interval of the distribution of true effect sizes (here, HIV prevalence)

^⁑^Based on results of meta-analyses for FSWs, countries were classified as having low-level HIV epidemic (prevalence < 1%), intermediate-intensity HIV epidemic (prevalence 1–5%), and concentrated HIV epidemic (prevalence > 5%)

^¥^Point estimate as only one study was available

^€^Before 2011, South Sudan was part of Sudan, and thus, earlier measures from Sudan were based on studies that may have included participants from both Sudan and South Sudan

In FSWs, the national-level pooled mean prevalence was 0 or < 1% in most countries (low-level epidemics); between 1 and 5% (intermediate-intensity epidemics) in Algeria, Libya, Morocco, Somalia, and Sudan; and > 5% (concentrated epidemics) in Djibouti (17.9%, 95% CI = 13.6–22.6%) and South Sudan (17.3%, 95% CI = 8.7–28.1%).

In clients/male STI clinic attendees, the national-level pooled mean prevalence was mostly 0 or < 1%. However, high prevalence was estimated in Djibouti (5.4%, 95% CI = 1.5–10.8%) and South Sudan (13.5%, 95% CI = 4.5–28.8%).

There was evidence for the heterogeneity in effect size (prevalence) in meta-analyses. *p* value for Cochran’s *Q* statistic was mostly < 0.0001, prediction intervals were wide, and *I*^2^ was often > 50% indicating that most between-study variability is due to the true differences in prevalence across studies rather than chance.

### Associations with prevalence, sources of between-study heterogeneity, and temporal trend

Univariable meta-regressions for FSWs demonstrated strong evidence for an association with odds for subregion, population type, sample size, year of data collection, and response rate (Table 6). Meanwhile, there was poor evidence for an association with sampling methodology, validity of sex work definition, and HIV ascertainment, which were hence dismissed from inclusion in the multivariable model. Most variability in odds was explained by subregion (adjusted *R*^2^ = 39.8%).

**Table 6:** Results of meta-regression analyses to identify associations with HIV prevalence, sources of between-study heterogeneity, and trend in HIV prevalence in FSWs in the Middle East and North Africa (MENA)

Variables		Studies	Samples	Univariable analyses			Multivariable analysis		
		Total *N*	Total *N*	OR (95% CI)	LR test *p* value^€^	Variance explained R^2£^ (%)	AOR (95% CI)	*p* value	LR test *p* value^¥^
Country/subregion*									
Eastern MENA	Afghanistan, Iran, Pakistan	122	47,533	1.00	< 0.001	39.80	1.00		< 0.001
Fertile Crescent	Egypt, Iraq, Jordan, Lebanon, Syria	136	132,758	0.17 (0.10–0.27)			0.21 (0.12–0.36)	< 0.001	
Bahrain and Yemen	Bahrain and Yemen	11	2491	2.60 (0.78–8.67)			1.77 (0.52–6.01)	0.357	
Horn of Africa	Djibouti, Somalia, South Sudan	93	29,509	33.45 (19.77–56.58)			45.43 (24.66–83.68)	< 0.001	
North Africa	Algeria, Libya, Morocco, Sudan, Tunisia	312	75,428	3.14 (2.09–4.72)			2.90 (1.80–4.68)	< 0.001	
Population type	Street-based, venue-based, and other FSWs^†^	619	220,363	1.00	0.002	1.29	1.00		0.163
	Bar girls	55	67,356	0.33 (0.17–0.67)			0.66 (0.37–1.18)	0.163	
Total sample size of tested FSWs	< 100 participants	75	4008	1.00	0.001	1.54	1.00		< 0.001
	≥ 100 participants	599	283,711	0.36 (0.20–0.65)			0.35 (0.21–0.56)	< 0.001	
Median year of data collection**	< 1993	104	36,038	1.00	0.001	1.96	1.00		0.005
	1993–2002	169	98,221	0.31 (0.17–0.56)			1.18 (0.71–1.95)	0.522	
	≥ 2003	401	153,460	0.57 (0.33–0.97)			2.03 (1.24–3.33)	0.005	
Sampling methodology	Non-probability sampling	570	254,072	1.00	0.217	0.08	–	–	–
	Probability-based sampling	104	33,647	0.72 (0.42–1.21)			–	–	–
Response rate	≥ 60%	96	31,161	1.00	0.043	0.64	1.00		0.544
	< 60%/unclear	62	14,102	2.76 (1.24–6.13)			1.17 (0.60–2.27)	0.645	
	Not applicable^‡^	516	242,456	1.37 (0.80–2.37)			1.33 (0.79–2.23)	0.279	
Validity of sex work definition	Clear and valid definition	117	36,431	1.00	0.161	0.25	–	–	–
	Poorly defined/unclear	41	8832	2.35 (0.96–5.73)			–	–	–
	Not applicable^‡^	516	242,456	1.15 (0.70–1.90)			–	–	–
HIV ascertainment	Biological assays	157	44,894	1.00	0.786	0	–	–	–
	Self-report, unclear, and not applicable^‡^	517	242,825	0.94 (0.60–1.47)			–	–	–

*Abbreviations*: *AOR* adjusted odds ratio, *CI* confidence interval, *FSWs* female sex workers, *LR* likelihood ratio, *OR* odds ratio

*Countries were grouped based on geography and similarity in HIV prevalence levels. Given the large fraction of studies with zero HIV prevalence, particularly in the Fertile Crescent, an increment of 0.1 was added to a number of events in all studies when generating log odds, and Eastern MENA was thus used also as a statistically better reference. While this choice of increment was arbitrary, other increments yielded the same findings, though some of the effect sizes changed in scale

**Year grouping was driven by independent evidence identifying the emergence of HIV epidemics among both men who have sex with men [10] and people who inject drugs [11] in multiple MENA countries around 2003. Missing values for year of data collection (only six stratified measures) were imputed using data for year of publication adjusted by the median difference between year of publication and median year of data collection (for studies with complete information)

^†^A large fraction of studies did not separate the different forms of female sex workers, and thus it was not possible to analyze these as separate categories

^‡^Measures extracted only from routine databases with no reports describing the study methodology were not included in the ROB assessment

^€^Predictors with *p* value ≤ 0.1 were considered as showing strong evidence for an association with (prevalence) odds and were hence included in the multivariable analysis

^£^Adjusted *R*^2^ in the final multivariable model = 49.21%

^¥^Predictors with *p* value ≤ 0.1 in the multivariable model were considered as showing strong evidence for an association with (prevalence) odds

Multivariable analysis indicated strong subregional differences and explained 49.2% of the variation (Table 6). Compared to Eastern MENA, the adjusted odds ratio (AOR) ranged from 0.2 (95% CI = 0.1–0.4) for the Fertile Crescent to 45.4 (95% CI = 24.7–83.7) for the Horn of Africa. Studies with a larger sample size (≥ 100) showed lower odds (AOR = 0.4, 95% CI = 0.2–0.6).

Compared with studies with data collection pre-1993, studies conducted after 2003 showed strong evidence for higher odds (AOR = 2.0, 95% CI = 1.2–3.3). Notably, the trend of increasing odds was evident only after controlling for the strong confounding effect of the subregion. The trend for each subregion was also overall increasing, though the strength of evidence varied across subregions (not shown). Including the year of data collection as a linear term, instead of a categorical variable, using only post-2003 data indicated strong evidence for increasing HIV odds (AOR = 1.15, 95% CI = 1.09–1.21, *p* < 0.0001; not shown). No association was found with the population type or response rate.

Meta-regression analyses for clients demonstrated similar results to those of FSWs, but with wider CIs considering the smaller number of prevalence studies (Additional file 1: Table S10). There was evidence that subregion was associated with HIV odds in clients, but no evidence that sample size or year of data collection explained the between-study heterogeneity.

### Sex work context and sexual and injecting risk behaviors

For the detailed sex work context and behavioral measures, we provide here (for brevity) only a high-level summary of key measures.

#### Sex work context

Across studies, the mean age of FSWs ranged from 19.5 to 37.4, with a median of 27.8 years. Mean age at sexual debut ranged from 14.0 to 22.5 years (median = 17.5), and mean age at sex work initiation ranged from 17.5 to 27.5 years (median = 22.7). Mean duration of sex work ranged from 0.7 to 14.3 years (median = 5.5). A median of 28.0% (range = 0.9–76.6%) of FSWs were single, 30.1% (range = 0–65.5%) were divorced, and 7.0% (range = 0–27.2%) were widowed.

#### Reported condom use

There was high heterogeneity in reported condom use among FSWs by sexual partnership type and across and within countries (Additional file 1: Table S11). Condom use at last sex with clients ranged from 1.2 to 94.8% (median = 44.0%). Consistent condom use with clients ranged from 0 to 95.2% (median = 26.3%) among all FSWs and from 38.2 to 45.3% (median = 42.3%) among FSWs reporting condom use with clients.

Median condom use at last sex with regular clients was 55.9% (range = 25.5–92.0%) and that with one-time clients was 58.3% (range = 28.5–96.0%). Less condom use at last sex was found with non-paying partners (median = 22.0%, range = 4.9–78.3%). There was also variability in condom use at last *anal* sex (range = 0–86.5%), though low levels were generally reported (median = 18.5%).

The median fraction of FSWs who reported having a condom at the time of study interview was 12.5% (range = 0–66.1%).

#### Clients and partners

Studies varied immensely in types of measures reporting data on clients and partners. Some reported a mean number of regular/non-regular clients, but over various time frames. Others reported different distributions for the number of clients (and by client type), also over various time frames. Summarizing the evidence was therefore challenging, given the large type of measure variability.

This being said, the mean number of clients in the past month ranged from 4.4 to 114.0, with a median of 34.0 clients. Median fraction of FSWs reporting (during the past month) < 5 clients, 5–9 clients, and 10+ clients was 28.5%, 28.1%, and 19.1%, respectively. FSWs were equally likely to report regular and one-time clients during the past month (medians = 80.0% and 81.0%, ranges = 54.3–92.4% and 59.2–97.5%, respectively).

FSWs reported a distribution of sex acts in the past week, with a median of 41.2% reporting 1–2 acts, 32.0% reporting 3–4 acts, and 12.9% reporting 5+ acts. Anal sex with clients in the past month was reported by a median of 8.0% (range = 2.3–100%).

Median fraction of FSWs that are married/cohabiting was 45.3% (range = 0–99.6%), while that of FSWs reporting non-paying partners was 48.5% (range = 6.8–86.2%). The mean number of non-paying partners in the past month ranged between 1 and 3, with about two thirds reporting only one partner.

Only few studies investigated group sex: 7.7% [90] of FSWs reported ever engaging in group sex, 6.2% [68] and 12.9% [68] reported group sex in the past month, and 10.0% [58] in the past week.

#### Injecting risk behavior, sex with PWID, and substance use

There was a large variability in injecting risk behavior and substance use among FSWs, but the highest levels of injecting drug use were reported in Iran and Pakistan (Additional file 1: Table S12). Median of *current*/*recent* injecting drug use was 2.1% (range = 0–26.6%), but the majority of studies were from Pakistan. Studies in Iran reported a *history* of injecting drug use in the range of 6.1–18.0% (median of 13.6%) among all FSWs and range of 16.4–25.5% (median of 22.3%) among only ever/active drug users. A history of injecting drug use was reported by < 1% (median) of all FSWs (range = 0%–11.8%) in the rest of MENA countries.

Fraction of FSWs reporting current/recent sex with PWID ranged from 0.5 to 13.6% within Afghanistan and 0–54.9% within Pakistan, with medians of 5.2% and 5.6%, respectively. Sex with PWID was reported at 23.6% [93] among FSWs in Iran.

Close to a third of FSWs reported ever using drugs (median = 27.0%, range = 1.7–90.7%). A median of 8.9% reported current/recent drug use (range = 0.6–59.0%). Any substance use before/during sex was reported by 37.8% (median, range = 1.0–88.1%). Alcohol use before/during sex was reported by 44.1% (median, range = 3.0–70.7%).

#### Knowledge of HIV/AIDS and perception of risk

Knowledge of HIV/AIDS was generally high among FSWs across MENA (Additional file 1: Table S13). Vast majority of FSWs ever heard of HIV (median = 81.9%, range = 25.4–100%) and were aware of sexual (median = 72.0%, range = 50.8–94.9%) and injecting (median = 88.7%, range = 11.5–99.6%) modes of transmission, but to a lesser extent of condoms as a prevention method (median = 51.6%, range = 14.1–89.8%)—condoms were more perceived as a contraception method. Levels of knowledge, however, varied often substantially within the same country.

Overall, FSWs did not perceive themselves at high risk of HIV acquisition (Additional file 1: Table S14). Perception of HIV risk was reported as at-risk (median = 34.6%, range = 22.8–48.5), low-risk (median = 18.3%, range = 7.1–46.9), medium-risk (median = 16.4%, range = 5.3–36.1), and high-risk (median = 14.4%, range = 5.9–32.0).

#### HIV testing

HIV testing among FSWs varied across countries, but was generally low, with a median fraction of 17.6% (range = 4.0–99.4%) ever tested for HIV (Additional file 1: Table S15). Only a median of 12.1% (range = 0.9–38.0%) of all FSWs tested for HIV in the past 12 months, and nearly two thirds of those who ever tested did so in the past 12 months (median = 59.2%, range = 33.3–82.0%). Majority of FSWs who ever tested were aware of their status (median = 91.9%, range = 60.0–99.0%).

## Discussion

Through an extensive, systematic, and comprehensive assessment of HIV epidemiology among FSWs and clients, including data presented in the scientific literature for the first time, we found that HIV epidemics among FSWs have already emerged in MENA, and some appear to have reached their peak. Based on a synthesis and triangulation of evidence from studies on a total of 300,000 FSWs and 30,000 clients, a strong regionalization of epidemics has been identified. In Djibouti and South Sudan, the HIV epidemic is concentrated with a prevalence of ~ 20% in FSWs. In Algeria, Libya, Morocco, Somalia, and Sudan, the epidemic is of intermediate-intensity (prevalence 1–5%). Strikingly, in the remaining countries with available data, the prevalence is < 1%, and most often zero.

A key finding is that HIV prevalence in FSWs has been (overall) growing steadily since 2003. This is the same time in which independent evidence has identified the emergence of major epidemics among both PWID [11] and MSM [10] in MENA. It is probable that the epidemics among these key populations have been bridged to FSWs. An example is Pakistan, where the prevalence among FSWs was < 1% in almost all cities in three consecutive IBBSS rounds between 2005 and 2012 [38, 40, 69]. However, prevalence ranging from 1.5 to 8.8% was documented in half of the cities in the latest round in 2016–2017 [42]. These emerging epidemics among FSWs were preceded by large and growing epidemics first among PWID [11] and then among MSM [10, 11].

Some of the FSW epidemics, particularly those in Djibouti and South Sudan, emerged much earlier, most likely by late 1980s [6], mainly affected by geographic proximity and stronger population links to sub-Saharan Africa (SSA) [6]. Djibouti is a port country and the major trade route for Ethiopia and a station for large international military bases [6, 151]. The majority of FSWs operating in Djibouti are Ethiopians catering to the Ethiopian truck drivers transporting shipments from the Djibouti port [84–86]. South Sudan is socio-culturally part of SSA, with a major fraction of FSWs coming from Uganda, Congo, and Kenya [79]. In these MENA countries, HIV in commercial heterosexual sex networks (CHSNs) is well-established and epidemics are concentrated—though at levels lower than the hyper-endemic epidemics observed in SSA [152].

Unlike the epidemics among PWID and MSM [10, 11], the FSW epidemics have been overall growing rather slowly, with the prevalence being mostly < 5%. Strikingly, a considerable fraction of countries still do not appear to have much HIV transmission in CHSNs, with consistently very low prevalence, quite often even at zero level—46.8% of studies in FSWs reported zero prevalence, and 7 out of 18 countries had a pooled mean prevalence of zero or nearly zero. One explanation for the observed low HIV prevalence could be that HIV has not yet been effectively introduced into CHSNs—it took decades for HIV to be effectively introduced into PWID [11] and MSM [10] networks. Another possible factor pertains to the structure of CHSNs, characterized apparently by low connectivity [6, 153, 154], which reduces the risk of HIV being introduced, or efficiently/sustainably transmitted. Unlike PWID and MSM, FSWs are also exposed to HIV mainly through their clients, who have a lower risk of exposure to HIV than themselves, thus possibly contributing to slower epidemic growth [6].

Other factors may also contribute to explaining the observed low HIV prevalence. The synthesized evidence suggests a lower risk environment for FSWs in MENA, compared to other regions. The reported number of clients is rather low at a median of 34 per month, at the lower end of global range [155–158]. Close to half of commercial sex acts are protected through condom use, with no difference between regular and one-time clients, despite noted variability across and within countries. HIV/AIDS knowledge also varies, but is generally substantial, with the majority of FSWs being aware of sexual and injecting modes of transmission, and over half are aware of condoms as a prevention method. Injecting drug use and sex with PWID is low in most countries, except for countries in Eastern MENA, notably Afghanistan, Iran, and Pakistan. Serological markers for hepatitis C virus (a marker of injecting risk) [159–161] are also low in FSWs, assessed at a median of 1.1% (range = 0–9.9%, not shown), with the highest measures reported in Iran [61, 162]. These relatively lower levels of risk behavior than other regions [163–165] stand in contrast to what has been observed in PWID and MSM in MENA [10, 11].

Importantly, with the efficacy of 60% in randomized clinical trials [166–169], male circumcision, which is essentially at universal coverage across MENA [170], may have also slowed, or even substantially reduced HIV transmission in CHSNs leading to the observed low HIV prevalence [171]. Incidentally, the two most affected countries—South Sudan and Djibouti—are nearly the only two major settings where male circumcision is at low coverage in MENA, either nationally, as is the case for South Sudan [170], or among clients of FSWs, as is the case for Ethiopian truckers and international military personnel stationed in Djibouti [151, 170]. Though HIV prevalence will probably continue to increase among FSWs and clients, the high levels of male circumcision coupled with lower levels of risk behavior may prevent significant epidemics, as seen elsewhere [172–174], from materializing in CHSNs in multiple MENA countries.

HIV prevalence in FSWs in few countries, particularly in Eastern MENA, may not necessarily reflect heterosexual as much as iatrogenic exposures through injecting drug use. Specifically, in Iran and Pakistan, countries with large HIV epidemics among PWID [11], a considerable fraction of FSWs report current/recent/history (14% in Iran and 2% in Pakistan) of injecting drug use. High prevalence of sex work is also reported in women engaging in injecting drug use [93, 175, 176]. Current/recent/history of sex with PWID is also common (24% in Iran and 6% in Pakistan). The overlap between these key populations suggests a potential for HIV to be bridged from PWID networks to CHSNs, as seem to have occurred in Pakistan recently [42, 177, 178].

Population proportion of current/recent FSWs ranged from 0.2 to 2.4% across studies with a median of 0.6%, while that of current/recent clients ranged from 0.3 to 13.8% with a median of 5.7%, both on the lower end of global range [179, 180]. Though these population proportions may seem small, the size of CHSNs is much larger than that of PWID and MSM [10, 11, 181]. This suggests that CHSNs could be a main driver of HIV incidence in many countries despite the low HIV prevalence in FSWs. An example is Morocco where the mode of transmission analyses estimated that over half of HIV incidence is driven by CHSNs, despite an HIV prevalence of only ~ 2% in FSWs [182–184]. The role of CHSNs is even more significant in countries with concentrated epidemics. In Djibouti, for example, the large HIV epidemic among FSWs was mirrored shortly after by a rapid rise in prevalence among clients (as proxied by male STI clinic attendees; Table 4), leading eventually to a prevalence > 1% in pregnant women [6].

HIV response to the epidemic in CHSNs in MENA continues to be weak and limited in scope and scale [185]. Criminality [151, 185] and stigma [186–188] associated with sex work persist as barriers to surveillance and targeted programming [189–191], leading even to the resistance to acknowledge the existence of sex work [192]. These challenges are compounded by the diverse typologies and increased mobility of FSWs [41, 70, 151]. Across MENA, only 18% of FSWs reported ever testing for HIV, and fewer (12%) reported testing in the past 12 months, far below the 90% service coverage target of “UNAIDS 2016–2021 Strategy” [193]. Programs, including healthcare provision, where they exist, are nearly always implemented by non-governmental organizations (NGOs), who often lack the resources or legal coverage to deliver comprehensive prevention interventions [6, 185].

There are, however, notable exceptions. Morocco has established an evidence-informed national strategy and rapidly scaled up provision of comprehensive services for at-risk populations, including outreach peer education programs as well as testing and case management services [183, 185]. Voluntary counseling and testing centers were established nationwide, with FSWs estimated to constitute about a quarter of attendees in 2007 [183, 194]. Findings of the 2011–2012 IBBSS indicated that over a third of FSWs ever tested for HIV, the vast majority of whom were aware of their status [67]. Condom use at last sex also increased from 37% in 2003 to a median of 50% in 2011 (Additional file 1: Table S11). Morocco’s success has been grounded on a strong multisectorial response where NGOs, in partnership with the government, play a leading role in implementing interventions [185]. In Iran, the large expansion of harm reduction services, including the first women-operated services in MENA [11], is a promising step for targeting FSWs most at risk.

This study is limited by gaps in evidence. Epidemic status among FSWs remains unknown in six countries, as no data were identified. Others (Bahrain and Libya) also had limited data to warrant a meaningful characterization of the epidemic. The high heterogeneity of epidemics within countries suggests that caution is needed when interpreting data without a representative national coverage. For instance, while concentrated epidemics among FSWs are documented in southern Morocco [67, 195] and southern Algeria [113, 196–198], these do not appear to be representative of FSWs at the national level [42, 67, 74, 78, 81, 82, 113, 195–199]. Hidden epidemics or outbreaks may also exist in specific geographies within the country, but not necessarily elsewhere. Data varied over time with high quality and volume of evidence available mostly post-2000, thanks to the expansion and funding of IBBSS studies. While the pooled prevalence estimates were meant to provide a summary of the relative standing of MENA countries in the HIV epidemic, the large between-study heterogeneity suggests that caution is warranted when interpreting these estimates. Studies in clients of FSWs/proxy populations remain limited with wide variability in evidence availability across MENA.

A considerable fraction of studies used convenience sampling, although meta-regression indicated no difference in the prevalence by sampling methodology. This may be explained by FSWs being more “visible” [151, 200] compared to PWID [11] and MSM [10]. A sizable fraction of studies was from routine data reporting with no sufficient documentation of study methodology. However, most of these country-level program data were presumably based on rigorous case definitions following WHO guidelines [6]. There is also a possibility that a fraction of studies may have enrolled women without a strict and valid definition for sex work, yet meta-regression findings showed no effect for the validity of sex work definition on HIV prevalence. There was also no evidence that other study-specific quality domains, including HIV ascertainment method and response rate, had an effect on prevalence. A considerable fraction of studies reported zero prevalence, thus an increment of 0.1 was added to a number of events to be able to conduct the meta-regressions. While this choice of increment was arbitrary, other increments yielded the same findings, though some of the effect sizes changed in scale. There was evidence for a small-study effect in meta-regression suggesting potential publication bias towards studies reporting higher prevalence.

## Conclusions

HIV epidemics among FSWs are emerging in MENA, with some already established. The epidemic has been growing steadily in recent years, with strong regionalization and heterogeneity. A contributing factor to epidemic growth appears to be the epidemics that emerged among PWID [11] and MSM [10] nearly two decades ago. Strikingly, a large fraction of countries still do not appear to have any significant epidemic dynamics in CHSNs. These findings demonstrate the need for expanding surveillance systems, including the conduct of repeated IBBSS studies with national coverage to monitor HIV prevalence trends and to detect the emergence of epidemics. There is also a pressing need for mapping and size estimation studies to delineate the diverse typologies of sex work and to ensure evidence-informed response with adequate coverage of interventions.

Achieving “UNAIDS 2016–2021 Strategy” [193] service coverage targets entails reaching out to the increasingly dispersed FSW population [41, 70, 151]. Building on Morocco’s success, this would be best achieved through NGOs leading the provision of comprehensive interventions, with governmental support, even if discrete. Extending harm reduction services to women PWID is also critical to curb HIV burden in FSWs most at risk, specifically in Eastern MENA. The window of opportunity for detecting epidemics at their nascence, and for controlling incidence in CHSNs, should not be missed.

## Additional file

Additional file 1: Supplementary information including further details and additional results for the systematic review and meta-analytics of HIV infection in female sex and their clients workers in the Middle East and North Africa. **Tables S1-S15.** **Figure S1.** **Box S1-S2.** (DOCX 1819 kb)

## Data Availability

All data are within the paper and its supplementary information.

## Abbreviations

AOR: Adjusted odds ratio
CHSNs: Commercial heterosexual sex networks
CI: Confidence interval
FSWs: Female sex workers
IBBSS: Integrated bio-behavioral surveillance surveys
MENA: Middle East and North Africa
MSM: Men who have sex with men
NGOs: Non-governmental organizations
PRISMA: Preferred Reporting Items for Systematic Reviews and Meta-analyses
PWID: People who inject drugs
ROB: Risk of bias
SI: Supplementary Information
SSA: Sub-Saharan Africa
STI: Sexually transmitted infection
UNAIDS: Joint United Nations Programme on HIV/AIDS
WHO: World Health Organization
