# Supplementary material for: HIV epidemiology among female sex workers and their clients in the Middle East and North Africa: systematic review, meta-analyses, and meta-regressions
Source: BMC Med. 2019 Jun 24;17:119. doi: 10.1186/s12916-019-1349-y (PMC6589882; doi:10.1186/s12916-019-1349-y)
Supplement: Supplementary file 1 — Supplementary information including further details and additional results for the systematic review and meta-analytics of HIV infection in female sex and their clients workers in the Middle East and North Africa. Tables S1-S15. Figure S1. Box S1-S2. (DOCX 1819 kb) [file 12916_2019_1349_MOESM1_ESM.docx]

**Supplementary Information**

**HIV epidemiology among female sex workers and their clients in the Middle East and North Africa: Systematic review, meta-analyses, and meta-regressions**

Hiam Chemaitelly MSc,^*1,3^ Helen A. Weiss PhD,^2,3^ Clara Calvert PhD,^3^ Manale Harfouche MPh,^1^ and Laith J. Abu-Raddad PhD^1,4,5^

*^1^Infectious Disease Epidemiology Group, Weill Cornell Medicine-Qatar, Cornell University, Qatar Foundation – Education City, Doha, Qatar*

*^2^MRC Tropical Epidemiology Group, London School of Hygiene and Tropical Medicine, London, United Kingdom*

*^3^Department of Infectious Disease Epidemiology, Faculty of Epidemiology and Population Health, London School of Hygiene and Tropical Medicine, London, United Kingdom*

*^4^Department of Healthcare Policy & Research, Weill Cornell Medicine, Cornell University, New York, New York, USA*

^5^College of Health and Life Sciences, Hamad bin Khalifa University, Doha, Qatar

**^*^Reprints or correspondence**

Hiam Chemaitelly, Weill Cornell Medicine-Qatar, Qatar Foundation - Education City, P.O. Box 24144, Doha, Qatar. Telephone: +(974) 4492-8443. Fax: +(974) 4492-8422. E-mail: [hsc2001@qatar-med.cornell.edu](mailto:hsc2001@qatar-med.cornell.edu)

**Table of Contents**

Table S1 Preferred Reporting Items for Systematic Reviews and Meta-analyses (PRISMA) checklist3

Fig. S1 Map of the Middle East and North Africa region5

**Box S1** Search criteria for the systematic review of size estimation, HIV incidence, and HIV prevalence studies in FSWs and their clients, in the Middle East and North Africa6

**Box S2** List of extracted variables for the systematic review of HIV epidemiology among FSWs and their clients in the Middle East and North Africa9

**Table S2** Quality assessment criteria for size estimation and HIV prevalence studies in FSWs and their clients (or proxy populations of clients) in the Middle East and North Africa, as identified in the systematic review10

**Table S3** Details of variables and subcategories included in the meta-regression analyses11

**Table S4** Estimates of subnational representation for the number and population proportion of FSWs and of their clients in the Middle East and North Africa reported by identified studies12

**Table S5** HIV point-prevalence measures in FSWs as extracted or obtained from various sources including the US Census Bureau database, the WHO-EMRO, and the UNAIDS epidemiological fact sheets databases, among other sources of data22

**Table S6** Summary of the risk of bias assessment of size estimation and HIV prevalence studies in FSWs and their clients (or proxy populations of clients), in the Middle East and North Africa 31

**Table S7** Risk of bias assessment of estimates of national and subnational representation for the number and population proportion of FSWs and of their clients, in the Middle East and North Africa32

**Table S8** Risk of bias assessment of HIV prevalence studies in FSWs in the Middle East and North Africa37

**Table S9** Risk of bias assessment of HIV prevalence studies in clients of FSWs (or proxy populations of clients) in the Middle East and North Africa40

**Table S10** Results of meta-regression analyses to identify associations with HIV prevalence, sources of between-study heterogeneity, and trend in HIV prevalence in clients of FSWs (or proxy populations of clients such as male STI clinic attendees), in the Middle East and North Africa41

**Table S11** Condom use among FSWs and their clients in the Middle East and North Africa42

**Table S12** Measures of injecting drug use and overlap with people who inject drugs among FSWs in the Middle East and North Africa49

**Table S13** HIV/AIDS knowledge among FSWs in the Middle East and North Africa53

**Table S14** Perception of risk among FSWs in the Middle East and North Africa54

**Table S15** HIV testing among FSWs in the Middle East and North Africa55

**References**57

**Table S1** Preferred Reporting Items for Systematic Reviews and Meta-analyses (PRISMA) checklist [1]

| Section/topic | # | Checklist item | Reported in main text |
| --- | --- | --- | --- |
|  | | | |
| Title | 1 | Identify the report as a systematic review, meta-analysis, or both. | p. 1 |
|  | | | |
| Structured summary | 2 | Provide a structured summary including, as applicable: background; objectives; data sources; study eligibility criteria, participants, and interventions; study appraisal and synthesis methods; results; limitations; conclusions and implications of key findings; systematic review registration number. | p. 2-3 |
|  | | | |
| Rationale | 3 | Describe the rationale for the review in the context of what is already known. | p. 4 |
| Objectives | 4 | Provide an explicit statement of questions being addressed with reference to participants, interventions, comparisons, outcomes, and study design (PICOS). | p. 4-5 |
|  | | | |
| Protocol and registration | 5 | Indicate if a review protocol exists, if and where it can be accessed (e.g., Web address), and, if available, provide registration information including registration number. | NA |
| Eligibility criteria | 6 | Specify study characteristics (e.g., PICOS, length of follow-up) and report characteristics (e.g., years considered, language, publication status) used as criteria for eligibility, giving rationale. | p. 5-6 |
| Information sources | 7 | Describe all information sources (e.g., databases with dates of coverage, contact with study authors to identify additional studies) in the search and date last searched. | p. 5 & Box S1 in SI |
| Search | 8 | Present full electronic search strategy for at least one database, including any limits used, such that it could be repeated. | Box S1 in SI |
| Study selection | 9 | State the process for selecting studies (i.e., screening, eligibility, included in systematic review, and, if applicable, included in the meta-analysis). | p. 5-6 |
| Data collection process | 10 | Describe method of data extraction from reports (e.g., piloted forms, independently, in duplicate) and any processes for obtaining and confirming data from investigators. | p. 6-7 |
| Data items | 11 | List and define all variables for which data were sought (e.g., PICOS, funding sources) and any assumptions and simplifications made. | p. 6-7 & Box S2 in SI |
| Risk of bias in individual studies | 12 | Describe methods used for assessing risk of bias of individual studies (including specification of whether this was done at the study or outcome level), and how this information is to be used in any data synthesis. | p. 7-8 & Table S2 in SI |
| Summary measures | 13 | State the principal summary measures (e.g., risk ratio, difference in means). | p. 8 |
| Synthesis of results | 14 | Describe the methods of handling data and combining results of studies, if done, including measures of consistency (e.g., I^2^) for each meta-analysis. | p.6-8 & Table 5 |
| Risk of bias across studies | 15 | Specify any assessment of risk of bias that may affect the cumulative evidence (e.g., publication bias, selective reporting within studies). | p. 7-8 & Table S2 in SI |
| Additional analyses | 16 | Describe methods of additional analyses (e.g., sensitivity or subgroup analyses, meta-regression), if done, indicating which were pre-specified. | p. 8-9 & S3 Table in SI |
|  | | | |
| Study selection | 17 | Give numbers of studies screened, assessed for eligibility, and included in the review, with reasons for exclusions at each stage, ideally with a flow diagram. | p. 9-10 & Fig. 1 |
| Study characteristics | 18 | For each study, present characteristics for which data were extracted (e.g., study size, PICOS, follow-up period) and provide the citations. | p.10-11, Tables 1-4, and Tables S4 & S5in SI |
| Risk of bias within studies | 19 | Present data on risk of bias of each study and, if available, any outcome-level assessment (see Item 12). | p. 12 & Tables S6-S9 in SI |
| Results of individual studies | 20 | For all outcomes considered (benefits or harms), present, for each study: (a) simple summary data for each intervention group and (b) effect estimates and confidence intervals, ideally with a forest plot. | p. 10-11, Tables 1-4 & Tables S4-S5 in SI |
| Synthesis of results | 21 | Present results of each meta-analysis done, including confidence intervals and measures of consistency. | p.12-13 & Table 5 |
| Risk of bias across studies | 22 | Present results of any assessment of risk of bias across studies (see Item 15). | p. 12 & Tables S6-S9 in SI |
| Additional analysis | 23 | Give results of additional analyses, if done (e.g., sensitivity or subgroup analyses, meta-regression [see Item 16]). | p. 13-17, Table 6, & Tables S10-S15 in SI |
| DISCUSSION | | | |
| Summary of evidence | 24 | Summarize the main findings including the strength of evidence for each main outcome; consider their relevance to key groups (e.g., health care providers, users, and policy makers). | p. 18-22 |
| Limitations | 25 | Discuss limitations at study and outcome level (e.g., risk of bias), and at review level (e.g., incomplete retrieval of identified research, reporting bias). | p. 22-23 |
| Conclusions | 26 | Provide a general interpretation of the results in the context of other evidence, and implications for future research. | p. 23-24 |
|  | | | |
| Funding | 27 | Describe sources of funding for the systematic review and other support (e.g., supply of data); role of funders for the systematic review. | p. 26 |

*Abbreviations*: *NA* not applicable, *P* page(s), *SI* Supporting information

**Fig. S1** Map of the Middle East and North Africa region. The definition for this region covers 23 countries including Afghanistan, Algeria, Bahrain, Djibouti, Egypt, Iran, Iraq, Jordan, Kuwait, Lebanon, Libya, Morocco, Oman, Pakistan, Palestine, Qatar, Saudi Arabia, Somalia, Sudan (available studies for Sudan before 2011, the year of independence of South Sudan, may have come from both Sudan and the newly indpendent Republic of South Sudan), Syria, Tunisia, United Arab Emirates (UAE), and Yemen. This definition is based on definitions of the World Health Organization, the Joint United Nations Programme on HIV/AIDS, and the World Bank [2]

**
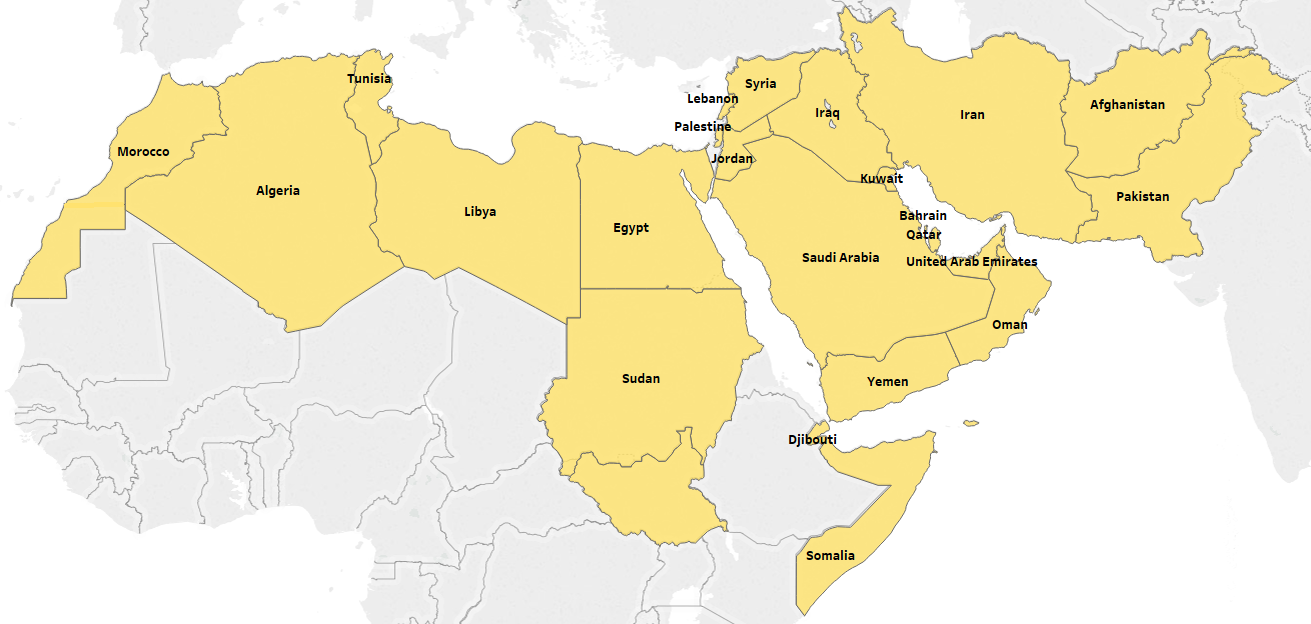
**

**Box S1** Search criteria for the systematic review of size estimation, HIV incidence, and HIV prevalence studies in FSWs and their clients, in the Middle East and North Africa (MENA)

| **PubMed** (July 29, 2018) |
| --- |
| ***Sex work*** |
| "Extramarital Relations"[Mesh] OR “Sex Work*”[Mesh] OR "Sex/analysis"[Mesh] OR "Sex/statistics and numerical data"[Mesh] OR "Sexual partners"[Mesh] OR "Sex Trafficking/epidemiology"[Mesh] OR "Sex Trafficking/statistics and numerical data"[Mesh] OR Sex work*[Text] OR Sexual work*[Text] OR Sexwork*[Text] OR Sex-work*[Text] OR Sexual partner*[Text] OR Sex partner*[Text] OR Sexual contact*[Text] OR FSW[Text] OR FSWs[Text] OR CSW[Text] OR CSWs[Text] OR SW[Text] OR SWs[Text] OR TSW[Text] OR TSWs[Text] OR TS[Text] OR Travailleuse* sexe[Text] OR Travailleuse* sex[Text] OR Bar girl*[Text] OR Callgirl*[Text] OR Call girl*[Text] OR Escort*[Text] OR Masseuse*[Text] OR Hostess*[Text] OR ((Premarital[Text] OR Pre-marital[Text] OR Pre marital[Text] OR Extramarital[Text] OR Extra-marital[Text] OR Extra marital[Text] OR Illicit[Text] OR Illegal[Text]) AND (Sex[Text] OR Sexual[Text] OR Relation*[Text])) OR Outside marriage[Text] OR Out of marriage[Text] OR “Illegal social behavior”[Text] OR “Illegal social behaviour”[Text] OR Adultery[Text] OR Prostitut*[Text] OR Promiscu*[Text] OR Female entertain*[Text] OR Sex entertain*[Text] OR Sexual* entertain*[Text] OR Entertainment work*[Text] OR Sex industr*[Text] OR Sex establishment*[Text] OR Brothel*[Text] OR Red light[Text] OR Red-light[Text] OR Red district*[Text] OR Nightclub*[Text] OR Pimp[Text] OR ((Intergenerational[Text] OR Cross-generation*[Text] OR Cross-generational[Text] OR Recreational[Text] OR Commercial[Text] OR Transaction*[Text] OR Casual[Text] OR Group[Text] OR Informal[Text] OR Street[Text] OR Migrant*[Text] OR Survival[Text] OR Occupational[Text] OR Tourism[Text]) AND (Sex[Text] OR Sexual*[Text])) OR Sex seeking[Text] OR Sex-seeking[Text] OR Solicit*[Text] OR ((Provision*[Text] OR Provider*[Text] OR Provid*[Text] OR Sell*[Text] OR Sold[Text] OR Exchang*[Text] OR Trad*[Text] OR Favor*[Text] OR Consum*[Text] OR Commodi*[Text] OR Paid[Text] OR Paying[Text] OR Pay[Text] OR Payer*[Text] OR Buying[Text] OR Buy[Text] OR Buyer*[Text] OR Charg*[Text] OR Engag*[Text] OR Service*[Text] OR Money[Text] OR Cash[Text] OR Drug*[Text] OR Goods[Text] OR Gift*[Text]) AND (Sex[Text] OR Sexual*[Text])) OR Hidden population*[Text] OR Hard to reach population*[Text] OR Hard-to-reach population*[Text] OR Core group*[Text] OR Core risk group*[Text] OR Vulnerable women[Text] OR Vulnerable population*[Text] OR Vulnerable female*[Text] OR Most-at-risk population*[Text] OR Most at risk population*[Text] OR High risk population*[Text] OR High-risk population*[Text] OR Population* at high risk[Text] OR Population* at high-risk[Text] OR ((Traffick*[Text] OR Slave*[Text] OR Coerc*[Text] OR Abduct*[Text] OR Exploit*[Text] OR Abuse*[Text] OR Violence[Text]) AND (Sex[Text] OR Sexual*[Text])) |
| ***MENA*** |
| "Middle East"[Mesh] OR "Islam"[Mesh] OR "Arabs"[Mesh] OR "Arab World"[Mesh] OR "Africa, Northern"[Mesh] OR "Sudan"[Mesh] OR "Somalia"[Mesh] OR "Djibouti"[Mesh] OR "Pakistan"[Mesh] OR “South Sudan”[Mesh] OR "Middle East*"[Text] OR "Middle-East"[Text] OR "North Africa*"[Text] OR "North-Africa"[Text] OR "EMRO"[Text] OR "Eastern Mediterranean"[Text] OR “Arab*”[Text] OR “Arab World”[Text] OR "Islam*"[Text] OR "Afghanistan"[Text] OR "Afghan*"[Text] OR "Algeria*"[Text] OR "Bahrain*"[Text] OR "Djibouti"[Text] OR "Egypt*"[Text] OR "Jordan*"[Text] OR "Kuwait*"[Text] OR "Lebanon"[Text] OR "Leban*"[Text] OR "Libya*"[Text] OR "Iran*"[Text] OR "Iraq*"[Text] OR "Morocco"[Text] OR “Moroccan*”[Text] OR "Oman*"[Text] OR "Pakistan*"[Text] OR "Qatar*"[Text] OR "Saudi*"[Text] OR "Somalia"[Text] OR "Somal*"[Text] OR "Sudan*"[Text] OR "Syria*"[Text] OR "Tunisia*"[Text] OR "United Arab Emirates"[Text] OR "Emirat*"[Text] OR "West Bank"[Text] OR "Ghaza*"[Text] OR "Gaza*"[Text] OR "Palestine"[Text] OR "Palestinian*"[Text] OR "Yemen*"[Text] OR “UAE”[Text] OR “KSA”[Text] |
| ***Women*** |
| "Female/analysis"[Mesh] OR "Female/statistics and numerical data"[Mesh] OR “Women/epidemiology”[Mesh] OR “Women/statistics and numerical data”[Mesh] OR Women[Text] OR Girl*[Text] OR Female*[Text] |
| ***Clients/Men*** |
| “Male/complications"[Mesh] OR "Male/diagnosis"[Mesh] OR "Men/statistics and numerical data"[Mesh] OR Men[Text] OR Male[Text] OR Males[Text] OR Client*[Text] OR Paying partner*[Text] OR Sugar daddy[Text] OR Sugar daddies[Text] |
|  |
| **FINAL PUBMED SEARCH** |
| **(“Sex work” AND “MENA” AND “Women”) OR (“Sex work” AND “MENA” AND “Clients/Men”)** |
|  |
| **Embase** (July 29, 2018) |
| ***Sex work*** |
| exp prostitution/ or exp casual sex/ or exp transactional sex/ or exp group sex/ or exp sex tourism/ or exp sexual promiscuity/ or exp extramarital sex/ or exp premarital sex/ or exp sexual relation/ or exp sexual partners/ or ((exp sex trafficking/ or exp sexual exploitation/ or exp sexual coercion/) NOT Child) or (sex* work* or sexwork* or sex-work* or sex partner* or sexual partner* or sexual contact* or premarital sex or premarital sexual or premarital relation* or pre-marital sex or pre-marital sexual or pre-marital relation* or pre marital sex or pre marital sexual or pre marital relation* or extramarital sex or extramarital sexual or extramarital relation* or extra-marital sex or extra-marital sexual or extra-marital relation* or extra marital sex or extra marital sexual or extra marital relation* or illicit sex or illicit sexual or illicit relation* or illegal sex or illegal sexual or illegal relation* or (out* ADJ1 marriage) or illegal social behavio?r or adultery or prostitut* or promiscu* or FSW or FSWs or CSW or CSWs or SW or SWs or TSW or TSWs or TS or (women ADJ4 sex*) or (Travailleuse* ADJ1 sex*) or bar girl* or call girl* or callgirl* or escort* or masseuse* or hostess* or female entertain* or sex entertain* or sexual entertain* or entertainment work* or sex industr* or sex establishment* or brothel* or red light or red-light or (red ADJ1 district*) or nightclub* or pimp or recreation* sex* or intergenerational sex* or cross-generation sex* or cross-generational sex* or commercial sex* or transactional sex* or sex* transaction* or casual sex* or informal sex* or group sex* or street sex* or (migra* ADJ4 sex*) or (sex* ADJ4 migra*) or survival sex* or occupational sex* or sex* tourism or sex seeking or sex-seeking or solicit* or (consum* ADJ4 sex*) or (sex* ADJ 4 consumer) or (sex* ADJ4 consumers) or (sex* ADJ4 provi*) or (provi* ADJ4 sex*) or (sell* ADJ4 sex*) or (sex* ADJ4 sell*) or sold sex* or (exchang* ADJ4 sex*) or (sex* ADJ4 exchange) or (trading ADJ4 sex*) or (trade* ADJ4 sex*) or sex* trade or sex* favor* or (commodi* ADJ4 sex*) or (sex* ADJ4 commodi*) or (paid ADJ4 sex*) or (pay* ADJ4 sex*) or (sex* ADJ4 pay*) or (buy* ADJ4 sex*) or (sex* ADJ4 buy*) or (charg* ADJ4 sex*) or (sex* ADJ4 charg*) or (engag* ADJ4 sex*) or (sex* ADJ4 engage*) or (sex* ADJ4 service*) or (service* ADJ4 sex*) or (money ADJ4 sex*) or (sex* ADJ4 money) or (cash ADJ4 sex*) or (sex* ADJ4 cash) or (sex* ADJ4 drug*) or (drug* ADJ4 sex*) or (sex* ADJ4 goods) or (goods ADJ4 sex*) or (sex* ADJ4 gift*) or (gift* ADJ4 sex*) or hidden population* or hard to reach population* or hard-to-reach population* or (core ADJ1 group*) or vulnerable women or vulnerable female*).mp. or ((vulnerable population* or most-at-risk population* or most at risk population* or high risk population* or high-risk population* or population* at high risk or population* at high-risk).mp. AND (sex* or infection* or STI or STIs or STD or STDs or human immunodeficiency virus or HIV* or AIDS* or acquired immune deficiency syndrome or acquired immunodeficiency syndrome).mp.) or ((sex trafficking or sexual trafficking or (traffick* ADJ4 sex*) or sex* slave* or sex* coerc* or sex* abduct* or sex* exploit* or sex* abuse* or sex* violence) NOT Child).mp. or ((women ADJ4 traffick*) or (girls ADJ4 traffick*) or (female* ADJ4 traffick*) or (traffick* ADJ4 women) or (traffick* ADJ4 girls) or (traffick* ADJ4 female*)).mp. |
| ***MENA*** |
| exp Middle East/ or exp North Africa/ or exp Arab/ or exp Afghanistan/ or exp Djibouti/ or exp Pakistan/ or exp Somalia/ or exp Sudan/ or exp South Sudan/ or Middle East.mp. or North Africa.mp. or EMRO.mp. or Eastern Mediterranean.mp. or Arab.mp. or Arabs.mp. or Arab World.mp. or Islam.mp. or Afghanistan.mp. or Afghan*.mp. or Algeria*.mp. or Bahrain*.mp. or Djibouti.mp. or Egypt*.mp. or Jordan*.mp. or Kuwait*.mp. or Leban*.mp. or Libya*.mp. or Iran*.mp. or Iraq*.mp. or Morocc*.mp. or Oman*.mp. or Pakistan*.mp. or Qatar*.mp. or Saudi*.mp. or Somal*.mp. or Sudan*.mp. or Syria*.mp. or Tunisia*.mp. or United Arab Emirates.mp. or Emirat*.mp. or West Bank.mp. or Ghaza*.mp. or Gaza*.mp. or Palestin*.mp. or Yemen*.mp. or UAE.mp. or KSA.mp. |
| ***Women*** |
| exp female/ or (women or girl* or female*).mp. |
| ***Clients/Men*** |
| exp male/ or (client* or (paying ADJ1 partner*) or sugar dadd* or men or male*).mp. |
|  |
| **FINAL EMBASE SEARCH** |
| **(“Sex work” AND “MENA” AND “Women”) OR (“Sex work” AND “MENA” AND “Clients/Men”)** |
|  |
| **Regional databases** |
| **HIV and AIDS Asia Pacific Research Statistical Data Information** (May 27, 2018) |
| Keyword search for: “Afghanistan” and “Pakistan” |
|  |
| **Iran Scientific Information Database** (July 23, 2018**)** |
| Keyword search for: “HIV”, “AIDS”, “Human immunodeficiency virus”, “Acquired immune deficiency syndrome”, “sex work”, “prostitute”, “size estim”, and “sexually transmitted infection” |
|  |
| **Iraq Academic Scientific Journals database** (July 23, 2018) |
| Keyword search for: “HIV OR AIDS”, “HIV”, “Human immunodeficiency virus”, “Acquired immune deficiency syndrome”, “sex work”, “prostitute”, “commercial sex”, “size estimation”, and “sexually transmitted infection” |
|  |
| **MENA HIV/AIDS Epidemiology Synthesis Project database** (June 01, 2018) |
| Hand search of all documents in the database |
|  |
| **PakMediNet** (July 23, 2018) |
| Keyword search for: “HIV”, “AIDS”, “Human immunodeficiency virus”, “Acquired immune deficiency syndrome”, “sex work”, “prostitute”, “commercial”, “size estimation”, and “sexually transmitted infection” |
|  |
| **US Census Bureau** (July 17, 2018) |
| Keyword search using each MENA country name |
|  |
| **World Health Organization Index Medicus for the Eastern Mediterranean Region** (July 23, 2018) |
| Keyword search for: “HIV OR AIDS”, “Human AND immunodeficiency AND virus”, “Acquired AND immune AND deficiency AND syndrome”, “prostitute”, and “sex AND worker” |
| **World Health Organization Index Medicus for the Eastern Mediterranean Region** (July 27, 2018) |
| Keyword search for: “Algeria”, “Algerie”, “Djibouti”, “Egypt”, “Egypte”, “Libya”, “Libie”, “Maroc”, “Morocco”, “Tunisia”, “Tunisie”, “Somalia”, “Somalie”, “Sudan”, and “Soudan” |
|  |
| **Abstract archives of the International AIDS Society conferences** (July 28, 2018) |
| Keyword search using each MENA country name |

*Abbreviations*: *FSWs* female sex workers

**Box S2** List of extracted variables for the systematic review of HIV epidemiology among FSWs and their clients in the Middle East and North Africa (MENA)

| **Report characteristics** |
| --- |
| Author(s), year of publication, full citation, type of publication, and source of data |
|  |
| **General study characteristics** |
| Study population and its characteristics, year(s) of data collection, country of origin, country of survey, city, study site, study design, sampling methodology, estimation methodology, sample size, population definition, eligibility criteria, and participation rate |
|  |
| **Studies/outcome measures** |
| Population-size estimates and population proportions of FSWs and clients |
|  |
| HIV incidence (including number followed-up, follw-up time, sero-conversion risk, incidence rate, and details related to outcome ascertainment) |
|  |
| HIV prevalence (including number tested, number antibody positive, and details related to outcome ascertainment) |
|  |
| **Sexual and injecting risk behaviours and contextual measures** |
| Socio-demographic charcateristics and sex work context (age, age at sexual debut, age at sex work intiation, and marital status), |
|  |
| Condom use with clients and partners (over different time frames, types of sexual partnerships-regular/occasional/paying/non-paying, and sexual acts-vaginal/anal) |
|  |
| Types of sexual partnerships (over different time frames) |
|  |
| Injecting risk behaviour (current/recent/history of drug use, injecting drug use, sex with people who inject drugs, and substance use before or during sex) |
|  |
| Knowledge of HIV/AIDS (knowledge of sexual and injecting modes of transmission, and of condom as HIV prevention method) |
|  |
| Perception of risk of exposure to HIV infection |
|  |
| HIV testing (ever, during the past 12 months, received results) |

*Abbreviations*: *FSWs* female sex workers

**Table S2** Quality assessment criteria for size estimation and HIV prevalence studies in FSWs and their clients (or proxy populations of clients) in the Middle East and North Africa, as identified in the systematic review

| **Quality domain** | **ROB assessment** | **Criteria** | **Size estimation** | **HIV prevalence** |
| --- | --- | --- | --- | --- |
| 1. **Validity of sex work definition** | Low ROB | Clear and valid sex work definition/engagement in paid sex clearly established | X | X |
|  | High ROB | Sex work/engagement in paid sex not well-defined/not clearly established |  |  |
|  | Unclear | Sex work definition/information on engagement in paid sex not provided |  |  |
|  |  |  |  |  |
| 1. **Rigor of estimation methodology** | Low ROB | Method likely to yield representative estimate for the number or population proportion of FSWs or clients such as multiplier unique object, time-location geographical mapping, capture-recapture, and network scale-up, among others | X | NA |
|  | High ROB | Method unlikely to yield representative estimate for the number or population proportion of FSWs or clients such as self-report based on convenience sampling |  |  |
|  | Unclear | Information not reported |  |  |
|  |  |  |  |  |
| 1. **Rigor of sampling methodology** | Low ROB | Studies using probability-based sampling | NA | X |
|  | High ROB | Studies using non-probability sampling |  |  |
|  | Unclear | Information not reported |  |  |
|  |  |  |  |  |
| 1. **Response rate** | Low ROB | ≥60% or ≥60% of target sample size reached in studies using RDS or TLS | X | X |
|  | High ROB | <60% or <60% of target sample size reached in studies using RDS or TLS |  |  |
|  | Unclear | Information not reported |  |  |
|  |  |  |  |  |
| 1. **HIV ascertainment** | Low ROB | HIV ascertainment using biological assays | NA | X |
|  | High ROB | HIV ascertainment based on self-report |  |  |
|  | Unclear | Information not reported |  |  |

*Abbreviations*: *FSWs* female sex workers, *NA* not applicable, *RDS* respondent-driven sampling, *ROB* risk of bias assessment, *TLS* time-location sampling

**Table S3** Details of variables and subcategories included in the meta-regression analyses

| **Variable** | **Sub-categories** |
| --- | --- |
| **Country/subregion**^*^ | 1. Eastern MENA: Afghanistan, Iran, and Pakistan |
|  | 1. Fertile Crescent: Egypt, Iraq, Jordan, Lebanon, Syria |
|  | 1. Bahrain, Kuwait, and Yemen |
|  | 1. Horn of Africa: Djibouti, Somalia, and South Sudan |
|  | 1. North Africa: Algeria, Libya, Morocco, Sudan, and Tunisia |
|  |  |
| **FSW population type** | 1. Street-based, venues-based, and other FSWs |
|  | 1. Bar girls |
|  |  |
| **Total sample size of tested FSWs** | 1. <100 participants |
|  | 1. ≥100 participants |
|  |  |
| **Median year of data collection**^**^ | 1. <1993 |
|  | 1. 1993-2002 |
|  | 1. ≥2003 |
|  |  |
| **Sampling methodology**^†^ | 1. Non-probability sampling |
|  | 1. Probability-based sampling |
|  |  |
| **Response rate** | 1. ≥60% |
|  | 1. <60%/unclear |
|  | 1. Not applicable^‡^ |
|  |  |
| **Validity of sex work definition** | 1. Clear & valid definition |
|  | 1. Poorly defined/unclear |
|  | 1. Not applicable^‡^ |
|  |  |
| **HIV ascertainment** | 1. Biological assays |
|  | 1. Self-report/unclear |
|  | 1. Not applicable^‡^ |

^*^Countries were grouped based on geography and similarity in HIV prevalence levels.

^**^Year grouping was driven by independent evidence identifying the emergence of HIV epidemics among both men who have sex with men [3] and people who inject drugs [4] in multiple MENA countries around 2003.

^†^Sampling methodology was not included in the meta-regression analyses of clients of FSWs as too few studies used probability-based sampling (only four).

^‡^Measures extracted only from routine databases with no reports describing the study methodology were not included in the ROB assessment.

*Abbreviations*: *FSWs* female sex workers

**Table S4** Estimates of subnational representation for the number and population proportion of FSWs and of their clients in the Middle East and North Africa (MENA) reported by identified studies

| Country  Author, year [citation] | Year(s) of data collection | City/ province | Estimation methodology | Sample type | Time frame | Reported size estimate | | | |
| --- | --- | --- | --- | --- | --- | --- | --- | --- | --- |
|  |  |  |  |  |  | N | Range | %^*^ | Range^*^ |
| FSWs |  |  |  |  |  |  |  |  |  |
| Afghanistan |  |  |  |  |  |  |  |  |  |
| SAR AIDS HDS, 2008 [5] | 2006-07 | Jalalabad | Enumeration (time-location geographical mapping) | Home & street-based FSWs | Current | 90 | NR | 0·26 | NR |
| SAR AIDS HDS, 2008 [5] | 2006-07 | Kabul | Enumeration (time-location geographical mapping) | Home & street-based FSWs | Current | 898 | NR | 0·19 | NR |
| SAR AIDS HDS, 2008 [5] | 2006-07 | Mazar-i-Sharif | Enumeration (time-location geographical mapping) | Home & street-based FSWs | Current | 172 | NR | 0·28 | NR |
| NACP, 2012 [6] (round II) | 2012 | Herat | Multiplier unique object | FSWs | Past 12 M | 2,134 | NR | NR | NR |
| NACP, 2012 [6] (round II) | 2012 | Kabul | Multiplier unique object | FSWs | Past 12 M | 2,800 | NR | NR | NR |
| Djibouti |  |  |  |  |  |  |  |  |  |
| Trellu-Kane, 2005 [7] | 2005 | Djibouti | Conv sample (self-report) | Gen pop (13-24 years) | Past 12 M | NR | NR | 4 | NR |
| Egypt |  |  |  |  |  |  |  |  |  |
| Jacobsen, 2014 [8] | 2014 | Giza | Enumeration (time-location geographical mapping) | FSWs | Current | 6,092 | 1,407-7,615 | 0·17 | NR |
| Jacobsen, 2014 [8] | 2014 | Alexandria | Enumeration (time-location geographical mapping) | FSWs | Current | 4,225 | 1,011-6,500 | 0·34 | NR |
| Jacobsen, 2014 [8] | 2014 | Sharkia | Enumeration (time-location geographical mapping) | FSWs | Current | 1,345 | 448-1,416 | 0·34 | NR |
| Jacobsen, 2014 [8] | 2014 | Red Sea | Enumeration (time-location geographical mapping) | FSWs | Current | 1,315 | 404-1,384 | 1·92 | NR |
| Jacobsen, 2014 [8] | 2014 | Menia | Enumeration (time-location geographical mapping) | FSWs | Current | 278 | 89-323 | 0·11 | NR |
| Iran |  |  |  |  |  |  |  |  |  |
| Karami, 2017 [9] | NR | Hamadan | Capture-recapture | FSWs | Past 12 M | 842 | 700-1,042 | 0·45 | NR |
| Sharifi, 2017 [10] | 2015 | Ahvaz | Wisdom of the crowds | FSWs | Current | 10,000 | 5,400 | 2·86 | 1·55-3·86 |
| Sharifi, 2017 [10] | 2015 | Arak | Wisdom of the crowds | FSWs | Current | 3,800 | 2,600 | 2·30 | 1·57-3·38 |
| Sharifi, 2017 [10] | 2015 | Bandar Abbas | Wisdom of the crowds | FSWs | Current | 4,000 | 2,200 | 2·87 | 1·58-4·45 |
| Sharifi, 2017 [10] | 2015 | Isfahan | Wisdom of the crowds | FSWs | Current | 12,200 | 7,800 | 2·02 | 1·29-2·74 |
| Sharifi, 2017 [10] | 2015 | Kerman | Wisdom of the crowds | FSWs | Current | 4,600 | 2,500 | 2·46 | 1·34-3·32 |
| Sharifi, 2017 [10] | 2015 | Kermanshah | Wisdom of the crowds | FSWs | Current | 1,600 | 1,200 | 0·59 | 0·45-1·97 |
| Sharifi, 2017 [10] | 2015 | Mashhad | Wisdom of the crowds | FSWs | Current | 12,000 | 6,700 | 1·43 | 0·80-2·01 |
| Sharifi, 2017 [10] | 2015 | Sari | Wisdom of the crowds | FSWs | Current | 800 | 400 | 0·85 | 0·42-1·17 |
| Sharifi, 2017 [10] | 2015 | Shiraz | Wisdom of the crowds | FSWs | Current | 13,300 | 8,700 | 2·75 | 1·80-3·68 |
| Sharifi, 2017 [10] | 2015 | Tabriz | Wisdom of the crowds | FSWs | Current | 13,100 | 9,000 | 2·84 | 1·95-3·94 |
| Sharifi, 2017 [10] | 2015 | Tehran | Wisdom of the crowds | FSWs | Current | 63,700 | 44,500 | 2·52 | 1·76-3·83 |
| Sharifi, 2017 [10] | 2015 | Zahedan | Wisdom of the crowds | FSWs | Current | 840 | 500 | 0·51 | 0·31-1·41 |
| Sharifi, 2017 [10] | 2015 | Ahvaz | Multiplier unique object | FSWs | Current | 1,200 | 180-8,500 | 0·35 | 0·05-2·43 |
| Sharifi, 2017 [10] | 2015 | Arak | Multiplier unique object | FSWs | Current | 3,000 | 500-21,900 | 1·81 | 0·28-13·2 |
| Sharifi, 2017 [10] | 2015 | Bandar Abbas | Multiplier unique object | FSWs | Current | 390 | 170-900 | 0·28 | 0·12-0·65 |
| Sharifi, 2017 [10] | 2015 | Isfahan | Multiplier unique object | FSWs | Current | 2,300 | 1,150-5,850 | 0·38 | 0·19-0·97 |
| Sharifi, 2017 [10] | 2015 | Kerman | Multiplier unique object | FSWs | Current | 1,400 | 200-9,700 | 0·73 | 0·11-5·17 |
| Sharifi, 2017 [10] | 2015 | Kermanshah | Multiplier unique object | FSWs | Current | 70 | 40-120 | 0·03 | 0·01-0·04 |
| Sharifi, 2017 [10] | 2015 | Khoram Abad | Multiplier unique object | FSWs | Current | 200 | 150-290 | 0·17 | 0·13-0·25 |
| Sharifi, 2017 [10] | 2015 | Mashhad | Multiplier unique object | FSWs | Current | 3,000 | 1,700-5,300 | 0·35 | 0·20-0·63 |
| Sharifi, 2017 [10] | 2015 | Sari | Multiplier unique object | FSWs | Current | 4,700 | 1,000-6,600 | 5·00 | 1·06-7·00 |
| Sharifi, 2017 [10] | 2015 | Shiraz | Multiplier unique object | FSWs | Current | 1,300 | 700-22,700 | 0·26 | 0·13-0·54 |
| Sharifi, 2017 [10] | 2015 | Tabriz | Multiplier unique object | FSWs | Current | 170 | 50-700 | 0·04 | 0·01-0·15 |
| Sharifi, 2017 [10] | 2015 | Tehran | Multiplier unique object | FSWs | Current | 7,500 | 1,600-42,300 | 0·3 | 0·06-1·68 |
| Sharifi, 2017 [10] | 2015 | Ahvaz | Network scale-up | Gen pop | Current | 4,300 | 3,300-5,200 | 1·22 | 0·96-1·47 |
| Sharifi, 2017 [10] | 2015 | Arak | Network scale-up | Gen pop | Current | 2,200 | 1,700-2,600 | 1·30 | 1·05-1·55 |
| Sharifi, 2017 [10] | 2015 | Bandar Abbas | Network scale-up | Gen pop | Current | 2,200 | 1,800-2,500 | 1·56 | 1·31-1·84 |
| Sharifi, 2017 [10] | 2015 | Isfahan | Network scale-up | Gen pop | Current | 14,700 | 13,100-16,500 | 2·44 | 2·16-2·74 |
| Sharifi, 2017 [10] | 2015 | Kerman | Network scale-up | Gen pop | Current | 2,000 | 1,500-2,500 | 1·06 | 0·85-1·31 |
| Sharifi, 2017 [10] | 2015 | Kermanshah | Network scale-up | Gen pop | Current | 4,000 | 3,300-4,700 | 1·47 | 1·23-1·75 |
| Sharifi, 2017 [10] | 2015 | Khoram Abad | Network scale-up | Gen pop | Current | 740 | 570-930 | 0·65 | 0·50-0·80 |
| Sharifi, 2017 [10] | 2015 | Mashhad | Network scale-up | Gen pop | Current | 15,200 | 12,500-18,100 | 1·81 | 1·49-2·16 |
| Sharifi, 2017 [10] | 2015 | Sari | Network scale-up | Gen pop | Current | 1,500 | 1,200-1,700 | 1·54 | 1·30-1·81 |
| Sharifi, 2017 [10] | 2015 | Shiraz | Network scale-up | Gen pop | Current | 8,100 | 7,100-9,100 | 1·67 | 1·46-1·89 |
| Sharifi, 2017 [10] | 2015 | Tabriz | Network scale-up | Gen pop | Current | 640 | 420-930 | 0·14 | 0·09-0·19 |
| Sharifi, 2017 [10] | 2015 | Tehran | Network scale-up | Gen pop | Current | 38,700 | 34,200-43,400 | 1·54 | 1·36-1·71 |
| Sharifi, 2017 [10] | 2015 | Zahedan | Network scale-up | Gen pop | Current | 2,600 | 2,200-3,000 | 1·63 | 1·38-1·88 |
| Karami, 2017 [11] | 2016 | Tehran | Capture-recapture | FSWs | Current | 690 | 633-747 | NR | NR |
| Morocco |  |  |  |  |  |  |  |  |  |
| MOH, 2012 [12] | 2011-12 | Agadir | Multiplier unique object | FSWs | Past 6 M | 3,639-4,333 | 1,556-5,480 | NR | NR |
| MOH, 2012 [12] | 2011-12 | Fes | Multiplier unique object | FSWs | Past 6 M | 6,028 | 3,631-8,504 | NR | NR |
| MOH, 2012 [12] | 2011-12 | Rabat | Multiplier unique object | FSWs | Past 6 M | 5,683 | 4,760-7,333 | NR | NR |
| MOH, 2012 [12] | 2011-12 | Tanger | Multiplier unique object | FSWs | Past 6 M | 3,956 | 3,677-4,234 | NR | NR |
| Huygens, 2013 [13] | 2013 | Agadir | Census | Brothel-based FSWs | Current | 955 | NR | NR | NR |
| Huygens, 2013[13] | 2013 | Agadir | Capture-recapture | FSWs at floating sites | Current | 7,253 | NR | NR | NR |
| Pakistan |  |  |  |  |  |  |  |  |  |
| NACP, 2005 [14] (pilot) | 2004-05 | Karachi | Enumeration (time-location geographical mapping) | Brothel, kothikhana, home, & street-based FSWs | Current | 11,546 | 10,239-12,853 | NR | NR |
| NACP, 2005 [14] (pilot) | 2004-05 | Rawalpindi | Enumeration (time-location geographical mapping) | Kothikhana, home & street-based FSWs | Current | 1,596 | 1,293-1,899 | NR | NR |
| NACP, 2005 [15] (round I) | 2005 | Faisalabad | Enumeration (time-location geographical mapping) | Brothel, kothikhana, home, & street-based FSWs | Current | 2,050 | 1,600-2,500 | 0·46 | NR |
| NACP, 2005 [15] (round I) | 2005 | Hyderabad | Enumeration (time-location geographical mapping) | Brothel, kothikhana, home, & street-based FSWs | Current | 1,350 | 1,200-1,500 | 0·69 | NR |
| NACP, 2005 [15] (round I) | 2005 | Karachi | Enumeration (time-location geographical mapping) | Brothel, kothikhana, home, & street-based FSWs | Current | 11,550 | 10,200-12,900 | 0·58 | NR |
| NACP, 2005 [15] (round I) | 2005 | Lahore | Enumeration (time-location geographical mapping) | Brothel, kothikhana, home, & street-based FSWs | Current | 14,150 | 12,100-16,200 | 1·26 | NR |
| NACP, 2005 [15] (round I) | 2005 | Multan | Enumeration (time-location geographical mapping) | Kothikhana, home & street-based FSWs | Current | 2,500 | 2,000-3,000 | 0·99 | NR |
| NACP, 2005 [15] (round I) | 2005 | Peshawar | Enumeration (time-location geographical mapping) | Kothikhana, home & street-based FSWs | Current | 950 | 800-1,100 | 0·45 | NR |
| NACP, 2005 [15] (round I) | 2005 | Quetta | Enumeration (time-location geographical mapping) | Kothikhana, home & street-based FSWs | Current | 750 | 600-900 | 0·64 | NR |
| NACP, 2005 [15] (round I) | 2005 | Sukkur | Enumeration (time-location geographical mapping) | Kothikhana, home & street-based FSWs | Current | 1,750 | 1,500-2,000 | 0·88 | NR |
| Emmanuel, 2010 [16] (round II) | 2006 | Bannu | Enumeration (time-location geographical mapping) | Kothikhana, home, street-based, & other FSWs | Current | 125 | NR | 0·04 | NR |
| Emmanuel, 2010 [16] (round II) | 2006 | Faisalabad | Enumeration (time-location geographical mapping) | Kothikhana, home, street-based, & other FSWs | Current | 9,500 | NR | 1·30 | NR |
| Emmanuel, 2010 [16] (round II) | 2006 | Gujranwala | Enumeration (time-location geographical mapping) | Kothikhana, home, street-based, & other FSWs | Current | 2,421 | NR | 0·58 | NR |
| Emmanuel, 2010 [16] (round II) | 2006 | Hyderabad | Enumeration (time-location geographical mapping) | Brothel, kothikhana, home, street-based, & other FSWs | Current | 2,750 | NR | 0·71 | NR |
| Emmanuel, 2010 [16] (round II) | 2006 | Karachi | Enumeration (time-location geographical mapping) | Brothel, kothikhana, home, street-based, & other FSWs | Current | 25,550 | NR | 0·74 | NR |
| Emmanuel, 2010 [16] (round II) | 2006 | Lahore | Enumeration (time-location geographical mapping) | Brothel, kothikhana, home, street-based, & other FSWs | Current | 24,625 | NR | 1·34 | NR |
| Emmanuel, 2010 [16] (round II) | 2006 | Larkana | Enumeration (time-location geographical mapping) | Brothel, kothikhana, home, street-based, & other FSWs | Current | 525 | NR | 0·44 | NR |
| Emmanuel, 2010 [16] (round II) | 2006 | Multan | Enumeration (time-location geographical mapping) | Brothel, kothikhana, home, street-based, & other FSWs | Current | 5,075 | NR | 1·22 | NR |
| Emmanuel, 2010 [16] (round II) | 2006 | Peshawar | Enumeration (time-location geographical mapping) | Kothikhana, home, street-based, & other FSWs | Current | 1,550 | NR | 0·44 | NR |
| Emmanuel, 2010 [16] (round II) | 2006 | Quetta | Enumeration (time-location geographical mapping) | Kothikhana, home, street-based, & other FSWs | Current | 2,500 | NR | 1·10 | NR |
| Emmanuel, 2010 [16] (round II) | 2006 | Rawalpindi | Enumeration (time-location geographical mapping) | Kothikhana, home, street-based, & other FSWs | Current | 1,596 | NR | 0·31 | NR |
| Emmanuel, 2010 [16] (round II) | 2006 | Sargodha | Enumeration (time-location geographical mapping) | Kothikhana, home, street-based, & other FSWs | Current | 1,831 | NR | 0·67 | NR |
| Emmanuel, 2010 [16] (round II) | 2006 | Sukkur | Enumeration (time-location geographical mapping) | Kothikhana, home, street-based, & other FSWs | Current | 2,550 | NR | 1·14 | NR |
| Khan, 2011 [17] | 2007 | Lahore | Network scale-up | FSWs | NR | 5,226 | NR | NR | NR |
| Khan, 2011 [17] | 2007 | Lahore | Network scale-up | FSWs (<30 years) | NR | NR | NR | 0·43 | NR |
| Khan, 2011 [17] | 2007 | Lahore | Network scale-up | FSWs (30+ years) | NR | NR | NR | 0·56 | NR |
| NACP, 2008 [18] | 2007 | Faisalabad | Enumeration (time-location geographical mapping) | Adolescent FSWs | Current | 86 | NR | NR | NR |
| NACP, 2008 [18] | 2007 | Karachi | Enumeration (time-location geographical mapping) | Adolescent FSWs | Current | 498 | NR | NR | NR |
| NACP, 2008 [18] | 2007 | Lahore | Enumeration (time-location geographical mapping) | Adolescent FSWs | Current | 9 | NR | NR | NR |
| NACP, 2008 [18] | 2007 | Larkana | Enumeration (time-location geographical mapping) | Adolescent FSWs | Current | 5 | NR | NR | NR |
| NACP, 2008 [18] | 2007 | Mardan | Enumeration (time-location geographical mapping) | Adolescent FSWs | Current | 2 | NR | NR | NR |
| NACP, 2008 [18] | 2007 | Peshawar | Enumeration (time-location geographical mapping) | Adolescent FSWs | Current | 1,030 | NR | NR | NR |
| NACP, 2008 [18] | 2007 | Quetta | Enumeration (time-location geographical mapping) | Adolescent FSWs | Current | 105 | NR | NR | NR |
| Emmanuel, 2013 [19, 20] (round IV) | 2011-12 | DG Khan | Enumeration (time-location geographical mapping) | Kothikhana, home, street-based, & other FSWs | Current | 1,413 | 1,307-1,518 | 1·30 | NR |
| Emmanuel, 2013 [19, 20] (round IV) | 2011-12 | Faisalabad | Enumeration (time-location geographical mapping) | Kothikhana, home, street-based, & other FSWs | Current | 4,846 | 4,381-5,311 | 0·50 | NR |
| Emmanuel, 2013 [19, 20] (round IV) | 2011-12 | Haripur | Enumeration (time-location geographical mapping) | Kothikhana, home, street-based, & other FSWs | Current | 2,994 | 2,850-3,138 | 1·19 | NR |
| Emmanuel, 2013 [19, 20] (round IV) | 2011-12 | Hyderabad | Enumeration (time-location geographical mapping) | Brothel, kothikhana, home, street-based, & other FSWs | Current | 4,566 | 4,018-5,113 | 0·85 | NR |
| Emmanuel, 2013 [19, 20] (round IV) | 2011-12 | Karachi | Enumeration (time-location geographical mapping) | Brothel, kothikhana, home, street-based, & other FSWs | Current | 25,399 | 21,794-29,004 | 0·55 | NR |
| Emmanuel, 2013 [19, 20] (round IV) | 2011-12 | Lahore | Enumeration (time-location geographical mapping) | Brothel, kothikhana, home, street-based, & other FSWs | Current | 23,766 | 21,109-26,422 | 1·15 | NR |
| Emmanuel, 2013 [19, 20] (round IV) | 2011-12 | Larkana | Enumeration (time-location geographical mapping) | Brothel, kothikhana, home, street-based, & other FSWs | Current | 1,114 | 969-1,258 | 0·82 | NR |
| Emmanuel, 2013 [19, 20] (round IV) | 2011-12 | Mirpurkhas | Enumeration (time-location geographical mapping) | Kothikhana, home, street-based, & other FSWs | Current | 884 | 852-915 | 0·85 | NR |
| Emmanuel, 2013 [19, 20] (round IV) | 2011-12 | Multan | Enumeration (time-location geographical mapping) | Brothel, kothikhana, home, street-based, & other FSWs | Current | 5,308 | 4,767-5,847 | 0·80 | NR |
| Emmanuel, 2013 [19, 20] (round IV) | 2011-12 | Nawabshah | Enumeration (time-location geographical mapping) | Brothel, kothikhana, home, street-based, & other FSWs | Current | 2,011 | 1,672-2,352 | 1·42 | NR |
| Emmanuel, 2013 [19, 20] (round IV) | 2011-12 | Peshawar | Enumeration (time-location geographical mapping) | Kothikhana, home, street-based, & other FSWs | Current | 3,317 | 2,897-3,736 | 0·42 | NR |
| Emmanuel, 2013 [19, 20] (round IV) | 2011-12 | Quetta | Enumeration (time-location geographical mapping) | Kothikhana, home, street-based, & other FSWs | Current | 3,710 | 3,271-4,149 | 1·07 | NR |
| Emmanuel, 2013 [19, 20] (round IV) | 2011-12 | Rawalpindi | Enumeration (time-location geographical mapping) | Kothikhana, home, street-based, & other FSWs | Current | 3,635 | 3,263-4,021 | 0·34 | NR |
| Emmanuel, 2013 [19, 20] (round IV) | 2011-12 | Sargodha | Enumeration (time-location geographical mapping) | Brothel, kothikhana, home, street-based, & other FSWs | Current | 3,898 | 3,597-4,198 | 1·25 | NR |
| Emmanuel, 2013 [19, 20] (round IV) | 2011-12 | Sukkur | Enumeration (time-location geographical mapping) | Kothikhana, home, street-based, & other FSWs | Current | 2,317 | 2,031-2,610 | 1·05 | NR |
| Punjab ACP, 2015 [21] | 2014 | Faisalabad | Enumeration (time-location geographical mapping) | Kothikhana, home, street-based, & other FSWs | Current | 7,556 | 5,500-9,612 | NR | NR |
| Punjab ACP, 2015 [21] | 2014 | Lahore | Enumeration (time-location geographical mapping) | Brothel, kothikhana, home, street-based, & other FSWs | Current | 25,716 | 21,685-29,746 | NR | NR |
| Punjab ACP, 2015 [21] | 2014 | Multan | Enumeration (time-location geographical mapping) | Brothel, kothikhana, home, street-based, & other FSWs | Current | 6,561 | 4,272-8,850 | NR | NR |
| Punjab ACP, 2015 [21] | 2014 | Sargodha | Enumeration (time-location geographical mapping) | Brothel, kothikhana, home, street-based, & other FSWs | Current | 4,327 | 2,987-5,667 | NR | NR |
| NACP, 2017 [22] (round V) | 2016-17 | Bahawalpur | Enumeration (time-location geographical mapping) | Brothel, kothikhana, home, street-based, & other FSWs | Current | 6,201 | 5,522-6,737 | NR | NR |
| NACP, 2017 [22] (round V) | 2016-17 | Bannu | Enumeration (time-location geographical mapping) | Brothel, kothikhana, home, street-based, & other FSWs | Current | 192 | 171-209 | NR | NR |
| NACP, 2017 [22] (round V) | 2016-17 | DG Khan | Enumeration (time-location geographical mapping) | Kothikhana, home, street-based, & other FSWs | Current | 1,349 | 1,201-1,466 | NR | NR |
| NACP, 2017 [22] (round V) | 2016-17 | Gujranwala | Enumeration (time-location geographical mapping) | Brothel, kothikhana, home, street-based, & other FSWs | Current | 4,069 | 3,624-4,420 | NR | NR |
| NACP, 2017 [22] (round V) | 2016-17 | Gujrat | Enumeration (time-location geographical mapping) | Brothel, kothikhana, home, street-based, & other FSWs | Current | 317 | 282-344 | NR | NR |
| NACP, 2017 [22] (round V) | 2016-17 | Hyderabad | Enumeration (time-location geographical mapping) | Brothel, kothikhana, home, street-based, & other FSWs | Current | 4426 | 3,942-4,808 | NR | NR |
| NACP, 2017 [22] (round V) | 2016-17 | Karachi | Enumeration (time-location geographical mapping) | Brothel, kothikhana, home, street-based, & other FSWs | Current | 25,191 | 22,434-27,367 | NR | NR |
| NACP, 2017 [22] (round V) | 2016-17 | Kasur | Enumeration (time-location geographical mapping) | Brothel, kothikhana, home, street-based, & other FSWs | Current | 1,739 | 1,549-1,889 | NR | NR |
| NACP, 2017 [22] (round V) | 2016-17 | Larkana | Enumeration (time-location geographical mapping) | Brothel, kothikhana, home, street-based, & other FSWs | Current | 4,593 | 4,090-4,990 | NR | NR |
| NACP, 2017 [22] (round V) | 2016-17 | Mirpurkhas | Enumeration (time-location geographical mapping) | Kothikhana, home, street-based, & other FSWs | Current | 2,084 | 1,856-2,264 | NR | NR |
| NACP, 2017 [22] (round V) | 2016-17 | Nawabshah | Enumeration (time-location geographical mapping) | Brothel, kothikhana, home, street-based, & other FSWs | Current | 1,690 | 1,505-1,836 | NR | NR |
| NACP, 2017 [22] (round V) | 2016-17 | Peshawar | Enumeration (time-location geographical mapping) | Kothikhana, home, street-based, & other FSWs | Current | 765 | 681-831 | NR | NR |
| NACP, 2017 [22] (round V) | 2016-17 | Rawalpindi | Enumeration (time-location geographical mapping) | Kothikhana, home, street-based, & other FSWs | Current | 2,465 | 2,195-2,678 | NR | NR |
| NACP, 2017 [22] (round V) | 2016-17 | Quetta | Enumeration (time-location geographical mapping) | Kothikhana, home, street-based, & other FSWs | Current | 4,121 | 3,670-4,477 | NR | NR |
| NACP, 2017 [22] (round V) | 2016-17 | Sheikhupura | Enumeration (time-location geographical mapping) | Brothel, kothikhana, home, street-based, & other FSWs | Current | 6,252 | 5,568-6,792 | NR | NR |
| NACP, 2017 [22] (round V) | 2016-17 | Sialkot | Enumeration (time-location geographical mapping) | Brothel, kothikhana, home, street-based, & other FSWs | Current | 2,031 | 1,809-2,206 | NR | NR |
| NACP, 2017 [22] (round V) | 2016-17 | Sukkur | Enumeration (time-location geographical mapping) | Kothikhana, home, street-based, & other FSWs | Current | 3,307 | 2,945-3,593 | NR | NR |
| NACP, 2017 [22] (round V) | 2016-17 | Turbat | Enumeration (time-location geographical mapping) | Brothel, kothikhana, home, street-based, & other FSWs | Current | 523 | 466-568 | NR | NR |
| Somalia |  |  |  |  |  |  |  |  |  |
| WHO, 2011[23] | 2011 | Berbera & Bossaso | NR | FSWs | Current | 614 | NR | NR | NR |
| MOH, 2016 [24] | 2016 | Bossaso | Enumeration (time-location geographical mapping) | FSWs | Past 12 M | 911 | 736-1,079 | NR | NR |
| MOH, 2016 [24] | 2016 | Hargeisa | Enumeration (time-location geographical mapping) | FSWs | Past 12 M | 1,126 | 842-1,409 | NR | NR |
| MOH, 2016 [24] | 2016 | Mogadishu | Multiplier unique object | FSWs | Past 12 M | 963 | NR | NR | NR |
| Sudan |  |  |  |  |  |  |  |  |  |
| NACP, 2002 [25] | 2002 | Khartoum, Gezira, Kassala | Pop-bsd survey (self-report) | Refugees (predom. women) | Past 12 M | NR | NR | 0·83 | NR |
| NACP, 2002 [25] | 2002 | Khartoum, Gezira, Kassala | Conv sample (self-report) | ANC attendees | Past 12 M | NR | NR | 0·5 | NR |
| NACP, 2005 [26] | 2005 | South Darfur | Conv sample (self-report) | Tea and food sellers | Lifetime | NR | NR | 3·00 | NR |
| UNHCR, 2007 [27] | 2006 | Juba, South Sudan | Pop-bsd survey (self-report) | Gen pop (15-49 years) | Lifetime | NR | NR | 0·4 | NR |
| UNHCR, 2007 [27] | 2006 | Juba, South Sudan | Pop-bsd survey (self-report) | Gen pop (15-49 years) | Past 12 M | NR | NR | 0·2 | NR |
| NAP, 2015 [28] | 2008 | Juba, South Sudan | Conv sample (self-report) | Gen pop | Past 12 M | NR | NR | 10 | NR |
| NAP, 2015 [28] | 2008 | Morobo, South Sudan | Conv sample (self-report) | Gen pop | Past 12 M | NR | NR | 13 | NR |
| WHO, 2011 [23] | 2012 | Juba, South Sudan | NR | FSWs | Current | 2,511 | NR | NR | NR |
| WHO, 2011 [23] | 2012 | Yambio, South Sudan | NR | FSWs | Current | 375 | NR | NR | NR |
| NAP, 2016 [29] | 2015 | Juba, Yei, & Nimule, South Sudan | NR | FSWs | NR | 4,700 | NR | NR | NR |
| MOH, 2016 [30] | 2015-16 | Juba, South Sudan | Multiplier unique object | FSWs | Past 6 M | 5,800 | 4,927-6,673 | NR | NR |
| MOH, 2016 [30] | 2015-16 | Juba, South Sudan | Capture-recapture | FSWs | Past 6 M | 5,306 | 4,673-5,939 | NR | NR |
| Tunisia |  |  |  |  |  |  |  |  |  |
| Hsairi, 2012 [31] | 2011 | Tunis | Multiplier unique object | Street-based FSWs | Current | 541 | 447-681 | NR | NR |
| Hsairi, 2012 [31] | 2011 | Sfax | Multiplier unique object | Street-based FSWs | Current | 596 | 477-795 | NR | NR |
| Hsairi, 2012 [31] | 2011 | Sousse | Multiplier unique object | Street-based FSWs | Current | 291 | 250-350 | NR | NR |
| Yemen |  |  |  |  |  |  |  |  |  |
| MOH, 2010 [32] | NR | Aden | Enumeration (time-location geographical mapping) | FSWs | Current | NR | 1,875-4,260 | NR | 1·16-2·64 |
| MOH, 2010 [32] | NR | Hodeida | Enumeration (time-location geographical mapping) | FSWs | Current | NR | 1,580-1,759 | NR | 1·89-2·10 |
| MOH, 2010 [32] | NR | Mukallah | Enumeration (time-location geographical mapping) | FSWs | Current | NR | 1,488-1,786 | NR | 2·07-2·49 |
| MOH, 2010 [32] | NR | Sanaa | Enumeration (time-location geographical mapping) | FSWs | Current | NR | 3,092-4,495 | NR | 0·64-2·10 |
| MOH, 2010 [32] | NR | Taiz | Enumeration (time-location geographical mapping) | FSWs | Current | NR | 1,050-1,835 | NR | 0·80-1·40 |
| Clients of FSWs |  |  |  |  |  |  |  |  |  |
| Afghanistan |  |  |  |  |  |  |  |  |  |
| Mansoor, 2008[33] | 2007 | Balkh, Herat, Kabul, & Nangahar | Pop-bsd survey (self-report) | Freshmen students | Past 12 M | NR | NR | 5·2 | NR |
| Djibouti |  |  |  |  |  |  |  |  |  |
| Trellu-Kane, 2005[7] | 2005 | Djibouti | Conv sample (self-report) | Gen pop (13-24 years) | Past 12 M | NR | NR | 17 | NR |
| Iran |  |  |  |  |  |  |  |  |  |
| Shokoohi, 2012[34] | NR | Kerman | Network scale-up, (probability method) based on conv sample | Gen pop | Past 12 M | 9,314 | 7,710-10,916 | 7·0 | 5·8-8·2 |
| Shokoohi, 2012 [34] | NR | Kerman | Network scale-up, (frequency method) based on conv sample | Gen pop | Past 12 M | 3,203 | 1,704-5,130 | 2·4 | 1·3-3·9 |
| Khalajabadi, 2018 [35] | 2013-14 | Tehran | Pop-bsd survey (self-report) | University students | Last sex | NR | NR | 1·3 | NR |
| Khalajabadi, 2018 [35] | 2013-14 | Tehran | Pop-bsd survey (self-report) | University students | Lifetime | NR | NR | 6·6 | NR |
| Lebanon |  |  |  |  |  |  |  |  |  |
| Melikian, 1954 [36] | 1952 | Beirut | Conv sample (self-report) | University students in a liberal and comparatively Western college student environment | Past 12 M | NR | NR | 59·3 | NR |
| Melikian, 1967[37] | 1963 | Beirut | Conv sample (self-report) | University students in a liberal and comparatively Western college student environment | Past 12 M | NR | NR | 40·6 | NR |
| Ghandour, 2014[38] | 2012 | Beirut | Pop-bsd survey (self-report) | University students (18-30 years) | Lifetime paid sex | NR | NR | 20·1 | NR |
| Pakistan |  |  |  |  |  |  |  |  |  |
| Faisel, 2005 [39] | 2004-05 | Lahore | Pop-bsd survey (self-report) | Migrant workers | Past 12 M | NR | NR | 6·8 | NR |
| Minhas, 2005 [40] | 2005 | NR | Self-report (conv sample) | Students | Current | NR | NR | 7 | NR |
| Somalia |  |  |  |  |  |  |  |  |  |
| Ismail, 1990[41] | 1986 | Mogadishu | Self-report (conv sample) | Healthcare workers and medical students | NR | NR | NR | 48 | NR |
| Ismail, 1990 [42] | 1987 | Jambaluul village | Conv sample (self-report; take all) | Gen pop | Lifetime | NR | NR | 29 | NR |
| MOH, 2016 [24] | 2016 | Bossaso | Enumeration (time-location geographical mapping) | Secondary key informants | Past 12 M | 3,469 | 2,480-4,453 | NR | NR |
| MOH, 2016 [24] | 2016 | Bossaso | Wisdom of the crowds | Gen pop | Past 12 M | 3,530 | NR | NR | NR |
| MOH, 2016 [24] | 2016 | Hargeisa | Enumeration (time-location geographical mapping) | Secondary key informants | Past 12 M | 1,828 | 1,301-2,353 | NR | NR |
| MOH, 2016 [24] | 2016 | Hargeisa | Wisdom of the crowds | Gen pop | Past 12 M | 1,559 | NR | NR | NR |
| MOH, 2016 [24] | 2016 | Mogadishu | Enumeration (time-location geographical mapping) | Secondary key informants | Past 12 M | 2,599 | 1,801-3,395 | NR | NR |
| MOH, 2016 [24] | 2016 | Mogadishu | Wisdom of the crowds | Gen pop | Past 12 M | 2,202 | NR | NR | NR |
| Sudan |  |  |  |  |  |  |  |  |  |
| McCarthy, 1989[43] | 1987-88 | Port Sudan, Kassala, Gederef, Juba & Omdurman | Conv sample (self-report) | Soldiers attending outpatient military clinics | Lifetime | NR | NR | 51·6 | NR |
| Holt, 2003 [44] | 1992 | Dimma refugee camp | Conv sample (self-report) | Sudanese refugees | Lifetime | NR | NR | 46·0 | 39·0-53·0 |
| Holt, 2003 [44] | 1992 | Dimma refugee camp | Conv sample (self-report) | Sudanese refugees | Past 3 M | NR | NR | 31·0 | 25·0-38·0 |
| NACP, 2002 [25] | 2002 | Blue Nile & Equatoria | Conv sample (self-report) | Military personnel | Past 12 M | NR | NR | 11·7 | NR |
| UNHCR, 2007 [27] | 2006 | Juba, South Sudan | Pop-bsd survey (self-report) | Gen pop (15-49 years) | Lifetime | NR | NR | 1·7 | NR |
| UNHCR, 2007 [27] | 2006 | Juba, South Sudan | Pop-bsd survey (self-report) | Gen pop (15-49 years) | Past 12 M | NR | NR | 1·4 | NR |
| United Arab Emirates |  |  |  |  |  |  |  |  |  |
| MOH, 2014 [45] | 2010-11 | NR | Conv sample (self-report) | University students | Lifetime | NR | NR | 0·07 | NR |

The table is sorted by year(s) of data collection or year of publication if year of data collection was not reported.

^*^The decimal places of the population proportion figures are as reported in the original reports.

*Abbreviations*: *ACP* AIDS Control Program, *ANC* antenatal clinic, *Conv* convenience, *DG Khan* Dera Ghazi Khan, *Gen* general, *FSWs* female sex workers, *M* months, *MOH* ministry of Health, *NACP* National AIDS Control Programme, *NAP* National AIDS Program, *NR* not reported, *Pop* population, *Pop-bsd* population-based, *SAR AIDS HDS* South Asia Region AIDS Human Development Sector, *UNHCR* United Nations Higher Commission for Refugees, *WHO* World Health Organization

**Table S5** HIV point-prevalence measures in FSWs as extracted or obtained from various sources including the US Census Bureau database, the WHO-EMRO, and the UNAIDS epidemiological fact sheets databases, among other sources of data

| **Country**  **Author, year [citation]** | **Year(s) of data collection** | **City/province** | **Study site** | **Sampling** | **Population** | **Sample size** | **HIV prev**^*^ **(%)** |
| --- | --- | --- | --- | --- | --- | --- | --- |
| **Afghanistan** |  |  |  |  |  |  |  |
| MENA HIV ESP, 2013[46] | 2011-12 | National | NR | NR | FSWs | 487 | 0 |
| MENA HIV ESP, 2013 [46] | 2012 | National | NR | NR | FSWs | 1039 | 0·3 |
| **Algeria** |  |  |  |  |  |  |  |
| Abu-Raddad, 2010 [2] | 2004 | NR | NR | NR | FSWs | NR | 3·0 |
| Abu-Raddad, 2010 [2] | 2004 | NR | NR | NR | FSWs | NR | 4·0 |
| Jenkins, 2003 [47] | 1988 | NR | NR | NR | FSWs | NR | 1·2 |
| MOH, 1990 [48] | 1988 | Oran | NR | Conv | FSWs | 52 | 1·9 |
| MOH, 1990 [48] | 1988 | Blida | NR | Conv | FSWs | 34 | 0 |
| MOH, 1990 [48] | 1988 | Tlemcen | NR | Conv | FSWs | 43 | 0 |
| MOH, 1990 [48] | 1988 | Ghardaia | NR | Conv | FSWs | 19 | 0 |
| MOH, 1990 [48] | 1988 | Biskra | NR | Conv | FSWs | 13 | 7·7 |
| MOH, 1990 [48] | 1988 | Constantine | NR | Conv | FSWs | 237 | 0·4 |
| MOH, 1990 [48] | 1988 | Tindouf | NR | Conv | FSWs | 11 | 0 |
| Addad, 1993 [49] | 1991 | NR | NR | NR | FSWs | NR | 0 |
| Jenkins, 2003 [47] | 2000 | NR | NR | NR | FSWs | 20 | 10 |
| MOH, 2009 [50] | 2000 | Tamanrasset & Oran | Sentinel surveillance | Conv | FSWs | 139 | 2·9 |
| UNAIDS, 2008 [51] | 2000 | Tamanrasset | NR | NR | FSWs | NR | 20 |
| Abu-Raddad, 2010 [2] | 2004 | NR | NR | NR | FSWs | NR | 2·0 |
| MOH, 2009 [50] | 2004 | National | Sentinel surveillance | Conv | FSWs | 185 | 3·8 |
| MOH, 2009 [50] | 2007 | National | Sentinel surveillance | Conv | FSWs | 380 | 4·0 |
| MOH, 2016 [52] | 2008 | Tamanrasset | Sentinel surveillance | Conv | FSWs | 161 | 1·2 |
| MOH, 2016 [52] | 2012 | Tamanrasset | Sentinel surveillance | Conv | FSWs | 109 | 4·6 |
| MOH, 2014 [53] | 2014 | Saida | Sentinel surveillance | Conv | FSWs | 78 | 5·1 |
| MOH, 2017 [54] | 2017 | NR | NR | NR | FSWs | NR | 5·5 |
| MOH, 2018 [55] | 2018 | NR | NR | NR | FSWs | NR | 4·2 |
| **Bahrain** |  |  |  |  |  |  |  |
| MOH, 2012 [56] | 2010-11 | National | Detainment center | Conv | FSWs tested at detainment | 724 | 0·8 |
| **Djibouti** |  |  |  |  |  |  |  |
| Jenkins, 2003 [47] | 1987 | NR | NR | NR | Street-based FSWs | NR | 3·9 |
| UNAIDS, 2008 [51] | 1987 | Djibouti | NR | NR | FSWs | NR | 2·1 |
| Bailly, 1988 [57] | 1987-88 | NR | NR | NR | FSWs | 251 | 2·8 |
| UNAIDS, 2008 [51] | 1988 | Djibouti | NR | NR | FSWs | NR | 4·2 |
| MENA HIV ESP, 2010 [2] | 1989 | NR | Sentinel surveillance | Conv | FSWs | 560 | 5·2 |
| MENA HIV ESP, 2010 [2] | 1989 | NR | Sentinel surveillance | Conv | Bar girls | 476 | 2·1 |
| MENA HIV ESP, 2010 [2] | 1990 | NR | Sentinel surveillance | Conv | Bar girls | 190 | 5·8 |
| UNAIDS, 2008 [51] | 1990 | Djibouti (Major urban areas) | NR | NR | FSWs | NR | 19·5 |
| Jenkins, 2003 [47] | 1991 | NR | NR | NR | Bar girls | NR | 14·2 |
| MENA HIV ESP, 2010 [2] | 1991 | NR | Sentinel surveillance | Conv | FSWs | 449 | 31·4 |
| MENA HIV ESP, 2010 [2] | 1991 | NR | Sentinel surveillance | Conv | Bar girls | 618 | 13·1 |
| OMS, 2001 [58] | 1991 | NR | NR | NR | FSWs | NR | 39·8 |
| UNAIDS, 2008 [51] | 1991 | Djibouti (Major urban areas) | NR | NR | FSWs | NR | 26·0 |
| MENA HIV ESP, 2010 [2] | 1992 | NR | Sentinel surveillance | Conv | FSWs | 400 | 43·0 |
| MENA HIV ESP, 2010 [2] | 1992 | NR | Sentinel surveillance | Conv | Bar girls | 724 | 12·4 |
| MOH, 1993[59] | 1992 | NR | NR | NR | Street-based FSWs | NR | 51·4 |
| MOH, 1993 [59] | 1992 | NR | NR | NR | Bar girls | NR | 21·7 |
| UNAIDS, 2008[51] | 1992 | Djibouti (Major urban areas) | NR | NR | FSWs | NR | 36·6 |
| Jenkins, 2003 [47] | 1993 | NR | NR | NR | Bar girls | NR | 25·6 |
| Jenkins, 2003 [47] | 1993 | NR | NR | NR | Street-based FSWs | NR | 55·8 |
| MOH, 1993 [59] | 1993 | NR | NR | NR | Bar girls | 411 | 23·4 |
| MOH, 1993 [59] | 1993 | NR | NR | NR | Street-based FSWs | 313 | 56·5 |
| OMS, 2001 [58] | 1993 | NR | NR | NR | Bar girls | NR | 27·0 |
| Shrestha, 1999 [60] | 1993 | NR | Bars | NR | Bar girls | 1039 | 14·7 |
| Shrestha, 1999 [60] | 1993 | NR | NR | NR | FSWs | 571 | 47·5 |
| UNAIDS, 2008 [51] | 1993 | Djibouti (Major urban areas) | NR | NR | FSWs | NR | 37·7 |
| UNAIDS, 2008 [51] | 1993 | Outside major urban areas | NR | NR | FSWs | NR | 26·3 |
| UNAIDS, 2008 [51] | 1993 | Outside major urban areas | NR | NR | FSWs | NR | 0·1 |
| Shrestha, 1999 [60] | 1994 | NR | Bars | NR | Bar girls | 852 | 12·2 |
| Shrestha, 1999 [60] | 1994 | NR | NR | NR | FSWs | 573 | 45·4 |
| UNAIDS, 2008 [51] | 1994 | Outside major urban areas | NR | NR | FSWs | NR | 25·5 |
| UNAIDS, 2008 [51] | 1994 | Outside major urban areas | NR | NR | FSWs | NR | 0 |
| Shrestha, 1999 [60] | 1995 | NR | Bars | NR | Bar girls | 68 | 11·8 |
| UNAIDS, 2008 [51] | 1995 | Outside major urban areas | NR | NR | FSWs | NR | 36·8 |
| UNAIDS, 2008 [51] | 1995 | Outside major urban areas | NR | NR | FSWs | NR | 0·1 |
| Shrestha, 1999 [60] | 1995 | NR | NR | NR | FSWs | 364 | 41·5 |
| US Dep. of State, 2000 [61] | 1995 | NR | NR | NR | FSWs | NR | 57·0 |
| Shrestha, 1999 [60] | 1996 | NR | NR | NR | FSWs | 294 | 32·7 |
| UNAIDS, 2008 [51] | 1996 | Outside major urban areas | NR | NR | FSWs | NR | 0 |
| Shrestha, 1999 [60] | 1997 | NR | NR | NR | FSWs | 327 | 32·7 |
| UNAIDS, 2008 [51] | 1997 | Outside major urban areas | NR | NR | FSWs | NR | 0 |
| Bahdon, 1998 [62] | 1998 | NR | NR | NR | FSWs | 117 | 28·2 |
| MOH, 1999 [63] | 1998 | NR | NR | NR | FSWs | 142 | 27·5 |
| UNAIDS, 2008 [51] | 1998 | Outside major urban areas | NR | NR | FSWs | NR | 0 |
| MENA HIV ESP, 2010 [2] | 1999 | NR | Sentinel surveillance | Conv | FSWs | 42 | 38·1 |
| UNAIDS, 2008 [51] | 1999 | Outside major urban areas | NR | NR | FSWs | NR | 0 |
| UNAIDS, 2008 [51] | 1999 | Outside major urban areas | NR | NR | FSWs | NR | 0 |
| MENA HIV ESP, 2010 [2] | 2000 | NR | Sentinel surveillance | Conv | FSWs | 34 | 20·6 |
| MOH, 2008 [64] | 2007 | NR | NR | NR | FSWs | 66 | 19·7 |
| MOH, 2010 [65] | 2007 | NR | Sentinel surveillance | Conv | FSWs | NR | 18·0 |
| MOH, 2008 [64] | 2008 | NR | NR | NR | FSWs | 52 | 17·3 |
| WHO, 2011 [23] | 2008 | NR | Clinics | Conv | FSWs | 79 | 20·3 |
| MOH, 2010 [65] | 2009 | NR | Sentinel surveillance | Conv | FSWs | NR | 15·3 |
| MENA HIV ESP, 2013 [46] | 2012 | Djibouti | Clinical center | Conv | FSWs | 718 | 13·1 |
| **Egypt** |  |  |  |  |  |  |  |
| MENA HIV ESP, 2010 [2] | 1989 | NR | Sentinel surveillance | Conv | FSWs | 347 | 0 |
| Mourad, 1992 [66] | 1990-91 | Cairo | NR | NR | FSWs | 154 | 0 |
| MOH, 2001 [67] | 1992 | NR | NR | Conv | FSWs | 160 | 0 |
| MOH, 2001 [67] | 1993 | NR | NR | Conv | FSWs | 221 | 0 |
| Murugasampillay, 1995[68] | 1993 | Alexandria | NR | Conv | FSWs | 42 | 0 |
| MOH, 2001 [67] | 1994 | NR | NR | Conv | FSWs | 194 | 0 |
| MOH, 2001 [67] | 1995 | NR | NR | Conv | FSWs | 129 | 0 |
| MENA HIV ESP, 2010 [2] | 1996 | NR | Sentinel surveillance | Conv | FSWs | 145 | 0·7 |
| MOH, 2001 [67] | 1996 | NR | NR | Conv | FSWs | 112 | 0·9 |
| MENA HIV ESP, 2010 [2] | 1997 | NR | Sentinel surveillance | Conv | FSWs | 79 | 0 |
| MOH, 2001 [67] | 1997 | NR | NR | Conv | FSWs | 179 | 1·1 |
| MENA HIV ESP, 2010 [2] | 1998 | NR | Sentinel surveillance | Conv | FSWs | 69 | 0 |
| MOH, 2001 [67] | 1998 | NR | NR | Conv | FSWs | 269 | 1·5 |
| MENA HIV ESP, 2010 [2] | 1999 | NR | Sentinel surveillance | Conv | FSWs | 172 | 0·6 |
| MOH, 2001 [67] | 1999 | NR | NR | Conv | FSWs | 183 | 1·1 |
| MOH, 2001 [67] | 2000 | NR | NR | Conv | FSWs | 129 | 0 |
| MOH, 2001 [67] | 2001 | NR | NR | Conv | FSWs | 65 | 0 |
| MENA HIV ESP, 2010 [2] | 2002 | NR | Sentinel surveillance | Conv | FSWs | 203 | 0 |
| MENA HIV ESP, 2010 [2] | 2003 | NR | Sentinel surveillance | Conv | FSWs | 265 | 0 |
| MENA HIV ESP, 2010 [2] | 2003 | NR | Sentinel surveillance | Conv | Bar girls | 181 | 0 |
| MENA HIV ESP, 2010 [2] | 2004 | NR | Sentinel surveillance | Conv | FSWs | 345 | 0 |
| US Census Bureau, 2017 [69] | 2004 | NR | Sentinel surveillance | Conv | FSWs | 308 | 0 |
| MENA HIV ESP, 2010 [2] | 2005 | NR | Sentinel surveillance | Conv | FSWs | 192 | 0 |
| MENA HIV ESP, 2010 [2] | 2006 | National | NR | NR | FSWs & bar girls | 975 | 0 |
| Arafa, 2007 [70] | 2006-07 | Alexandria | Clinic | Conv | FSWs | NR | 0 |
| NAP, 2014 [71] | 2010 | Cairo | NGO | Conv | FSWs | 137 | 0 |
| NAP, 2014 [71] | 2013 | NR | VCT | Conv | FSWs | 188 | 0·5 |
| NAP, 2017 [72] | 2016 | NR | Sentinel surveillance | Conv | FSWs | 249 | 1·2 |
| **Iran** |  |  |  |  |  |  |  |
| NACP, 1994 [73] | 1987-91 | NR | Sentinel surveillance | Conv | FSWs | 3596 | 0·03 |
| MENA HIV ESP, 2010 [2] | 1990 | NR | Sentinel surveillance | Conv | FSWs | 708 | 0·1 |
| MENA HIV ESP, 2010 [2] | 1991-92 | NR | Sentinel surveillance | Conv | FSWs | 2897 | 0 |
| NACP, 1994 [73] | 1993-94 | Evin | Sentinel surveillance | Conv | FSWs | 400 | 0 |
| Eltayeb, 1995 [74] | 1994 | NR | Rehab. centers | Conv | FSWs | 31 | 0 |
| Shrestha, 1999 [60] | 1995 | NR | NR | NR | FSWs | 505 | 0 |
| Shrestha, 1999 [60] | 1996 | NR | NR | NR | FSWs | 120 | 0 |
| Shrestha, 1999 [60] | 1997 | NR | NR | NR | FSWs | 220 | 0 |
| Shrestha, 1999 [60] | 1998 | NR | NR | NR | FSWs | 1605 | 0 |
| MENA HIV ESP, 2010 [2] | 1999 | NR | Sentinel surveillance | Conv | FSWs | 800 | 0 |
| Feizzadeh, 2010 [75] | 2000 | Charmanhal | Prison | Conv | Incarcerated FSWs | NR | 14 |
| MENA HIV ESP, 2010 [2] | 2000-01 | NR | Sentinel surveillance | Conv | FSWs | 404 | 0 |
| MENA HIV ESP, 2010 [2] | 2002 | NR | Sentinel surveillance | Conv | FSWs | 309 | 0 |
| MENA HIV ESP, 2010 [2] | 2003-05 | NR | Sentinel surveillance | Conv | FSWs | 44 | 2·3 |
| MENA HIV ESP, 2010 [2] | 2005 | Isfahan | Sentinel surveillance | Conv | FSWs | 258 | 0 |
| MOH, 2006 [76] | 2005 | Tehran | NR | Conv | FSWs | 50 | 0 |
| MENA HIV ESP, 2010 [2] | 2006 Q1 & Q3 | National | NR | NR | FSWs & bar girls | 301 | 2·7 |
| MENA HIV ESP, 2010 [2] | 2006 | Isfhan | Sentinel surveillance | Conv | FSWs | 281 | 0 |
| Feizzadeh, 2010 [75] | 2007 | Kermanshah | PHC | Conv | FSWs attending clinics | NR | 3 |
| Feizzadeh, 2010 [75] | 2007 | Kohkilouye | Prison | Conv | Incarcerated FSWs | NR | 11 |
| **Iraq** |  |  |  |  |  |  |  |
| MENA HIV ESP, 2010 [2] | 1989 | NR | Sentinel surveillance | Conv | Bar girls | 300 | 0 |
| MENA HIV ESP, 2010 [2] | 1989 | NR | Sentinel surveillance | Conv | FSWs | 420 | 0 |
| MENA HIV ESP, 2010 [2] | 1990 | NR | Sentinel surveillance | Conv | Bar girls | 429 | 0 |
| MENA HIV ESP, 2010 [2] | 1990 | NR | Sentinel surveillance | Conv | FSWs | 678 | 0 |
| MENA HIV ESP, 2010 [2] | 1991 | NR | Sentinel surveillance | Conv | Bar girls | 334 | 0 |
| MENA HIV ESP, 2010 [2] | 1991 | NR | Sentinel surveillance | Conv | FSWs | 225 | 0 |
| MENA HIV ESP, 2010 [2] | 1992 | NR | Sentinel surveillance | Conv | Bar girls | 369 | 0 |
| MENA HIV ESP, 2010 [2] | 1992 | NR | Sentinel surveillance | Conv | FSWs | 14 | 0 |
| Shrestha, 1999 [60] | 1993 | NR | NR | NR | Bar girls | 1337 | 0 |
| Shrestha, 1999 [60] | 1993 | NR | NR | NR | FSWs | 987 | 0 |
| Shrestha, 1999 [60] | 1994 | NR | NR | NR | Bar girls | 1083 | 0 |
| Shrestha, 1999 [60] | 1994 | NR | NR | NR | FSWs | 1084 | 0 |
| Shrestha, 1999 [60] | 1995 | NR | NR | NR | Bar girls | 876 | 0 |
| Shrestha, 1999 [60] | 1995 | NR | NR | NR | FSWs | 1408 | 0 |
| Shrestha, 1999 [60] | 1996 | NR | NR | NR | Bar girls | 472 | 0 |
| Shrestha, 1999 [60] | 1996 | NR | NR | NR | FSWs | 1272 | 0·07 |
| Shrestha, 1999 [60] | 1997 | NR | NR | NR | Bar girls | 582 | 0 |
| Shrestha, 1999 [60] | 1997 | NR | NR | NR | FSWs | 475 | 0 |
| Shrestha, 1999 [60] | 1998 | NR | NR | NR | Bar girls | 1027 | 0 |
| Shrestha, 1999 [60] | 1998 | NR | NR | NR | FSWs | 12 | 0 |
| Shrestha, 1999 [60] | 1998 | NR | NR | NR | Bar girls | 33 | 0 |
| MENA HIV ESP, 2010 [2] | 1999 | NR | Sentinel surveillance | Conv | Bar girls | 98 | 0 |
| MENA HIV ESP, 2010 [2] | 1999 | NR | Sentinel surveillance | Conv | FSWs | 1255 | 0 |
| MENA HIV ESP, 2010 [2] | 2000 | NR | Sentinel surveillance | Conv | Bar girls | 87 | 0 |
| MENA HIV ESP, 2010 [2] | 2000 | NR | Sentinel surveillance | Conv | FSWs | 199 | 0 |
| MENA HIV ESP, 2010 [2] | 2001 | NR | Sentinel surveillance | Conv | Bar girls | 153 | 0 |
| MENA HIV ESP, 2010 [2] | 2001 | NR | Sentinel surveillance | Conv | FSWs | 253 | 0 |
| MENA HIV ESP, 2010 [2] | 2002 | NR | Sentinel surveillance | Conv | Bar girls | 96 | 0 |
| MENA HIV ESP, 2010 [2] | 2002 | NR | Sentinel surveillance | Conv | FSWs | 294 | 0 |
| **Jordan** |  |  |  |  |  |  |  |
| El-Tayeb, 1995 [77] | 1990 | NR | NR | NR | FSWs | 40 | 0 |
| El-Tayeb, 1995 [77] | 1991 | NR | NR | NR | FSWs | 75 | 1·3 |
| El-Tayeb, 1995 [77] | 1994-95 | NR | NR | NR | FSWs | 12 | 0 |
| **Lebanon** |  |  |  |  |  |  |  |
| NACP, 1994 [78] | 1987-89 | National | NR | Conv | FSWs | 741 | 0 |
| NACP, 1994 [78] | 1992 | National | NR | Conv | FSWs | 1507 | 0·3 |
| NACP, 1994 [78] | 1993 | National | NR | Conv | FSWs | 2195 | 0·1 |
| NACP, 1994 [78] | 1994 | National | NR | Conv | FSWs | 819 | 0 |
| Shrestha, 1999 [60] | 1994 | NR | NR | NR | FSWs | 2912 | 0·07 |
| Shrestha, 1999 [60] | 1995 | NR | NR | NR | FSWs | 2438 | 0 |
| Jenkins, 2003 [47] | 1999 | NR | NR | NR | FSWs | 205 | 0 |
| Riedner, 2009 [79] | 2008 | NR | NR | NR | FSWs | NR | 0·7 |
| NACP, 2010 [80] | 2008-09 | NR | VCT | Conv | FSWs | 41 | 2·4 |
| **Libya** |  |  |  |  |  |  |  |
| Shazly, 1991 [81] | 1990 | NR | NR | NR | FSWs | 22 | 18·2 |
| Shrestha, 1999 [60] | 1993 | NR | NR | NR | FSWs | 554 | 1·1 |
| Shrestha, 1999 [60] | 1994 | NR | NR | NR | FSWs | 604 | 1·2 |
| **Morocco** |  |  |  |  |  |  |  |
| Benslimane, 1987 [82] | 1984-87 | Casablanca | NR | Conv | FSWs | 27 | 3·7 |
| Riyad, 1990 [83] | 1990 | Casablanca | NR | Conv | FSWs | 28 | 7·1 |
| MOH, 2008 [84] | 2001 | Souss Massa Draa | Sentinel surveillance | Conv | FSWs | NR | 6·3 |
| MOH, 2013 [85] | 2001 | National | Sentinel surveillance | Conv | Incarcerated FSWs | 217 | 2·3 |
| MOH, 2013 [85] | 2002 | National | Sentinel surveillance | Conv | Incarcerated FSWs | 350 | 3·1 |
| MOH, 2006 [86] | 2003 | NR | NGO | Conv | FSWs | 316 | 2·4 |
| MOH, 2013 [85] | 2003 | National | Sentinel surveillance | Conv | Incarcerated FSWs & FSWs attending clinics | 264 | 2·3 |
| MOH, 2013 [85] | 2004 | National | Sentinel surveillance | Conv | Incarcerated FSWs & FSWs attending clinics | 771 | 1·9 |
| Bennani, 2006 [87] | 2005 | NR | Prison | Conv | Incarcerated FSWs | NR | 2·9 |
| MOH, 2008 [84] | 2005 | National | Sentinel surveillance | Conv | FSWs | NR | 2·0 |
| MOH, 2013 [85] | 2005 | National | Sentinel surveillance | Conv | Incarcerated FSWs & FSWs attending clinics | 227 | 2·2 |
| MOH, 2008 [84] | 2006 | Souss Massa Draa | Sentinel surveillance | Conv | FSWs | NR | 4·1 |
| MOH, 2010 [88] | 2006 | National | Sentinel surveillance | Conv | FSWs | NR | 2·5 |
| MOH, 2010 [88] | 2006 | Souss Massa Draa | Sentinel surveillance | Conv | FSWs | NR | 4·3 |
| MOH, 2013 [85] | 2006 | National | Sentinel surveillance | Conv | Incarcerated FSWs & FSWs attending clinics | 650 | 0·2 |
| MOH, 2010 [88] | 2007 | NA | NR | Conv | FSWs | 810 | 2·6 |
| MOH, 2013 [85] | 2007 | National | Sentinel surveillance | Conv | Incarcerated FSWs & FSWs attending clinics | 774 | 2·7 |
| MOH, 2013 [85] | 2008 | National | Sentinel surveillance | Conv | Incarcerated FSWs & FSWs attending clinics | 1079 | 2·1 |
| MOH, 2013 [2] | 2008 | National | VCT | Conv | FSWs | 3110 | 1·3 |
| MOH, 2013 [85] | 2009 | National | Sentinel surveillance | Conv | Incarcerated FSWs & FSWs attending clinics | 965 | 2·4 |
| MOH, 2013 [2] | 2009 | National | VCT | Conv | FSWs | 3484 | 2·1 |
| MOH, 2013 [85] | 2010 | National | Sentinel surveillance | Conv | Incarcerated FSWs & FSWs attending clinics | 1158 | 2·7 |
| MOH, 2013 [2] | 2010 | National | VCT | Conv | FSWs | 4380 | 2·4 |
| MOH, 2013 [89] | 2011 | National | Sentinel surveillance | Conv | FSWs | 1432 | 1·8 |
| MOH, 2013 [2] | 2011 | National | VCT | Conv | FSWs | 4895 | 1·8 |
| Loudyi, 2013[90] | 2012 | Fes | VCT | Conv | FSWs | 927 | 0·9 |
| MOH, 2013 [85] | 2012 | National | Sentinel surveillance | Conv | FSWs attending clinics | 643 | 2·0 |
| MOH, 2013 {Abu-Raddad L, 2010 #43} | 2012 | National | VCT | Conv | FSWs | 10355 | 1·6 |
| **Pakistan** |  |  |  |  |  |  |  |
| Girgis, 1990 [91] | 1986-90 | NR | NR | NR | FSWs | 84 | 0 |
| MENA HIV ESP, 2010 [2] | 1989 | NR | Sentinel surveillance | Conv | FSWs | 84 | 0 |
| MENA HIV ESP, 2010 [2] | 1991-92 | NR | Sentinel surveillance | Conv | FSWs | 17 | 0 |
| Shrestha, 1999 [60] | 1993 | NR | NR | NR | FSWs | 649 | 1·2 |
| Shrestha, 1999 [60] | 1994-95 | NR | NR | NR | FSWs | 142 | 0·7 |
| UNAIDS, 2008 [51] | 1995 | Karachi | NR | NR | FSWs | NR | 0 |
| Shrestha, 1999 [60] | 1996 | NR | NR | NR | FSWs | 104 | 0 |
| Rizvi, 1999 [92] | 1997 | Multan | Red-light district | Conv | FSWs | 577 | 0·5 |
| MENA HIV ESP, 2010 [2] | 1999-00 | NR | Sentinel surveillance | Conv | FSWs | 186 | 3·8 |
| MENA HIV ESP, 2010 [2] | 2001 | NR | Sentinel surveillance | Conv | FSWs | 103 | 0 |
| Shah, 2001 [93] | 2001 | Sindh | VCT | Conv | FSWs | 60 | 1·7 |
| MENA HIV ESP, 2010 [2] | 2002-04 | NR | Sentinel surveillance | Conv | FSWs | 24 | 8·3 |
| Pasha, 2008 [94] | 2007 | Quetta | NR | NR | FSWs | 92 | 0 |
| Riedner, 2009 [79] | 2008 | NR | NR | NR | FSWs | NR | 0·2 |
| Pasha, 2009 [95] | 2009 | Faisalabad | NR | NR | FSWs | 92 | 7 |
| Pasha, 2011 [96] | 2011 | NR | NR | NR | FSWs | NR | 1·2 |
| Mir, 2013 [97] | 2013 | NR | NR | NR | FSWs | NR | 0·6 |
| **Somalia** |  |  |  |  |  |  |  |
| Omar, 1988 [98] | 1986-87 | Mogadishu | Community (urban areas) | Conv | FSWs | 287 | 0·4 |
| Jenkins, 2003 [47] | 1990 | NR | NR | NR | FSWs | NR | 2 |
| Jenkins, 2003 [47] | 1990 | NR | NR | NR | FSWs | NR | 4 |
| Duffy, 1999 [99] | 1999 | Somaliland | NR | NR | FSWs | 17 | 47·1 |
| **Sudan** |  |  |  |  |  |  |  |
| Ahmed, 1990 [100] | 1989 | South Sudan | NR | NR | FSWs | 1027 | 2·8 |
| Ahmed, 1990 [100] | 1989 | East Equatoria, South Sudan | NR | NR | FSWs | 171 | 7·6 |
| Ahmed, 1990 [100] | 1989 | West Equatoria, South Sudan | NR | NR | FSWs | 70 | 24·3 |
| MENA HIV ESP, 2010 [2] | 1989 | NR | Sentinel surveillance | Conv | FSWs | 920 | 2·7 |
| Basha, 2006 [101] | 2006 | NR | NR | NR | FSWs | NR | 1·6 |
| Abu-Raddad, 2010 [2] | 2007 | NR | NR | NR | FSWs | NR | 1·7 |
| Elrashied, 2009 [102] | 2009 | Khartoum | NR | NR | FSWs | 345 | 2·7 |
| NAP, 2015 [28] | 2011 | NR | NR | NR | FSWs | NR | 12 |
| NAP, 2015 [28] | 2014 | NR | NR | NR | FSWs | 764 | 28·9 |
| NAP, 2016 [29] | 2015 | South Sudan | NGO | Conv | FSWs | 2204 | 21 |
| **Syria** |  |  |  |  |  |  |  |
| El-Tayeb, 1995 [103] | 1987-89 | NR | Sentinel surveillance | Conv | FSWs | 294 | 0 |
| El-Tayeb, 1995 [103] | 1990 | NR | Sentinel surveillance | Conv | FSWs | 369 | 0 |
| El-Tayeb, 1995 [103] | 1991 | NR | Sentinel surveillance | Conv | FSWs | 650 | 0 |
| El-Tayeb, 1995 [103] | 1992 | NR | Sentinel surveillance | Conv | FSWs | 502 | 0 |
| El-Tayeb, 1995 [103] | 1992 | NR | Sentinel surveillance | Conv | Bar girls | 1043 | 0 |
| El-Tayeb, 1995 [103] | 1993 | NR | Sentinel surveillance | Conv | FSWs | 794 | 0 |
| El-Tayeb, 1995 [103] | 1993 | NR | Sentinel surveillance | Conv | Bar girls | 697 | 0 |
| El-Tayeb, 1995 [103] | 1994 | NR | Sentinel surveillance | Conv | FSWs | 555 | 0 |
| El-Tayeb, 1995 [103] | 1994 | NR | Sentinel surveillance | Conv | Bar girls | 1825 | 0 |
| Shrestha, 1999 [60] | 1994 | NR | NR | NR | FSWs | 525 | 0 |
| Shrestha, 1999 [60] | 1994 | NR | NR | NR | Bar girls | 1901 | 0 |
| El-Tayeb, 1995 [103] | 1995 | NR | Sentinel surveillance | Conv | FSWs | 59 | 0 |
| El-Tayeb, 1995 [103] | 1995 | NR | Sentinel surveillance | Conv | Bar girls | 158 | 0 |
| Shrestha, 1999 [60] | 1995 | NR | NR | NR | FSWs | 1289 | 0 |
| Shrestha, 1999 [60] | 1995 | NR | NR | NR | Bar girls | 1269 | 0 |
| Shrestha, 1999 [60] | 1996 | NR | NR | NR | FSWs | 1526 | 0 |
| Shrestha, 1999 [60] | 1996 | NR | NR | NR | Bar girls | 1507 | 0 |
| Shrestha, 1999 [60] | 1997 | NR | NR | NR | FSWs | 1707 | 0 |
| Shrestha, 1999 [60] | 1997 | NR | NR | NR | Bar girls | 1717 | 0 |
| Shrestha, 1999 [60] | 1998 | NR | NR | NR | FSWs | 1628 | 0·1 |
| Shrestha, 1999 [60] | 1998 | NR | NR | NR | Bar girls | 2313 | 0·03 |
| MENA HIV ESP, 2010 [2] | 1999 | NR | Sentinel surveillance | Conv | FSWs | 2688 | 0 |
| MENA HIV ESP, 2010 [2] | 1999 | NR | Sentinel surveillance | Conv | Bar girls | 2278 | 0 |
| Shrestha, 1999 [60] | 1999 | NR | NR | NR | FSWs | 1408 | 0 |
| Shrestha, 1999 [60] | 1999 | NR | NR | NR | Bar girls | 1166 | 0 |
| MENA HIV ESP, 2010 [2] | 2000 | NR | Sentinel surveillance | Conv | Bar girls | 2274 | 0 |
| MENA HIV ESP, 2010 [2] | 2000 | NR | Sentinel surveillance | Conv | FSWs | 2188 | 0 |
| MENA HIV ESP, 2010 [2] | 2001 | NR | Sentinel surveillance | Conv | Bar girls | 3304 | 0·1 |
| MENA HIV ESP, 2010 [2] | 2001 | NR | Sentinel surveillance | Conv | FSWs | 2281 | 0 |
| MENA HIV ESP, 2010 [2] | 2002 | NR | Sentinel surveillance | Conv | Bar girls | 2688 | 0·04 |
| MENA HIV ESP, 2010 [2] | 2002 | NR | Sentinel surveillance | Conv | FSWs | 1846 | 0 |
| MENA HIV ESP, 2010 [2] | 2003 | NR | Sentinel surveillance | Conv | Bar girls | 2653 | 0·04 |
| MENA HIV ESP, 2010 [2] | 2003 | NR | Sentinel surveillance | Conv | FSWs | 1019 | 0 |
| MENA HIV ESP, 2010 [2] | 2004 | NR | Sentinel surveillance | Conv | Bar girls | 4784 | 0·02 |
| MENA HIV ESP, 2010 [2] | 2004 | NR | Sentinel surveillance | Conv | FSWs | 1324 | 0 |
| MENA HIV ESP, 2010 [2] | 2005 | NR | Sentinel surveillance | Conv | Bar girls | 2673 | 0 |
| MENA HIV ESP, 2010 [2] | 2005 | NR | Sentinel surveillance | Conv | FSWs | 680 | 0·2 |
| MOH, 2005 [104] | 2005 | Damascus | Sentinel surveillance | Conv | FSWs | 400 | 0·2 |
| MENA HIV ESP, 2010 [2] | 2006, Q1 | National | NR | NR | FSWs | 197 | 0 |
| MENA HIV ESP, 2010 [2] | 2006, Q1 | National | NR | NR | Bar girls | 1528 | 0 |
| MENA HIV ESP, 2010 [2] | 2006, Q2 | National | NR | NR | FSWs | 311 | 0 |
| MENA HIV ESP, 2010 [2] | 2006, Q2 | National | NR | NR | Bar girls | 1354 | 0 |
| MENA HIV ESP, 2010 [2] | 2006, Q3 | National | NR | NR | FSWs | 121 | 0 |
| MENA HIV ESP, 2010 [2] | 2006, Q3 | National | NR | NR | Bar girls | 2001 | 0 |
| MENA HIV ESP, 2010 [2] | 2006, Q4 | National | NR | NR | FSWs | 345 | 0 |
| MENA HIV ESP, 2010 [2] | 2006, Q4 | National | NR | NR | Bar girls | 1197 | 0 |
| MENA HIV ESP, 2010 [2] | 2007, Q2 | National | NR | NR | FSWs | 596 | 0 |
| MENA HIV ESP, 2010 [2] | 2007, Q2 | National | NR | NR | Bar girls | 3570 | 0 |
| MENA HIV ESP, 2010 [2] | 2007, Q3 | National | NR | NR | FSWs | 526 | 0 |
| MENA HIV ESP, 2010 [2] | 2007, Q3 | National | NR | NR | Bar girls | 3421 | 0 |
| NACP, 2008 [105] | 2007 | NR | Sentinel surveillance | Conv | FSWs | 1288 | 0 |
| NACP, 2008 [105] | 2007 | NR | Sentinel surveillance | Conv | Bar girls | 7024 | 0 |
| Al-Sayed, 2010 [106] | 2009 | National | Sentinel surveillance | Conv | FSWs | 878 | 0 |
| Al-Sayed, 2010 [106] | 2009 | National | Sentinel surveillance | Conv | Bar girls | 8479 | 0 |
| MENA HIV ESP, 2013 [46] | 2011 | National | NR | NR | FSWs | 108 | 0 |
| MENA HIV ESP, 2013 [46] | 2011 | National | NR | NR | Bar girls | 6145 | 0 |
| **Tunisia** |  |  |  |  |  |  |  |
| Van de Perre, 1988 [107] | 1985 | NR | NR | NR | FSWs | 108 | 1·9 |
| Giraldo, 1988 [108] | 1985-87 | NR | NR | NR | FSWs | 373 | 1·9 |
| Gharbi, 1987 [109] | 1987 | Tunis | NR | NR | FSWs | 198 | 0 |
| Taibi, 1989 [110] | 1987 | Sfax | NR | NR | FSWs | 36 | 0 |
| MOH, 1990 [111] | 1988-89 | NR | NR | NR | FSWs | 970 | 0·6 |
| MENA HIV ESP, 2010 [2] | 1989 | NR | Sentinel surveillance | Conv | FSWs | 523 | 0 |
| MENA HIV ESP, 2010 [2] | 1989 | NR | Sentinel surveillance | Conv | Bar girls | 447 | 1·3 |
| Fekih, 1991 [112] | 1990 | NR | Sentinel surveillance | Conv | FSWs | 273 | 0 |
| MENA HIV ESP, 2010 [2] | 1991 | NR | Sentinel surveillance | Conv | FSWs | 374 | 0·3 |
| MENA HIV ESP, 2010 [2] | 1992 | NR | Sentinel surveillance | Conv | FSWs | 778 | 0 |
| MENA HIV ESP, 2010 [2] | 1992 | NR | Sentinel surveillance | Conv | Bar girls | 88 | 2·3 |
| NAP, 2005 [113] | 1992 | NR | NR | Conv | Street-based FSWs | NR | 2·3 |
| Shrestha, 1999 [60] | 1993 | NR | NR | NR | FSWs | 402 | 0·3 |
| Shrestha, 1999 [60] | 1994 | NR | NR | NR | FSWs | 880 | 0·1 |
| Shrestha, 1999 [60] | 1995 | NR | NR | NR | FSWs | 1091 | 0 |
| Shrestha, 1999 [60] | 1996 | NR | NR | NR | FSWs | 1020 | 0·4 |
| NAP, 2005 [113] | 1997 | NR | NR | Conv | Street-based FSWs | NR | 0 |
| Shrestha, 1999 [60] | 1997 | NR | NR | NR | FSWs | 992 | 0·1 |
| Shrestha, 1999 [60] | 1998 | NR | NR | NR | FSWs | 694 | 0 |
| MENA HIV ESP, 2010 [2] | 1999 | NR | Sentinel surveillance | Conv | FSWs | 996 | 0 |
| Shrestha, 1999 [60] | 1999 | NR | NR | NR | FSWs | 570 | 0 |
| MENA HIV ESP, 2010 [2] | 2000 | NR | Sentinel surveillance | Conv | FSWs | 483 | 0 |
| NAP, 2005 [113] | 2000 | NR | NR | Conv | FSWs | NR | 0 |
| Jenkins, 2003 [47] | 2001 | NR | NR | NR | FSWs | 458 | 0·2 |
| MENA HIV ESP, 2010 [2] | 2001 | NR | Sentinel surveillance | Conv | FSWs | 554 | 0·2 |
| NAP, 2005 [113] | 2001 | NR | Prison | Conv | Incarcerated FSWs | 100 | 0 |
| MENA HIV ESP, 2010 [2] | 2002 | NR | Sentinel surveillance | Conv | FSWs | 434 | 0 |
| NAP, 2005 [113] | 2002 | NR | NR | Conv | Legal FSWs | 1051 | 0 |
| NAP, 2005 [113] | 2002 | NR | NR | Conv | Street-based FSWs | 125 | 0 |
| MENA HIV ESP, 2010 [2] | 2003 | NR | Sentinel surveillance | Conv | FSWs | 916 | 0 |
| NAP, 2005 [113] | 2003 | NR | NR | Conv | Legal FSWs | 1109 | 0 |
| NAP, 2005 [113] | 2003 | NR | NR | Conv | Street-based FSWs | 13 | 0 |
| MENA HIV ESP, 2010 [2] | 2004 | NR | Sentinel surveillance | Conv | FSWs | 200 | 0 |
| MOH, 2006 [114] | 2004 | NR | NR | Conv | Legal FSWs | 568 | 0 |
| MENA HIV ESP, 2010 [2] | 2005 | NR | Sentinel surveillance | Conv | FSWs | 210 | 0 |
| MOH, 2006 [114] | 2005 | NR | NR | Conv | Legal FSWs | 640 | 0 |
| MOH, 2006 [114] | 2005 | NR | NR | Conv | Street-based FSWs | 18 | 0 |
| MENA HIV ESP, 2010 [2] | 2006, Q2 | National | NR | NR | FSWs & bar girls | 151 | 0 |
| MENA HIV ESP, 2010 [2] | 2006, Q3 | National | NR | NR | FSWs & bar girls | 93 | 0 |
| MENA HIV ESP, 2010 [2] | 2006, Q4 | National | NR | NR | FSWs & bar girls | 213 | 0 |
| MENA HIV ESP, 2010 [2] | 2007, Q1 & Q2 | National | NR | NR | FSWs & bar girls | 83 | 0 |
| UNAIDS, 2008 [115] | 2008 | NR | NR | NR | FSWs | NR | 2·3 |
| MOH, 2010 [116] | 2008 | NR | Sentinel surveillance | Conv | Legal FSWs | 300 | 0·3 |
| MOH, 2010 [116] | 2009 | NR | Sentinel surveillance | Conv | Legal FSWs | NR | 0 |
| **Yemen** |  |  |  |  |  |  |  |
| Shrestha, 1999 [60] | 1998 | NR | NR | NR | FSWs | 88 | 4·6 |
| MENA HIV ESP, 2010 [2] | 1999 | NR | Sentinel surveillance | Conv | FSWs | 73 | 2·7 |
| MENA HIV ESP, 2010 [2] | 2000-01 | NR | Sentinel surveillance | Conv | FSWs | 39 | 0 |
| Jenkins, 2003 [47] | 2001 | NR | NR | NR | FSWs | NR | 7 |
| MENA HIV ESP, 2010 [2] | 2002-03 | NR | Sentinel surveillance | Conv | FSWs | 434 | 0 |
| MENA HIV ESP, 2010 [2] | 2004 | NR | Sentinel surveillance | Conv | FSWs | 203 | 0·5 |
| MENA HIV ESP, 2010 [2] | 2005-06 | NR | Sentinel surveillance | Conv | FSWs | 20 | 0 |
| MENA HIV ESP, 2010 [2] | 2006 Q1, Q2 & Q4 | National | NR | NR | FSWs & bar girls | 20 | 0 |

The table is sorted by year(s) of data collection or year of publication if year of data collection was not reported.

^*^The decimal places of the prevalence figures are as reported in the original reports, but prevalence figures with more than one decimal places were rounded to one decimal place, with the exception of those below 0·1%.

*Abbreviations*: *Conv* convenience, *Dep* department, *FSWs* female sex workers, *MENA HIV ESP* MENA HIV/AIDS Epidemiology Synthesis Project, *MOH* Ministry of Health, *NACP* National AIDS Control programme, *NAP* National AIDS Program, *NGO* non-governmental organization, *NR* not reported, *OMS* Organisation Mondiale de la Sante, *PHC* primary healthcare centers, *Prev* prevalence, *Q* Quarter, *UNAIDS* The Joint United Nations Programme on HIV/AIDS, *VCT* voluntary counselling and testing, *WHO* World Health Organization, *WHO-EMRO* World Health Organization Regional Office for the Eastern Mediterranean

**Table S6** Summary of the risk of bias (ROB) assessment of size estimation and HIV prevalence studies in FSWs and their clients (or proxy populations of clients), in the Middle East and North Africa (MENA). Measures only extracted from routine databases with no reports describing the study methodology were not included in the ROB assessment

| **ROB quality domains** | **Size estimation studies** | | | | **HIV prevalence studies** | | | |
| --- | --- | --- | --- | --- | --- | --- | --- | --- |
|  | **FSWs** | | **Clients** | | **FSWs** | | **Clients** | |
|  | **n** | **%** | **n** | **%** | **n** | **%** | **n** | **%** |
| **Sex work definition** |  |  |  |  |  |  |  |  |
| Low ROB | 153 | 95·0 | 39 | 100·0 | 116 | 78.9 | 12 | 36·4 |
| High ROB | 0 | 0·0 | 0 | 0·0 | 0 | 0.0 | 1 | 3·0 |
| Unclear | 8 | 5·0 | 0 | 0·0 | 31 | 21.1 | 20 | 60·6 |
|  |  |  |  |  |  |  |  |  |
| **Estimation methodology** |  |  |  |  |  |  |  |  |
| Low ROB | 156 | 96·9 | 27 | 69·2 | NA | NA | NA | NA |
| High ROB | 5 | 3·1 | 12 | 30·8 | NA | NA | NA | NA |
| Unclear | 0 | 0·0 | 0 | 0·0 | NA | NA | NA | NA |
|  |  |  |  |  |  |  |  |  |
| **Rigor of sampling methodology** |  |  |  |  |  |  |  |  |
| Low ROB | NA | NA | NA | NA | 101 | 68.7 | 4 | 12·1 |
| High ROB | NA | NA | NA | NA | 43 | 29.3 | 29 | 87·9 |
| Unclear | NA | NA | NA | NA | 3 | 2.0 | 0 | 0·0 |
|  |  |  |  |  |  |  |  |  |
| **Response rate** |  |  |  |  |  |  |  |  |
| Low ROB | 86 | 53·4 | 19 | 48·7 | 92 | 62.6 | 4 | 12·1 |
| High ROB | 4 | 2·5 | 1 | 2·5 | 8 | 5.4 | 1 | 3·0 |
| Unclear | 71 | 44·1 | 19 | 48·7 | 47 | 32.0 | 28 | 84·9 |
|  |  |  |  |  |  |  |  |  |
| **HIV ascertainment** |  |  |  |  |  |  |  |  |
| Low ROB | NA | NA | NA | NA | 146 | 99.3 | 33 | 100·0 |
| High ROB | NA | NA | NA | NA | 1 | 0.7 | 0 | 0·0 |
| Unclear | NA | NA | NA | NA | 0 | 0.0 | 0 | 0·0 |
|  |  |  |  |  |  |  |  |  |
| **Total number of studies** | 161 | 100·0 | 39 | 100·0 | 147 | 100.0 | 33 | 100·0 |
|  |  |  |  |  |  |  |  |  |
| **Summary** |  |  |  |  |  |  |  |  |
| **Low ROB** |  |  |  |  |  |  |  |  |
| At least 1 domain | 161 | 100·0 | 39 | 100·0 | 147 | 100.0 | 33 | 100·0 |
| At least 2 domains | 152 | 94·4 | 32 | 82·1 | 125 | 85.0 | 13 | 39·4 |
| At least 3 domains | 82 | 50·9 | 14 | 35·9 | 79 | 53.7 | 2 | 6·1 |
|  |  |  |  |  |  |  |  |  |
| **High ROB** |  |  |  |  |  |  |  |  |
| At least 1 domain | 9 | 5·6 | 13 | 33·3 | 51 | 34.7 | 29 | 87·9 |
| At least 2 domains | 0 | 0·0 | 0 | 0·0 | 1 | 0.7 | 2 | 6·1 |
| At least 3 domains | 0 | 0·0 | 0 | 0·0 | 0 | 0.0 | 0 | 0·0 |

*Abbreviations*: *FSWs* female sex workers, *NA* not applicable

**Table S7** Risk of bias (ROB) assessment of estimates of national and subnational representation for the number and population proportion of FSWs and of their clients, in the Middle East and North Africa

| **Country**  **Author, year [citation]** | **Year(s) of data collection** | **Size estimate** | | **Risk of bias assessment** | | |
| --- | --- | --- | --- | --- | --- | --- |
|  |  | **N or range** | **%** | **Sex work definition** | **Estimation methodology** | **Response rate** |
| **FSWs** |  |  |  |  |  |  |
| ***National estimates*** |  |  |  |  |  |  |
| **Egypt** |  |  |  |  |  |  |
| Bahaa, 2010 [117] | 2004-08 | NR | 0·4 | Low ROB | High ROB | Unclear |
| Jacobsen, 2014 [8] | 2014 | 22,986 | 0·24 | Low ROB | Low ROB | Unclear |
| **Iran** |  |  |  |  |  |  |
| Sharifi, 2017 [10] | 2015 | 19,800 | 0·31 | Low ROB | Low ROB | Unclear |
| Sharifi, 2017 [10] | 2015 | 98,500 | 1·54 | Low ROB | Low ROB | Unclear |
| Sharifi, 2017 [10] | 2015 | 152,200 | 2·38 | Low ROB | Low ROB | Unclear |
| **Lebanon** |  |  |  |  |  |  |
| Kahhaleh, 2009 [118] | 1996 | NR | 0·54 | Low ROB | Low ROB | Unclear |
| Kahhaleh, 2009 [118] | 2004 | NR | 0·53 | Low ROB | Low ROB | Low ROB |
| **Morocco** |  |  |  |  |  |  |
| Bennani, 2013 [119] | 2011 | 85,000 | NR | Low ROB | Low ROB | Unclear |
| MOH, 2013 [120] | 2013 | NR | 6·9 | Low ROB | Low ROB | Low ROB |
| MOH, 2013 [120] | 2013 | NR | 2·4 | Low ROB | Low ROB | Low ROB |
| **Pakistan** |  |  |  |  |  |  |
| NACP, 2005 [15] (round I) | 2005 | 35,050 | 0·78 | Low ROB | Low ROB | Low ROB |
| Emmanuel, 2010 [16] (round II) | 2006 | 167,501 | 0·44 | Low ROB | Low ROB | Low ROB |
| Emmanuel, 2013 [19, 20] (round IV) | 2011-12 | 89,178 | 0·72 | Low ROB | Low ROB | Low ROB |
| NACP, 2017 [22] (round V) | 2016-17 | 64,829 | NR | Low ROB | Low ROB | Low ROB |
| **Sudan** |  |  |  |  |  |  |
| AFROCENTER Group, 2005 [121] | 2005 | NR | 0·4 | Low ROB | High ROB | Unclear |
| **Yemen** |  |  |  |  |  |  |
| MOH, 2010 [32] | NR | 58,934 | 1·16-2·10 | Unclear | Low ROB | Unclear |
| ***Subnational estimates*** |  |  |  |  |  |  |
| **Afghanistan** |  |  |  |  |  |  |
| SAR AIDS HDS, 2008 [5] | 2006-07 | 90 | 0·26 | Low ROB | Low ROB | Unclear |
| SAR AIDS HDS, 2008 [5] | 2006-07 | 898 | 0·19 | Low ROB | Low ROB | Unclear |
| SAR AIDS HDS, 2008 [5] | 2006-07 | 172 | 0·28 | Low ROB | Low ROB | Unclear |
| NACP, 2012 [6] (round II) | 2012 | 2,134 | NR | Low ROB | Low ROB | Low ROB |
| NACP, 2012 [6] (round II) | 2012 | 2,800 | NR | Low ROB | Low ROB | Low ROB |
| **Djibouti** |  |  |  |  |  |  |
| Trellu-Kane, 2005 [7] | 2005 | NR | 4 | Low ROB | High ROB | Low ROB |
| **Egypt** |  |  |  |  |  |  |
| Jacobsen, 2014 [8] | 2014 | 6,092 | 0·17 | Low ROB | Low ROB | Unclear |
| Jacobsen, 2014 [8] | 2014 | 4,225 | 0·34 | Low ROB | Low ROB | Unclear |
| Jacobsen, 2014 [8] | 2014 | 1,345 | 0·34 | Low ROB | Low ROB | Unclear |
| Jacobsen, 2014 [8] | 2014 | 1,315 | 1·92 | Low ROB | Low ROB | Unclear |
| Jacobsen, 2014 [8] | 2014 | 278 | 0·11 | Low ROB | Low ROB | Unclear |
| **Iran** |  |  |  |  |  |  |
| Karami, 2017 [9] | NR | 842 | 0·45 | Low ROB | Low ROB | Low ROB |
| Sharifi, 2017 [10] | 2015 | 10,000 | 2·86 | Low ROB | Low ROB | Unclear |
| Sharifi, 2017 [10] | 2015 | 3,800 | 2·30 | Low ROB | Low ROB | Unclear |
| Sharifi, 2017 [10] | 2015 | 4,000 | 2·87 | Low ROB | Low ROB | Unclear |
| Sharifi, 2017 [10] | 2015 | 12,200 | 2·02 | Low ROB | Low ROB | Unclear |
| Sharifi, 2017 [10] | 2015 | 4,600 | 2·46 | Low ROB | Low ROB | Unclear |
| Sharifi, 2017 [10] | 2015 | 1,600 | 0·59 | Low ROB | Low ROB | Unclear |
| Sharifi, 2017 [10] | 2015 | 12,000 | 1·43 | Low ROB | Low ROB | Unclear |
| Sharifi, 2017 [10] | 2015 | 800 | 0·85 | Low ROB | Low ROB | Unclear |
| Sharifi, 2017 [10] | 2015 | 13,300 | 2·75 | Low ROB | Low ROB | Unclear |
| Sharifi, 2017 [10] | 2015 | 13,100 | 2·84 | Low ROB | Low ROB | Unclear |
| Sharifi, 2017 [10] | 2015 | 63,700 | 2·52 | Low ROB | Low ROB | Unclear |
| Sharifi, 2017 [10] | 2015 | 840 | 0·51 | Low ROB | Low ROB | Unclear |
| Sharifi, 2017 [10] | 2015 | 1,200 | 0·35 | Low ROB | Low ROB | Unclear |
| Sharifi, 2017 [10] | 2015 | 3,000 | 1·81 | Low ROB | Low ROB | Unclear |
| Sharifi, 2017 [10] | 2015 | 390 | 0·28 | Low ROB | Low ROB | Unclear |
| Sharifi, 2017 [10] | 2015 | 2,300 | 0·38 | Low ROB | Low ROB | Unclear |
| Sharifi, 2017 [10] | 2015 | 1,400 | 0·73 | Low ROB | Low ROB | Unclear |
| Sharifi, 2017 [10] | 2015 | 70 | 0·03 | Low ROB | Low ROB | Unclear |
| Sharifi, 2017 [10] | 2015 | 200 | 0·17 | Low ROB | Low ROB | Unclear |
| Sharifi, 2017 [10] | 2015 | 3,000 | 0·35 | Low ROB | Low ROB | Unclear |
| Sharifi, 2017 [10] | 2015 | 4,700 | 5 | Low ROB | Low ROB | Unclear |
| Sharifi, 2017 [10] | 2015 | 1,300 | 0·26 | Low ROB | Low ROB | Unclear |
| Sharifi, 2017 [10] | 2015 | 170 | 0·04 | Low ROB | Low ROB | Unclear |
| Sharifi, 2017 [10] | 2015 | 7,500 | 0·3 | Low ROB | Low ROB | Unclear |
| Sharifi, 2017 [10] | 2015 | 4,300 | 1·22 | Low ROB | Low ROB | Unclear |
| Sharifi, 2017 [10] | 2015 | 2,200 | 1·30 | Low ROB | Low ROB | Unclear |
| Sharifi, 2017 [10] | 2015 | 2,200 | 1·56 | Low ROB | Low ROB | Unclear |
| Sharifi, 2017 [10] | 2015 | 14,700 | 2·44 | Low ROB | Low ROB | Unclear |
| Sharifi, 2017 [10] | 2015 | 2,000 | 1·06 | Low ROB | Low ROB | Unclear |
| Sharifi, 2017 [10] | 2015 | 4,000 | 1·47 | Low ROB | Low ROB | Unclear |
| Sharifi, 2017 [10] | 2015 | 740 | 0·65 | Low ROB | Low ROB | Unclear |
| Sharifi, 2017 [10] | 2015 | 15,200 | 1·81 | Low ROB | Low ROB | Unclear |
| Sharifi, 2017 [10] | 2015 | 1,500 | 1·54 | Low ROB | Low ROB | Unclear |
| Sharifi, 2017 [10] | 2015 | 8,100 | 1·67 | Low ROB | Low ROB | Unclear |
| Sharifi, 2017 [10] | 2015 | 640 | 0·14 | Low ROB | Low ROB | Unclear |
| Sharifi, 2017 [10] | 2015 | 38,700 | 1·54 | Low ROB | Low ROB | Unclear |
| Sharifi, 2017 [10] | 2015 | 2,600 | 1·63 | Low ROB | Low ROB | Unclear |
| Karami, 2017 [11] | 2016 | 690 | NR | Low ROB | Low ROB | Low ROB |
| **Morocco** |  |  |  |  |  |  |
| MOH, 2012 [12] | 2011-12 | 3,639-4,333 | NR | Low ROB | Low ROB | Low ROB |
| MOH, 2012 [12] | 2011-12 | 6,028 | NR | Low ROB | Low ROB | Low ROB |
| MOH, 2012 [12] | 2011-12 | 5,683 | NR | Low ROB | Low ROB | Low ROB |
| MOH, 2012 [12] | 2011-12 | 3,956 | NR | Low ROB | Low ROB | Low ROB |
| Huygens, 2013 [13] | 2013 | 955 | NR | Unclear | Low ROB | Low ROB |
| Huygens, 2013 [13] | 2013 | 7,253 | NR | Unclear | Low ROB | Low ROB |
| **Pakistan** |  |  |  |  |  |  |
| NACP, 2005 [14] (pilot) | 2004-05 | 11,546 | NR | Low ROB | Low ROB | Low ROB |
| NACP, 2005 [14] (pilot) | 2004-05 | 1,596 | NR | Low ROB | Low ROB | Low ROB |
| NACP, 2005 [15] (round I) | 2005 | 2,050 | 0·46 | Low ROB | Low ROB | Low ROB |
| NACP, 2005 [15] (round I) | 2005 | 1,350 | 0·69 | Low ROB | Low ROB | Low ROB |
| NACP, 2005 [15] (round I) | 2005 | 11,550 | 0·58 | Low ROB | Low ROB | Low ROB |
| NACP, 2005 [15] (round I) | 2005 | 14,150 | 1·26 | Low ROB | Low ROB | Low ROB |
| NACP, 2005 [15] (round I) | 2005 | 2,500 | 0·99 | Low ROB | Low ROB | Low ROB |
| NACP, 2005 [15] (round I) | 2005 | 950 | 0·45 | Low ROB | Low ROB | Low ROB |
| NACP, 2005 [15] (round I) | 2005 | 750 | 0·64 | Low ROB | Low ROB | Low ROB |
| NACP, 2005 [15] (round I) | 2005 | 1,750 | 0·88 | Low ROB | Low ROB | Low ROB |
| Emmanuel, 2010 [16] (round II) | 2006 | 125 | 0·04 | Low ROB | Low ROB | Low ROB |
| Emmanuel, 2010 [16] (round II) | 2006 | 9,500 | 1·30 | Low ROB | Low ROB | Low ROB |
| Emmanuel, 2010 [16] (round II) | 2006 | 2,421 | 0·58 | Low ROB | Low ROB | Low ROB |
| Emmanuel, 2010 [16] (round II) | 2006 | 2,750 | 0·71 | Low ROB | Low ROB | Low ROB |
| Emmanuel, 2010 [16] (round II) | 2006 | 25,550 | 0·74 | Low ROB | Low ROB | Low ROB |
| Emmanuel, 2010 [16] (round II) | 2006 | 24,625 | 1·34 | Low ROB | Low ROB | Low ROB |
| Emmanuel, 2010 [16] (round II) | 2006 | 525 | 0·44 | Low ROB | Low ROB | Low ROB |
| Emmanuel, 2010 [16] (round II) | 2006 | 5,075 | 1·22 | Low ROB | Low ROB | Low ROB |
| Emmanuel, 2010 [16] (round II) | 2006 | 1,550 | 0·44 | Low ROB | Low ROB | Low ROB |
| Emmanuel, 2010 [16] (round II) | 2006 | 2,500 | 1·10 | Low ROB | Low ROB | Low ROB |
| Emmanuel, 2010 [16] (round II) | 2006 | 1,596 | 0·31 | Low ROB | Low ROB | Low ROB |
| Emmanuel, 2010 [16] (round II) | 2006 | 1,831 | 0·67 | Low ROB | Low ROB | Low ROB |
| Emmanuel, 2010 [16] (round II) | 2006 | 2,550 | 1·14 | Low ROB | Low ROB | Low ROB |
| Khan, 2011 [17] | 2007 | 5,226 | NR | Low ROB | Low ROB | Low ROB |
| Khan, 2011 [17] | 2007 | NR | 0·43 | Low ROB | Low ROB | Low ROB |
| Khan, 2011 [17] | 2007 | NR | 0·56 | Low ROB | Low ROB | Low ROB |
| NACP, 2008 [18] | 2007 | 86 | NR | Low ROB | Low ROB | Unclear |
| NACP, 2008 [18] | 2007 | 498 | NR | Low ROB | Low ROB | Unclear |
| NACP, 2008 [18] | 2007 | 9 | NR | Low ROB | Low ROB | Unclear |
| NACP, 2008 [18] | 2007 | 5 | NR | Low ROB | Low ROB | Unclear |
| NACP, 2008 [18] | 2007 | 2 | NR | Low ROB | Low ROB | Unclear |
| NACP, 2008 [18] | 2007 | 1,030 | NR | Low ROB | Low ROB | Unclear |
| NACP, 2008 [18] | 2007 | 105 | NR | Low ROB | Low ROB | Unclear |
| Emmanuel, 2013 [19, 20] (round IV) | 2011-12 | 1,413 | 1·30 | Low ROB | Low ROB | Low ROB |
| Emmanuel, 2013 [19, 20] (round IV) | 2011-12 | 4,846 | 0·50 | Low ROB | Low ROB | Low ROB |
| Emmanuel, 2013 [19, 20] (round IV) | 2011-12 | 2,994 | 1·19 | Low ROB | Low ROB | High ROB |
| Emmanuel, 2013 [19, 20] (round IV) | 2011-12 | 4,566 | 0·85 | Low ROB | Low ROB | Low ROB |
| Emmanuel, 2013 [19, 20] (round IV) | 2011-12 | 25,399 | 0·55 | Low ROB | Low ROB | Low ROB |
| Emmanuel, 2013 [19, 20] (round IV) | 2011-12 | 23,766 | 1·15 | Low ROB | Low ROB | Low ROB |
| Emmanuel, 2013 [19, 20] (round IV) | 2011-12 | 1,114 | 0·82 | Low ROB | Low ROB | Low ROB |
| Emmanuel, 2013 [19, 20] (round IV) | 2011-12 | 884 | 0·85 | Low ROB | Low ROB | Low ROB |
| Emmanuel, 2013 [19, 20] (round IV) | 2011-12 | 5,308 | 0·80 | Low ROB | Low ROB | Low ROB |
| Emmanuel, 2013 [19, 20] (round IV) | 2011-12 | 2,011 | 1·42 | Low ROB | Low ROB | Low ROB |
| Emmanuel, 2013 [19, 20] (round IV) | 2011-12 | 3,317 | 0·42 | Low ROB | Low ROB | Low ROB |
| Emmanuel, 2013 [19, 20] (round IV) | 2011-12 | 3,710 | 1·07 | Low ROB | Low ROB | Low ROB |
| Emmanuel, 2013 [19, 20] (round IV) | 2011-12 | 3,635 | 0·34 | Low ROB | Low ROB | Low ROB |
| Emmanuel, 2013 [19, 20] (round IV) | 2011-12 | 3,898 | 1·25 | Low ROB | Low ROB | Low ROB |
| Emmanuel, 2013 [19, 20] (round IV) | 2011-12 | 2,317 | 1·05 | Low ROB | Low ROB | Low ROB |
| Punjab ACP, 2015 [21] | 2014 | 7,556 | NR | Low ROB | Low ROB | Low ROB |
| Punjab ACP, 2015 [21] | 2014 | 25,716 | NR | Low ROB | Low ROB | Low ROB |
| Punjab ACP, 2015 [21] | 2014 | 6,561 | NR | Low ROB | Low ROB | Low ROB |
| Punjab ACP, 2015 [21] | 2014 | 4,327 | NR | Low ROB | Low ROB | Low ROB |
| NACP, 2017 [22] (round V) | 2016-17 | 6,201 | NR | Low ROB | Low ROB | Low ROB |
| NACP, 2017 [22] (round V) | 2016-17 | 192 | NR | Low ROB | Low ROB | High ROB |
| NACP, 2017 [22] (round V) | 2016-17 | 1,349 | NR | Low ROB | Low ROB | Low ROB |
| NACP, 2017 [22] (round V) | 2016-17 | 4,069 | NR | Low ROB | Low ROB | Low ROB |
| NACP, 2017 [22] (round V) | 2016-17 | 317 | NR | Low ROB | Low ROB | Low ROB |
| NACP, 2017 [22] (round V) | 2016-17 | 4,426 | NR | Low ROB | Low ROB | Low ROB |
| NACP, 2017 [22] (round V) | 2016-17 | 25,191 | NR | Low ROB | Low ROB | Low ROB |
| NACP, 2017 [22] (round V) | 2016-17 | 1,739 | NR | Low ROB | Low ROB | Low ROB |
| NACP, 2017 [22] (round V) | 2016-17 | 4,593 | NR | Low ROB | Low ROB | Low ROB |
| NACP, 2017 [22] (round V) | 2016-17 | 2,084 | NR | Low ROB | Low ROB | Low ROB |
| NACP, 2017 [22] (round V) | 2016-17 | 1,690 | NR | Low ROB | Low ROB | Low ROB |
| NACP, 2017 [22] (round V) | 2016-17 | 765 | NR | Low ROB | Low ROB | Low ROB |
| NACP, 2017 [22] (round V) | 2016-17 | 2,465 | NR | Low ROB | Low ROB | Low ROB |
| NACP, 2017 [22] (round V) | 2016-17 | 4,121 | NR | Low ROB | Low ROB | Low ROB |
| NACP, 2017 [22] (round V) | 2016-17 | 6,252 | NR | Low ROB | Low ROB | Low ROB |
| NACP, 2017 [22] (round V) | 2016-17 | 2,031 | NR | Low ROB | Low ROB | High ROB |
| NACP, 2017 [22] (round V) | 2016-17 | 3,307 | NR | Low ROB | Low ROB | Low ROB |
| NACP, 2017 [22] (round V) | 2016-17 | 523 | NR | Low ROB | Low ROB | High ROB |
| **Somalia** |  |  |  |  |  |  |
| MOH, 2016 [24] | 2016 | 911 | NR | Low ROB | Low ROB | Unclear |
| MOH, 2016 [24] | 2016 | 1,126 | NR | Low ROB | Low ROB | Unclear |
| MOH, 2016 [24] | 2016 | 963 | NR | Low ROB | Low ROB | Unclear |
| **Sudan** |  |  |  |  |  |  |
| NACP, 2002 [25] | 2002 | NR | 0·83 | Low ROB | Low ROB | Low ROB |
| NACP, 2002 [25] | 2002 | NR | 0·5 | Low ROB | High ROB | Low ROB |
| NACP, 2005 [26] | 2005 | NR | 3 | Low ROB | High ROB | Low ROB |
| UNHCR, 2007 [27] | 2006 | NR | 0·4 | Low ROB | Low ROB | Low ROB |
| UNHCR, 2007 [27] | 2006 | NR | 0·2 | Low ROB | Low ROB | Low ROB |
| MOH, 2016 [30] | 2015-16 | 5,800 | NR | Low ROB | Low ROB | Low ROB |
| MOH, 2016 [30] | 2015-16 | 5,306 | NR | Low ROB | Low ROB | Low ROB |
| **Tunisia** |  |  |  |  |  |  |
| Hsairi, 2012 [31] | 2011 | 541 | NR | Low ROB | Low ROB | Low ROB |
| Hsairi, 2012 [31] | 2011 | 596 | NR | Low ROB | Low ROB | Low ROB |
| Hsairi, 2012 [31] | 2011 | 291 | NR | Low ROB | Low ROB | Low ROB |
| **Yemen** |  |  |  |  |  |  |
| MOH, 2010 [32] | NR | 1,875-4,260 | 1·16-2·64 | Unclear | Low ROB | Unclear |
| MOH, 2010 [32] | NR | 1,580-1,759 | 1·89-2·10 | Unclear | Low ROB | Unclear |
| MOH, 2010 [32] | NR | 1,488-1,786 | 2·07-2·49 | Unclear | Low ROB | Unclear |
| MOH, 2010 [32] | NR | 3,092-4,495 | 0·64-2·10 | Unclear | Low ROB | Unclear |
| MOH, 2010 [32] | NR | 1,050-1,835 | 0·80-1·40 | Unclear | Low ROB | Unclear |
| **Clients of FSWs** |  |  |  |  |  |  |
| ***National estimates*** |  |  |  |  |  |  |
| **Afghanistan** |  |  |  |  |  |  |
| Todd, 2007 [122] | 2005-06 | NR | 3·57 | Low ROB | Low ROB | Unclear |
| Todd, 2012 [123] | 2010-11 | NR | 12·5 | Low ROB | Low ROB | Low ROB |
| **Egypt** |  |  |  |  |  |  |
| Bahaa, 2010 [117] | 2004-08 | NR | 0·9 | Low ROB | High ROB | Unclear |
| **Lebanon** |  |  |  |  |  |  |
| Kahhaleh, 2009 [118] | 1996 | NR | 9·7 | Low ROB | Low ROB | Unclear |
| Adib, 2002 [124] | 1999 | NR | 13·84 | Low ROB | Low ROB | Low ROB |
| Kahhaleh, 2009 [118] | 2004 | NR | 5·65 | Low ROB | Low ROB | Low ROB |
| **Morocco** |  |  |  |  |  |  |
| MOH, 2007 [125] | 2007 | NR | 35·3 | Low ROB | Low ROB | Unclear |
| MOH, 2007 [125] | 2007 | NR | 2 | Low ROB | Low ROB | Unclear |
| MOH, 2013 [120] | 2013 | NR | 10·5 | Low ROB | Low ROB | Low ROB |
| MOH, 2013 [120] | 2013 | NR | 0·3 | Low ROB | Low ROB | Low ROB |
| **Pakistan** |  |  |  |  |  |  |
| Mir, 2013 [126] | 2007 | NR | 11·9 | Low ROB | Low ROB | Low ROB |
| Mir, 2013 [126] | 2007 | NR | 5·8 | Low ROB | Low ROB | Low ROB |
| **Sudan** |  |  |  |  |  |  |
| NACP, 2004 [127] | 2004 | NR | 0·3 | Low ROB | High ROB | Unclear |
| AFROCENTER Group, 2005 [121] | 2005 | NR | 0·5 | Low ROB | High ROB | Unclear |
| ***Subnational estimates*** |  |  |  |  |  |  |
| **Afghanistan** |  |  |  |  |  |  |
| Mansoor, 2008 [33] | 2007 | NR | 5·2 | Low ROB | Low ROB | Low ROB |
| **Djibouti** |  |  |  |  |  |  |
| Trellu-Kane, 2005 [7] | 2005 | NR | 17 | Low ROB | High ROB | Low ROB |
| **Iran** |  |  |  |  |  |  |
| Shokoohi, 2012 [34] | NR | 9,314 | 7·0 | Low ROB | Low ROB | Unclear |
| Shokoohi, 2012 [34] | NR | 3,203 | 2·4 | Low ROB | Low ROB | Unclear |
| Khalajabadi, 2018 [35] | 2013-14 | NR | 1·3 | Low ROB | Low ROB | Low ROB |
| Khalajabadi, 2018 [35] | 2013-14 | NR | 6·6 | Low ROB | Low ROB | Low ROB |
| **Lebanon** |  |  |  |  |  |  |
| Melikian, 1954 [36] | 1952 | NR | 59·3 | Low ROB | High ROB | Unclear |
| Melikian, 1967 [37] | 1963 | NR | 40·6 | Low ROB | High ROB | Low ROB |
| Ghandour, 2014 [38] | 2012 | NR | 20·1 | Low ROB | Low ROB | High ROB |
| **Pakistan** |  |  |  |  |  |  |
| Faisel, 2005 [39] | 2004-05 | NR | 6·8 | Low ROB | Low ROB | Low ROB |
| Minhas, 2005 [40] | 2005 | NR | 7 | Low ROB | High ROB | Unclear |
| **Somalia** |  |  |  |  |  |  |
| Ismail, 1990 [41] | 1986 | NR | 48 | Low ROB | High ROB | Unclear |
| Ismail, 1990 [42] | 1987 | NR | 29 | Low ROB | Low ROB | Low ROB |
| MOH, 2016 [24] | 2016 | 3,469 | NR | Low ROB | Low ROB | Unclear |
| MOH, 2016 [24] | 2016 | 3,530 | NR | Low ROB | Low ROB | Unclear |
| MOH, 2016 [24] | 2016 | 1,828 | NR | Low ROB | Low ROB | Unclear |
| MOH, 2016 [24] | 2016 | 1,559 | NR | Low ROB | Low ROB | Unclear |
| MOH, 2016 [24] | 2016 | 2,599 | NR | Low ROB | Low ROB | Unclear |
| MOH, 2016 [24] | 2016 | 2,202 | NR | Low ROB | Low ROB | Unclear |
| **Sudan** |  |  |  |  |  |  |
| McCarthy, 1989 [43] | 1987-88 | NR | 51·6 | Low ROB | High ROB | Unclear |
| Holt, 2003 [44] | 1992 | NR | 46·0 | Low ROB | High ROB | Low ROB |
| Holt, 2003 [44] | 1992 | NR | 31·0 | Low ROB | High ROB | Low ROB |
| NACP, 2002 [25] | 2002 | NR | 11·7 | Low ROB | High ROB | Low ROB |
| UNHCR, 2007 [27] | 2006 | NR | 1·7 | Low ROB | Low ROB | Low ROB |
| UNHCR, 2007 [27] | 2006 | NR | 1·4 | Low ROB | Low ROB | Low ROB |

The table is sorted by year(s) of data collection or year of publication if year of data collection was not reported.

*Abbreviations*: *ACP* AIDS Control Program, *FSWs* female sex workers, *MOH* Ministry of Health, *NACP* National AIDS Control Programme, *NAP* National AIDS Program, *NR* not reported, *SAR AIDS HDS* South Asia Region AIDS Human Development Sector, *UNHCR* United Nations Higher Commission for Refugees

**Table S8** Risk of bias (ROB) assessment of HIV prevalence studies in FSWs in the Middle East and North Africa

| **Country**  **Author, year [citation]** | **Year(s) of data collection** | **Sample size** | **HIV prev** **(%)** | **Sex work definition** | **Sampling methodology** | **Response rate** | **HIV ascertainment** |
| --- | --- | --- | --- | --- | --- | --- | --- |
| **Studies using probability-based sampling** | | | | | | | |
| **Afghanistan** |  |  |  |  |  |  |  |
| SAR AIDS HDS, 2008 [5] | 2006-07 | 45 | 0 | Low ROB | Low ROB | Unclear | Low ROB |
| SAR AIDS HDS, 2008 [5] | 2006-07 | 87 | 0 | Low ROB | Low ROB | Unclear | Low ROB |
| NACP, 2010 [128] (round I) | 2009 | 368 | 0 | Low ROB | Low ROB | Low ROB | Low ROB |
| NACP, 2012 [6] (round II) | 2012 | 344 | 0·9 | Low ROB | Low ROB | Low ROB | Low ROB |
| NACP, 2012 [6] (round II) | 2012 | 333 | 0 | Low ROB | Low ROB | Low ROB | Low ROB |
| NACP, 2012 [6] (round II) | 2012 | 355 | 0 | Low ROB | Low ROB | Low ROB | Low ROB |
| **Egypt** |  |  |  |  |  |  |  |
| MOH, 2006 [129] (round I) | 2006 | 118 | 0·8 | Unclear | High ROB | High ROB | Low ROB |
| MOH, 2010 [130] (round II) | 2010 | 200 | 0 | Low ROB | High ROB | Low ROB | Low ROB |
| **Iran** |  |  |  |  |  |  |  |
| Navadeh, 2012 [131] | 2010 | 139 | 0 | Low ROB | Low ROB | Low ROB | Low ROB |
| Sajadi, 2013 [132] (round I) | 2010 | 817 | 4·5 | Low ROB | Low ROB | Low ROB | Low ROB |
| Kazerooni, 2014 [133] | 2010-11 | 278 | 4·7 | Low ROB | Low ROB | Low ROB | Low ROB |
| Moaeyedi-Nia [134] | 2012-13 | 161 | 5 | Low ROB | Low ROB | Unclear | Low ROB |
| Mirzazadeh, 2016 [135] (round II) | 2015 | 1,337 | 2·1 | Low ROB | High ROB | Unclear | Low ROB |
| Karami, 2017 [11] | 2016 | 369 | 4·6 | Low ROB | Low ROB | Low ROB | High ROB |
| **Jordan** |  |  |  |  |  |  |  |
| WHO, 2011 [23] (round I) | 2009 | 225 | 0 | Unclear | Low ROB | Unclear | Low ROB |
| MOH, 2014 [136] (round II) | 2013 | 358 | 0·6 | Low ROB | Low ROB | Unclear | Low ROB |
| MOH, 2014 [136] (round II) | 2013 | 102 | 0 | Low ROB | Low ROB | Unclear | Low ROB |
| MOH, 2014 [136] (round II) | 2013 | 212 | 0·5 | Low ROB | Low ROB | Unclear | Low ROB |
| **Lebanon** |  |  |  |  |  |  |  |
| Mahfoud, 2010 [137] | 2007-08 | 95 | 0 | Low ROB | Low ROB | High ROB | Low ROB |
| **Libya** |  |  |  |  |  |  |  |
| Valadez, 2013 [138] (round I) | 2010-11 | 69 | 15·7 | Low ROB | Low ROB | High ROB | Low ROB |
| **Morocco** |  |  |  |  |  |  |  |
| MOH, 2012 [12] | 2011-12 | 364 | 5·1 | Low ROB | Low ROB | Low ROB | Low ROB |
| MOH, 2012 [12] | 2011-12 | 359 | 1·8 | Low ROB | Low ROB | Low ROB | Low ROB |
| MOH, 2012 [12] | 2011-12 | 392 | 0 | Low ROB | Low ROB | Low ROB | Low ROB |
| MOH, 2012 [12] | 2011-12 | 319 | 1·4 | Low ROB | Low ROB | Low ROB | Low ROB |
| **Pakistan** |  |  |  |  |  |  |  |
| Bokhari, 2007 [139] | 2004 | 378 | 0·5 | Low ROB | Low ROB | Low ROB | Low ROB |
| NACP, 2005 [15] (round I) | 2005 | 400 | 0 | Low ROB | Low ROB | Low ROB | Low ROB |
| NACP, 2005 [15] (round I) | 2005 | 400 | 0 | Low ROB | Low ROB | Low ROB | Low ROB |
| NACP, 2005 [15] (round I) | 2005 | 400 | 0·8 | Low ROB | Low ROB | Low ROB | Low ROB |
| NACP, 2005 [15] (round I) | 2005 | 400 | 0 | Low ROB | Low ROB | Low ROB | Low ROB |
| NACP, 2005 [15] (round I) | 2005 | 400 | 0 | Low ROB | Low ROB | Low ROB | Low ROB |
| NACP, 2005 [15] (round I) | 2005 | 359 | 0 | Low ROB | Low ROB | Low ROB | Low ROB |
| NACP, 2005 [15] (round I) | 2005 | 411 | 0·7 | Low ROB | Low ROB | Low ROB | Low ROB |
| NACP, 2005 [15] (round I) | 2005 | 368 | 0 | Low ROB | Low ROB | Low ROB | Low ROB |
| NACP, 2007 [140] (round II) | 2006 | 194 | 0 | Low ROB | Low ROB | Low ROB | Low ROB |
| NACP, 2007 [140] (round II) | 2006 | 400 | 0 | Low ROB | Low ROB | Low ROB | Low ROB |
| NACP, 2007 [140] (round II) | 2006 | 400 | 0 | Low ROB | Low ROB | Low ROB | Low ROB |
| NACP, 2007 [140] (round II) | 2006 | 398 | 0·3 | Low ROB | Low ROB | Low ROB | Low ROB |
| NACP, 2007 [140] (round II) | 2006 | 403 | 0 | Low ROB | Low ROB | Low ROB | Low ROB |
| NACP, 2007 [140] (round II) | 2006 | 425 | 0·02 | Low ROB | Low ROB | Low ROB | Low ROB |
| NACP, 2007 [140] (round II) | 2006 | 400 | 0 | Low ROB | Low ROB | Low ROB | Low ROB |
| NACP, 2007 [140] (round II) | 2006 | 400 | 0 | Low ROB | Low ROB | Low ROB | Low ROB |
| NACP, 2007 [140] (round II) | 2006 | 423 | 0 | Low ROB | Low ROB | Low ROB | Low ROB |
| NACP, 2007 [140] (round II) | 2006 | 398 | 0 | Low ROB | Low ROB | Low ROB | Low ROB |
| NACP, 2007 [140] (round II) | 2006 | 400 | 0 | Low ROB | Low ROB | Low ROB | Low ROB |
| NACP, 2007 [140] (round II) | 2006 | 400 | 0 | Low ROB | Low ROB | Low ROB | Low ROB |
| Hawkes, 2009 [141] | 2007 | 107 | 0 | Low ROB | Low ROB | Low ROB | Low ROB |
| Hawkes, 2009 [141] | 2007 | 426 | 0 | Low ROB | Low ROB | Unclear | Low ROB |
| Khan, 2011 [17] | 2007 | 730 | 0·7 | Low ROB | Low ROB | Unclear | Low ROB |
| NACP, 2010 [142] (special IBBSS among FSWs) | 2009 | 2,197 | 1·0 | Unclear | Unclear | Unclear | Low ROB |
| NACP, 2012 [20] (round IV) | 2012 | 375 | 0·5 | Low ROB | Low ROB | Low ROB | Low ROB |
| NACP, 2012 [20] (round IV) | 2012 | 376 | 0 | Low ROB | Low ROB | Low ROB | Low ROB |
| NACP, 2012 [20] (round IV) | 2012 | 211 | 0·9 | Low ROB | Low ROB | Low ROB | Low ROB |
| NACP, 2012 [20] (round IV) | 2012 | 377 | 1·9 | Low ROB | Low ROB | Low ROB | Low ROB |
| NACP, 2012 [20] (round IV) | 2012 | 375 | 0·5 | Low ROB | Low ROB | Low ROB | Low ROB |
| NACP, 2012 [20] (round IV) | 2012 | 375 | 1·9 | Low ROB | Low ROB | Low ROB | Low ROB |
| NACP, 2012 [20] (round IV) | 2012 | 375 | 0·3 | Low ROB | Low ROB | Low ROB | Low ROB |
| NACP, 2012 [20] (round IV) | 2012 | 367 | 0 | Low ROB | Low ROB | Low ROB | Low ROB |
| NACP, 2012 [20] (round IV) | 2012 | 345 | 0 | Low ROB | Low ROB | Low ROB | Low ROB |
| NACP, 2012 [20] (round IV) | 2012 | 375 | 0 | Low ROB | Low ROB | High ROB | Low ROB |
| NACP, 2012 [20] (round IV) | 2012 | 345 | 0.3 | Low ROB | Low ROB | Low ROB | Low ROB |
| NACP, 2012 [20] (round IV) | 2012 | 375 | 0.8 | Low ROB | Low ROB | Low ROB | Low ROB |
| NACP, 2017 [22] (round V) | 2016-17 | 351 | 0 | Low ROB | Low ROB | Low ROB | Low ROB |
| NACP, 2017 [22] (round V) | 2016-17 | 196 | 1.5 | Low ROB | Low ROB | High ROB | Low ROB |
| NACP, 2017 [22] (round V) | 2016-17 | 364 | 0.8 | Low ROB | Low ROB | Low ROB | Low ROB |
| NACP, 2017 [22] (round V) | 2016-17 | 304 | 0.7 | Low ROB | Low ROB | Low ROB | Low ROB |
| NACP, 2017 [22] (round V) | 2016-17 | 250 | 0.4 | Low ROB | Low ROB | Low ROB | Low ROB |
| NACP, 2017 [22] (round V) | 2016-17 | 364 | 2.2 | Low ROB | Low ROB | Low ROB | Low ROB |
| NACP, 2017 [22] (round V) | 2016-17 | 387 | 2.6 | Low ROB | Low ROB | Low ROB | Low ROB |
| NACP, 2017 [22] (round V) | 2016-17 | 364 | 0 | Low ROB | Low ROB | Low ROB | Low ROB |
| NACP, 2017 [22] (round V) | 2016-17 | 364 | 4.1 | Low ROB | Low ROB | Low ROB | Low ROB |
| NACP, 2017 [22] (round V) | 2016-17 | 364 | 4.1 | Low ROB | Low ROB | Low ROB | Low ROB |
| NACP, 2017 [22] (round V) | 2016-17 | 364 | 3.8 | Low ROB | Low ROB | Low ROB | Low ROB |
| NACP, 2017 [22] (round V) | 2016-17 | 265 | 3 | Low ROB | Low ROB | Low ROB | Low ROB |
| NACP, 2017 [22] (round V) | 2016-17 | 364 | 0 | Low ROB | Low ROB | Low ROB | Low ROB |
| NACP, 2017 [22] (round V) | 2016-17 | 364 | 0.3 | Low ROB | Low ROB | Low ROB | Low ROB |
| NACP, 2017 [22] (round V) | 2016-17 | 363 | 1.7 | Low ROB | Low ROB | Low ROB | Low ROB |
| NACP, 2017 [22] (round V) | 2016-17 | 364 | 8.8 | Low ROB | Low ROB | Low ROB | Low ROB |
| NACP, 2017 [22] (round V) | 2016-17 | 193 | 0 | Low ROB | Low ROB | High ROB | Low ROB |
| NACP, 2017 [22] (round V) | 2016-17 | 72 | 0 | Low ROB | Low ROB | High ROB | Low ROB |
| **Somalia** |  |  |  |  |  |  |  |
| Testa, 2008 [143] (round I) | 2008 | 237 | 5.2 | Low ROB | Low ROB | Low ROB | Low ROB |
| IOM, 2017 [144] (round II) | 2014 | 96 | 4.8 | Low ROB | Low ROB | High ROB | Low ROB |
| **Sudan** |  |  |  |  |  |  |  |
| Elkarim, 2002 [145] | 2002 | 367 | 4.4 | Low ROB | Low ROB | Unclear | Low ROB |
| Abdelrahim, 2010 [146] | 2008 | 321 | 0.9 | Low ROB | Low ROB | Low ROB | Low ROB |
| NACP, 2010 [147] | 2008-09 | 267 | 0.1 | Unclear | Low ROB | Unclear | Low ROB |
| NACP, 2012 [148] | 2011 | 305 | 0.3 | Low ROB | Low ROB | Low ROB | Low ROB |
| NACP, 2012 [148] | 2011 | 279 | 1.5 | Low ROB | Low ROB | Low ROB | Low ROB |
| NACP, 2012 [148] | 2011 | 282 | 0.6 | Low ROB | Low ROB | Low ROB | Low ROB |
| NACP, 2012 [148] | 2011 | 296 | 0.7 | Low ROB | Low ROB | Low ROB | Low ROB |
| NACP, 2012 [148] | 2011 | 288 | 5.0 | Low ROB | Low ROB | Low ROB | Low ROB |
| NACP, 2012 [148] | 2011 | 287 | 0 | Low ROB | Low ROB | Low ROB | Low ROB |
| NACP, 2012 [148] | 2011 | 303 | 0.7 | Low ROB | Low ROB | Low ROB | Low ROB |
| NACP, 2012 [148] | 2011 | 296 | 1 | Low ROB | Low ROB | Low ROB | Low ROB |
| NACP, 2012 [148] | 2011 | 293 | 7.7 | Low ROB | Low ROB | Low ROB | Low ROB |
| NACP, 2012 [148] | 2011 | 291 | 0.7 | Low ROB | Low ROB | Low ROB | Low ROB |
| NACP, 2012 [148] | 2011 | 303 | 0.7 | Low ROB | Low ROB | Low ROB | Low ROB |
| NACP, 2012 [148] | 2011 | 299 | 0.2 | Low ROB | Low ROB | Low ROB | Low ROB |
| NACP, 2012 [148] | 2011 | 284 | 1 | Low ROB | Low ROB | Low ROB | Low ROB |
| NACP, 2012 [148] | 2011 | 288 | 1.3 | Low ROB | Low ROB | Low ROB | Low ROB |
| MOH, 2016 [30] | 2015-16 | 835 | 37.9 | Low ROB | Low ROB | Low ROB | Low ROB |
| **Tunisia** |  |  |  |  |  |  |  |
| Hsairi, 2012 [31] | 2009 | 703 | 0.4 | Low ROB | Low ROB | Unclear | Low ROB |
| Hsairi, 2012 [31] | 2011 | 357 | 0.6 | Low ROB | Low ROB | Low ROB | Low ROB |
| Hsairi, 2012 [31] | 2011 | 284 | 0 | Low ROB | Low ROB | Low ROB | Low ROB |
| Hsairi, 2012 [31] | 2011 | 347 | 1.2 | Low ROB | Low ROB | Low ROB | Low ROB |
| **Yemen** |  |  |  |  |  |  |  |
| Stulhofer, 2008 [149] (round I) | 2008 | 244 | 1.3 | Unclear | Low ROB | Unclear | Low ROB |
| MOH, 2014 [150] (round I) | 2010-11 | 301 | 0 | Unclear | Low ROB | Unclear | Low ROB |
| **Studies using non-probability sampling** | | | | | | | |
| **Afghanistan** |  |  |  |  |  |  |  |
| Todd, 2010 [151] | 2006-08 | 520 | 0.2 | Low ROB | High ROB | Unclear | Low ROB |
| **Djibouti** |  |  |  |  |  |  |  |
| Rodier, 1993 [152] | 1987 | 66 | 4.6 | Low ROB | High ROB | Unclear | Low ROB |
| Rodier, 1993 [152] | 1987 | 221 | 1.4 | Low ROB | High ROB | Unclear | Low ROB |
| Constantine, 1992 [153] | 1988 | 33 | 18.2 | Unclear | High ROB | Unclear | Low ROB |
| Rodier, 1993 [152] | 1988 | 78 | 9.0 | Low ROB | High ROB | Unclear | Low ROB |
| Rodier, 1993 [152] | 1988 | 255 | 2.7 | Low ROB | High ROB | Unclear | Low ROB |
| Rodier, 1993 [152] | 1990 | 116 | 41.7 | Low ROB | High ROB | Unclear | Low ROB |
| Rodier, 1993 [152] | 1990 | 180 | 5.0 | Low ROB | High ROB | Unclear | Low ROB |
| Couzineau, 1991 [154] | 1991 | 300 | 43 | Unclear | High ROB | Unclear | Low ROB |
| Couzineau, 1991 [154] | 1991 | 397 | 13.1 | Unclear | High ROB | Unclear | Low ROB |
| Rodier, 1993 [152] | 1991 | 292 | 36.0 | Low ROB | High ROB | Unclear | Low ROB |
| Rodier, 1993 [152] | 1991 | 360 | 15.3 | Low ROB | High ROB | Unclear | Low ROB |
| Philippon, 1997 [155] | 1995 | 176 | 49 | Unclear | High ROB | Unclear | Low ROB |
| Marcelin, 2002 [156] | 1998-99 | 43 | 70 | Unclear | High ROB | Unclear | Low ROB |
| Marcelin, 2002 [156] | 1998-99 | 123 | 7 | Unclear | High ROB | Unclear | Low ROB |
| **Egypt** |  |  |  |  |  |  |  |
| Sheba, 1988 [157] | 1986-87 | 87 | 0 | Unclear | High ROB | Unclear | Low ROB |
| Watts, 1993[158] | 1986-90 | 349 | 0 | Unclear | High ROB | Unclear | Low ROB |
| Kabbash, 2012 [159] | 2009-10 | 431 | 0 | Unclear | High ROB | Low ROB | Low ROB |
| **Iran** |  |  |  |  |  |  |  |
| Jahani, 2005 [160] | 2002 | 149 | 0 | Unclear | High ROB | Unclear | Low ROB |
| Kassaian, 2012 [161] | 2009-10 | 91 | 0 | Low ROB | High ROB | Low ROB | Low ROB |
| Taghizadeh, 2015 [162] | 2014 | 184 | 4 | Unclear | High ROB | Low ROB | Low ROB |
| Asadi-Ali, 2018 [163] | 2015 | 133 | 1.5 | Low ROB | High ROB | Low ROB | Low ROB |
| **Lebanon** |  |  |  |  |  |  |  |
| Naman, 1989 [164] | 1985-87 | 291 | 0.3 | Unclear | High ROB | Unclear | Low ROB |
| **Morocco** |  |  |  |  |  |  |  |
| MOH, 2008 [165] | 2007 | 141 | 1.4 | Unclear | High ROB | Low ROB | Low ROB |
| **Pakistan** |  |  |  |  |  |  |  |
| Iqbal, 1996 [166] | 1987-94 | 21 | 0 | Unclear | High ROB | Unclear | Low ROB |
| Baqi, 1998 [167] | 1993-94 | 77 | 0 | Low ROB | High ROB | Low ROB | Low ROB |
| Anwar, 1998 [168] | NR | 103 | 1.9 | Unclear | Unclear | Unclear | Low ROB |
| Bokhari, 2007 [139] | 2004 | 421 | 0 | Low ROB | High ROB | Low ROB | Low ROB |
| Shah, 2004 [169] | 2004 | 157 | 0 | Unclear | High ROB | Unclear | Low ROB |
| Shah, 2004 [170] | 2004 | 163 | 1.2 | Unclear | High ROB | Unclear | Low ROB |
| Akhtar, 2008 [171] | 2007 | 246 | 0 | Unclear | Unclear | Unclear | Low ROB |
| Raza, 2015 [172] | 2014 | NR | 0 | Unclear | High ROB | Unclear | Low ROB |
| **Somalia** |  |  |  |  |  |  |  |
| Jama, 1987 [173] | 1985-86 | 85 | 0 | Unclear | High ROB | Unclear | Low ROB |
| Burans, 1990 [174] | NR | 89 | 0 | Unclear | High ROB | Low ROB | Low ROB |
| Scott, 1991 [175] | 1989 | 57 | 0 | Unclear | High ROB | Unclear | Low ROB |
| Corwin, 1991 [176] | 1990 | 302 | 3 | Unclear | High ROB | Unclear | Low ROB |
| Jama Ahmed, 1991 [177] | 1991 | 155 | 0.6 | Unclear | High ROB | Unclear | Low ROB |
| **Sudan** |  |  |  |  |  |  |  |
| Burans, 1990 [178] | 1987 | 203 | 0 | Low ROB | High ROB | Unclear | Low ROB |
| McCarthy, 1995 [179] | NR | 50 | 16 | Unclear | High ROB | Low ROB | Low ROB |
| **Tunisia** |  |  |  |  |  |  |  |
| Bchir, 1988 [180] | 1987 | 42 | 0 | Low ROB | High ROB | Unclear | Low ROB |
| Hassen, 2003 [181] | NR | 51 | 0 | Low ROB | High ROB | Low ROB | Low ROB |
| Znazen, 2010 [182] | 2007 | 183 | 0 | Low ROB | High ROB | Low ROB | Low ROB |

The table is sorted by year(s) of data collection.

*Abbreviations*: *FSWs* female sex workers, *IBBSS* integrated bio-behavioural surveillance survey, *IOM* International Organization for Migration, *MOH* Ministry of Health, *NACP* National AIDS Control Programme, *NAP* National AIDS Program, *NR* not reported, *Prev* prevalence, *SAR AIDS HDS* South Asia Region AIDS Human Development Sector, *WHO* World Health Organization

**Table S9** Risk of bias (ROB) assessment of HIV prevalence studies in clients of FSWs (or proxy populations of clients) in the Middle East and North Africa

| **Country**  **Author, year [citation]** | **Year(s) of data collection** | **Sample size** | **HIV prev (%)** | **Sex work definition** | **Sampling method** | **Response rate** | **HIV ascertainment** |
| --- | --- | --- | --- | --- | --- | --- | --- |
| **Djibouti** |  |  |  |  |  |  |  |
| Rodier, 1993 [152] | 1987 | 252 | 0.8 | Unclear | High ROB | Unclear | Low ROB |
| Rodier, 1993 [152] | 1988 | 249 | 0.8 | Unclear | High ROB | Unclear | Low ROB |
| Fox, 1989 [183] | NR | 105 | 1.0 | High ROB | High ROB | Unclear | Low ROB |
| Rodier, 1993 [152] | 1990 | 106 | 1.9 | Unclear | High ROB | Unclear | Low ROB |
| Rodier, 1993 [152] | 1991 | 193 | 10.4 | Unclear | High ROB | Unclear | Low ROB |
| **Egypt** |  |  |  |  |  |  |  |
| Sheba, 1988 [157] | 1986-87 | 302 | 0 | Unclear | High ROB | Unclear | Low ROB |
| **Kuwait** |  |  |  |  |  |  |  |
| Al-Owaish, 2000 [184] | 1996-97 | 617 | 0 | Low ROB | Low ROB | Unclear | Low ROB |
| Al-Owaish, 2000 [184] | 1996-97 | 1,367 | 0 | Low ROB | Low ROB | Unclear | Low ROB |
| Al-Owaish, 2002 [185] | 2002 | 599 | 0 | Unclear | High ROB | Unclear | Low ROB |
| Al-Mutairi, 2007 [186] | 2003-04 | 520 | 0 | Low ROB | High ROB | High ROB | Low ROB |
| **Morocco** |  |  |  |  |  |  |  |
| Heikel, 1999 [187] | 1992-96 | 1,131 | 0.9 | Unclear | High ROB | Unclear | Low ROB |
| Manhart, 1996 [188] | 1996 | 223 | 1.4 | Unclear | High ROB | Unclear | Low ROB |
| Alami, 2002 [189] | 2001 | 422 | 0 | Unclear | High ROB | Unclear | Low ROB |
| **Pakistan** |  |  |  |  |  |  |  |
| Mujeeb, 1993 [190] | NR | 32 | 0 | Unclear | High ROB | Unclear | Low ROB |
| Memon, 1997 [191] | 1994-95 | 50 | 0 | Unclear | High ROB | Unclear | Low ROB |
| NAP, 1996 [192] | 1995 | 402 | 0 | Unclear | High ROB | Unclear | Low ROB |
| NAP, 1996 [192] | 1995 | 295 | 0 | Unclear | High ROB | Unclear | Low ROB |
| Rehan, 2003 [193] | 1999 | 138 | 0 | Unclear | High ROB | Unclear | Low ROB |
| Rehan, 2003 [193] | 1999 | 148 | 0 | Unclear | High ROB | Unclear | Low ROB |
| Rehan, 2003 [193] | 1999 | 93 | 1.1 | Unclear | High ROB | Unclear | Low ROB |
| Rehan, 2003 [193] | 1999 | 86 | 0 | Unclear | High ROB | Unclear | Low ROB |
| Bhutto, 2011 [194] | 2000-09 | 4,288 | 0.06 | Low ROB | High ROB | Unclear | Low ROB |
| Bokhari, 2007 [139] | 2004 | 120 | 0 | Low ROB | Low ROB | Low ROB | Low ROB |
| Razvi, 2014 [195] | 2010-14 | 465 | 1.1 | Low ROB | High ROB | Unclear | Low ROB |
| NAP, 2012 [196] | 2011 | 381 | 0 | Low ROB | Low ROB | Low ROB | Low ROB |
| **Somalia** |  |  |  |  |  |  |  |
| Ismail, 1990 [41] | 1986 | 101 | 0 | Low ROB | High ROB | Unclear | Low ROB |
| Scott, 1991 [175] | 1989 | 50 | 0 | Unclear | High ROB | Unclear | Low ROB |
| Burans, 1990 [174] | NR | 45 | 0 | Low ROB | High ROB | Low ROB | Low ROB |
| Corwin, 1991 [176] | 1990 | 26 | 0 | Unclear | High ROB | Unclear | Low ROB |
| Ismail, 2007 [197] | 2007 | NR | 7.4 | Unclear | High ROB | Low ROB | Low ROB |
| **Sudan** |  |  |  |  |  |  |  |
| McCarthy, 1989 [198] | 1987 | 157 | 0 | Low ROB | High ROB | Unclear | Low ROB |
| McCarthy, 1989 [43] | 1987-88 | 398 | 2.5 | Low ROB | High ROB | Unclear | Low ROB |
| McCarthy, 1995 [179] | NR | 37 | 13.5 | Low ROB | High ROB | Unclear | Low ROB |

The table is sorted by year(s) of data collection or year of publication if year of data collection was not reported.

*Abbreviations*: *FSWs* female sex workers, *MOH* Ministry of Health, *NAP* National AIDS Program, *NR* not reported, *Prev* prevalence

**Table S10** Results of meta-regression analyses to identify associations with HIV prevalence, sources of between-study heterogeneity, and trend in HIV prevalence in clients of FSWs (or proxy populations of clients such as male STI clinic attendees), in the Middle East and North Africa (MENA)

|  |  | **Studies** | **Samples** | **Univariable analyses** | | | **Multivariable analysis** | | |
| --- | --- | --- | --- | --- | --- | --- | --- | --- | --- |
| **Sources of heterogeneity**^*^ |  | **Total N** | **Total N** | **OR (95% CI)** | **LR test p-value**^‡^ | **Variance explained R^2^**^£^ **(%)** | **AOR (95% CI)** | **p-value** | **LR test p-value**^¥^ |
| **Country/subregion**^**^ |  |  |  |  |  |  |  |  |  |
| Pakistan | Pakistan | 12 | 6,498 | 1.00 | <0.001 | 29.0 | 1.00 |  | <0.001 |
| Egypt | Egypt | 6 | 1,362 | 1.34 (0.28-6.30) |  |  | 1.56 (0.32-7.53) | 0.581 |  |
| Kuwait & Yemen | Kuwait & Yemen^†^ | 7 | 6,535 | 0.24 (0.06-1.06) |  |  | 0.26 (0.06-1.13) | 0.072 |  |
| Horn of Africa | Djibouti, Somalia, South Sudan | 27 | 3,269 | 19.58 (6.69-57.36) |  |  | 17.85 (6.02-52.87) | <0.001 |  |
| North Africa | Algeria, Morocco, Sudan | 95 | 11,867 | 3.00 (1.16-7.76) |  |  | 2.77 (0.95-8.05) | 0.062 |  |
|  |  |  |  |  |  |  |  |  |  |
| **Total sample size of tested clients/male STI clinic attendees** | <100 | 18 | 502 | 1.00 | 0.021 | 3.0 | 1.00 |  | 0.271 |
|  | ≥100 | 129 | 29,029 | 0.34 (0.14-0.84) |  |  | 0.63 (0.28-1.44) | 0.271 |  |
|  |  |  |  |  |  |  |  |  |  |
| **Median year of data collection**^⁑^ | <2003 | 42 | 13,889 | 1.00 | 0.506 | 0 | 1.00 |  | 0.588 |
|  | ≥2003 | 105 | 15,642 | 1.25 (0.64-2.46) |  |  | 1.24 (0.57-2.72) | 0.588 |  |

^*^Only country, sample size, and year of data collection had sufficient number of studies to warrant conduct of meta-regression analyses.

^**^Countries were grouped based on geography and similarity in HIV prevalence levels. Given the large fraction of studies with zero HIV prevalence, particularly in the Fertile Crescent, an increment of 0.1 was added to number of events in all studies when generating log odds, and Eastern MENA was thus used also as a statistically better reference. While this choice of increment was arbitrary, other increments yielded the same findings, though some of the effect sizes changed in scale.

^⁑^Year grouping was driven by independent evidence identifying the emergence of HIV epidemics among both men who have sex with men[3] and people who inject drugs[4] in multiple MENA countries around 2003. Missing values for year of data collection (only four stratified measures) were imputed using data for year of publication adjusted by the median difference between year of publication and median year of data collection (for studies with complete information).

^†^Only one study was from Yemen.

^‡^Predictors with p-value ≤0.1 were considered as showing strong evidence for an association with (prevalence) odds, and were hence included in the multivariable analysis. Median year was also included in the multivariable model given its importance.

^£^Adjusted R-squared in the final multivariable model=28.78%

^¥^Predictors with p-value ≤0.1 in the multivariable model were considered as showing strong evidence for an association with (prevalence) odds.

*Abbreviations*: *AOR* adjusted odds ratio, *CI* confidence interval, *Coll* collection, *FSWs* female sex workers, *LR* likelihood ratio, *OR* odds ratio, *STI* sexually transmitted infection

**Table S11** Condom use among FSWs and their clients in the Middle East and North Africa

| **Country**  **Author, year [citation]** | **Year(s) of data collection** | **City/province** | **Population** | **Condom use** | | |
| --- | --- | --- | --- | --- | --- | --- |
|  |  |  |  | **Time frame** | **Use (%)** | **Consistent use (always/most of the time among all FSWs) (%)** |
| **FSWS** |  |  |  |  |  |  |
| **VAGINAL SEX** |  |  |  |  |  |  |
| **With client** |  |  |  |  |  |  |
| **Afghanistan** |  |  |  |  |  |  |
| SAR AIDS HDS, 2008 [5] | 2006-07 | Jalalabad | All FSWs | Ever | 29.0 | 16.0 |
| SAR AIDS HDS, 2008 [5] | 2006-07 | Mazar-i-Sharif | All FSWs | Ever | 40.0 | 32.0 |
| Todd, 2010 [151] | 2006-08 | Kabul, Jalalabad, Mazar-i-Sharif | All FSWs | Ever | 30.2 | 38.2^*^ |
| NACP, 2010 [128] | 2009 | Kabul | All FSWs | Last sex | 58.1 | NR |
| NACP, 2012 [6] | 2012 | Herat | All FSWs | Last sex | 67.0 | NR |
| NACP, 2012 [6] | 2012 | Kabul | All FSWs | Last sex | 64.0 | NR |
| NACP, 2012 [6] | 2012 | Mazar-i-Sharif | All FSWs | Last sex | 26.1 | NR |
| **Algeria** |  |  |  |  |  |  |
| MOH, 2014 [53] | 2014 | Saida | All FSWs | Last sex | 84.1 | NR |
| **Djibouti** |  |  |  |  |  |  |
| Rodier, 1993 [152] | 1990 | Djibouti | All FSWs | NR | NR | 41.9 |
| Rodier, 1993 [152] | 1990 | Djibouti | All bar girls | NR | NR | 92.7 |
| Rodier, 1993 [152] | 1991 | Djibouti | All FSWs | NR | NR | 28.4 |
| Rodier, 1993 [152] | 1991 | Djibouti | All bar girls | NR | NR | 90.9 |
| Philippon, 1997 [155] | 1995 | Djibouti | All FSWs | NR | 86.0 | 48.0 |
| Trellu-Kane, 2005 [7] | 2005 | Djibouti | All FSWs | Last sex | 25.0 | NR |
| MOH, 2010 [65] | 2007 | Djibouti | All FSWs | Last sex | 94.2 | NR |
| **Egypt** |  |  |  |  |  |  |
| MOH, 2006 [129] | 2006 | Cairo | All FSWs | Last sex | 31.4 | NR |
| Kabbash, 2012 [159] | 2009-10 | Cairo | FSWs who heard of condoms | Last sex | 22.4 | 16.7^†^ |
| Kabbash, 2012 [159] | 2009-10 | Cairo | FSWs who heard of condoms | Past 1 M | 32.6 | NR |
| MOH, 2010 [130] | 2010 | Cairo | All FSWs | Last sex | 25.0 | 16.5 |
| MOH, 2010 [130] | 2010 | Cairo | All FSWs | Past 1 M | 41.0 | NR |
| NAP, 2014 [71] | 2010 | Cairo | All FSWs | Last sex | 10.0 | NR |
| **Iran** |  |  |  |  |  |  |
| Jahani, 2005 [160] | 2002 | NR | All FSWs | NR | NR | 83.2 |
| Kassaian, 2012 [161] | 2009-10 | Isfahan | All FSWs | NR | 64.8 | 48.4 |
| Sajadi, 2013 [132] | 2010 | National | All FSWs | Last sex | 57.1 | 49.1 |
| Kazerooni, 2014 [133] | 2010-11 | Shiraz | All FSWs | Last sex | 54.0 | 45.3^*^ |
| Kazerooni, 2014 [133] | 2010-11 | Shiraz | All FSWs | Past 1 M | 79.8 | NR |
| Moayedi-Nia, 2016 [134] | 2012-13 | Tehran | All FSWs | Last sex | 65.2 | NR |
| Taghizadeh, 2015 [162] | 2014 | Sari | All FSWs | Last sex | 78.5 | 62.4 |
| Asadi-Ali, 2018 [163] | 2015 | Northern Iran | All FSWs | Last sex | 43.3 | 42.3^*^ |
| Asadi-Ali, 2018 [163] | 2015 | Northern Iran | All FSWs | Ever | 83.6 | NR |
| Mirzazadeh, 2016 [135] | 2015 | National | NR | NR | NR | 26.0 |
| Karami, 2017 [11] | 2016 | Tehran | All FSWs | Last sex | 56.1 | 39.3 |
| Navadeh, 2012 [131] | 2010 | Kerman | All FSWs | Last sex | 83.1 | NR |
| **Jordan** |  |  |  |  |  |  |
| MOH, 2010 [199] | 2009 | 4 governorates | All FSWs | Last sex | 51.0 | NR |
| MOH, 2014 [136] | 2013 | Amman | All FSWs | Last sex | 80.0 | NR |
| MOH, 2014 [136] | 2013 | Irbid | All FSWs | Last sex | 67.0 | NR |
| **Morocco** |  |  |  |  |  |  |
| MOH, 2006 [86] | 2003 | NR | All FSWs | Last sex | 37.3 | NR |
| MOH, 2008 [165] | 2007 | Agadir, Rabat Sale, Tanger | Al FSWs | NR | 83.0 | 40.4 |
| MOH, 2012 [12] | 2011-12 | Agadir | All FSWs | Last sex | 42.0 | 28.7 |
| MOH, 2012 [12] | 2011-12 | Fes | All FSWs | Last sex | 49.5 | 26.3 |
| MOH, 2012 [12] | 2011-12 | Rabat | All FSWs | Last sex | 51.1 | 34.6 |
| MOH, 2012 [12] | 2011-12 | Tanger | All FSWs | Last sex | 63.1 | 58.3 |
| MOH, 2013 [120] | 2013 | National | All FSWs | Past 12 M | 61.0 | 6.4 |
| **Lebanon** |  |  |  |  |  |  |
| Mahfoud, 2010 [137] | 2007-08 | Greater Beirut | All FSWs | Past 1 M | 97.7 | 95.2 |
| **Pakistan** |  |  |  |  |  |  |
| Baqi, 1998 [167] | 1993-94 | Karachi | All FSWs | Ever | 9.8 | 0 |
| NACP, 2005[200] | 2004 | Karachi | All FSWs | Last sex | 25.0 | NR |
| NACP, 2005 [200] | 2004 | Lahore | All FSWs | Last sex | 53.0 | NR |
| NACP, 2005 [14] | 2004-05 | Karachi | All FSWs | Last sex | 36.7 | 18.1 |
| NACP, 2005 [14] | 2004-05 | Rawalpindi | All FSWs | Last sex | 49.3 | 16.7 |
| NACP, 2005 [15] | 2005 | Faisalabad | All FSWs | Last sex | 19.0 | 3.0 |
| NACP, 2005 [15] | 2005 | Hyderabad | All FSWs | Last sex | 17.0 | 13.0 |
| NACP, 2005 [15] | 2005 | Karachi | All FSWs | Last sex | 50.0 | 30.0 |
| NACP, 2005 [15] | 2005 | Lahore | All FSWs | Last sex | 68.0 | 42.0 |
| NACP, 2005 [15] | 2005 | Multan | All FSWs | Last sex | 35.0 | 14.0 |
| NACP, 2005 [15] | 2005 | Peshawar | All FSWs | Last sex | 23.0 | 11.0 |
| NACP, 2005 [15] | 2005 | Quetta | All FSWs | Last sex | 40.0 | 16.0 |
| NACP, 2005 [15] | 2005 | Sukkur | All FSWs | Last sex | 17.0 | 13.0 |
| NACP, 2007 [140] | 2006 | National | All FSWs | Last sex | 45.0 | 23.0 |
| NACP, 2007 [140] | 2006 | Bannu | All FSWs | NR | NR | 5.0 |
| NACP, 2007 [140] | 2006 | Faisalabad | All FSWs | NR | NR | 16.0 |
| NACP, 2007 [140] | 2006 | Gujranwala | All FSWs | NR | NR | 12.0 |
| NACP, 2007 [140] | 2006 | Hyderabad | All FSWs | NR | NR | 36.0 |
| NACP, 2007 [140] | 2006 | Karachi | All FSWs | NR | NR | 44.0 |
| NACP, 2007 [140] | 2006 | Lahore | All FSWs | NR | NR | 31.0 |
| NACP, 2007 [140] | 2006 | Larkana | All FSWs | NR | NR | 28.0 |
| NACP, 2007 [140] | 2006 | Multan | All FSWs | NR | NR | 5.0 |
| NACP, 2007 [140] | 2006 | Peshawar | All FSWs | NR | NR | 33.0 |
| NACP, 2007 [140] | 2006 | Quetta | All FSWs | NR | NR | 33.0 |
| NACP, 2007 [140] | 2006 | Rawalpindi | All FSWs | NR | NR | 31.0 |
| NACP, 2007 [140] | 2006 | Sargodha | All FSWs | NR | NR | 12.0 |
| NACP, 2007 [140] | 2006 | Sukkur | All FSWs | NR | NR | 7.0 |
| Hawkes, 2009 [141] | 2007 | Abbottabad, Rawalpindi | All FSWs | Last sex | 38.0 | 12.0 |
| Khan, 2011 [17] | 2007 | Lahore | All FSWs | NR | NR | 65.0 |
| NACP, 2010 [142] | 2009 | Punjab | All FSWs | Last sex | 43.3 | NR |
| NACP, 2012 [20] | 2011 | DG Khan | All FSWs | Last sex | 32.0 | 8.0 |
| NACP, 2012 [20] | 2011 | Faisalabad | All FSWs | Last sex | 43.0 | 30.0 |
| NACP, 2012 [20] | 2011 | Karachi | All FSWs | Last sex | 67.0 | 48.0 |
| NACP, 2012 [20] | 2011 | Haripur | All FSWs | Last sex | 44.0 | 24.0 |
| NACP, 2012 [20] | 2011 | Lahore | All FSWs | Last sex | 46.0 | 31.0 |
| NACP, 2012 [20] | 2011 | Larkana | All FSWs | Last sex | 58.0 | 53.0 |
| NACP, 2012 [20] | 2011 | Multan | All FSWs | Last sex | 48.0 | 24.0 |
| NACP, 2012 [20] | 2011 | Peshawar | All FSWs | Last sex | 43.0 | 27.0 |
| NACP, 2012 [20] | 2011 | Quetta | All FSWs | Last sex | 57.0 | 38.0 |
| NACP, 2012 [20] | 2011 | Rawalpindi | All FSWs | Last sex | 14.0 | 8.0 |
| NACP, 2012 [20] | 2011 | Sargodha | All FSWs | Last sex | 35.5 | 14.0 |
| NACP, 2012 [20] | 2011 | Sukkur | All FSWs | Last sex | 21.0 | 5.0 |
| Punjab NACP, 2015 [201] | 2014 | Faisalabad | All FSWs | Last sex | 71.2 | 38.2 |
| Punjab NACP, 2015 [201] | 2014 | Lahore | All FSWs | Last sex | 66.2 | 32.4 |
| Punjab NACP, 2015 [201] | 2014 | Multan | All FSWs | Last sex | 68.4 | 34.6 |
| Punjab NACP, 2015 [201] | 2014 | Sargodha | All FSWs | Last sex | 74.4 | 37.2 |
| NACP, 2017 [22] | 2016-17 | Bahawalpur | All FSWs | Last sex | 58.0 | 39.8 |
| NACP, 2017 [22] | 2016-17 | Bannu | All FSWs | Last sex | 74.0 | 46.4 |
| NACP, 2017 [22] | 2016-17 | DG Khan | All FSWs | Last sex | 65.1 | 29.4 |
| NACP, 2017 [22] | 2016-17 | Gujranwala | All FSWs | Last sex | 65.8 | 65.5 |
| NACP, 2017 [22] | 2016-17 | Gujrat | All FSWs | Last sex | 31.0 | 16.7 |
| NACP, 2017 [22] | 2016-17 | Hyderabad | All FSWs | Last sex | 59.9 | 37.9 |
| NACP, 2017 [22] | 2016-17 | Larkana | All FSWs | Last sex | 11.8 | 11.3 |
| NACP, 2017 [22] | 2016-17 | Karachi | All FSWs | Last sex | 61.5 | 45.5 |
| NACP, 2017 [22] | 2016-17 | Kasur | All FSWs | Last sex | 29.4 | 23.6 |
| NACP, 2017 [22] | 2016-17 | Mirpurkhas | All FSWs | Last sex | 28.8 | 17.3 |
| NACP, 2017 [22] | 2016-17 | Nawabshah | All FSWs | Last sex | 14.8 | 4.7 |
| NACP, 2017 [22] | 2016-17 | Peshawar | All FSWs | Last sex | 67.9 | 46.8 |
| NACP, 2017 [22] | 2016-17 | Quetta | All FSWs | Last sex | 89.8 | 75.0 |
| NACP, 2017 [22] | 2016-17 | Rawalpindi | All FSWs | Last sex | 4.1 | 1.1 |
| NACP, 2017 [22] | 2016-17 | Sheikhupura | All FSWs | Last sex | 74.4 | 72.7 |
| NACP, 2017 [22] | 2016-17 | Sialkot | All FSWs | Last sex | 94.8 | 93.3 |
| NACP, 2017 [22] | 2016-17 | Sukkur | All FSWs | Last sex | 61.4 | 55.8 |
| NACP, 2017 [22] | 2016-17 | Turbat | All FSWs | Last sex | 45.8 | 12.5 |
| **Somalia** |  |  |  |  |  |  |
| Testa, 2008 [143] | 2008 | Hargeisa | All FSWs | Last sex | 25.6 | 6.0 |
| IOM, 2017 [144] | 2014 | Hargeisa | All FSWs | Last sex | 31.5 | 17.5 |
| **Sudan** |  |  |  |  |  |  |
| Elkarim, 2002 [145] | 2002 | National | All FSWs | Last sex | 1.2 | 0.9 |
| Abdelrahim, 2010 [146] | 2008 | Khartoum | All FSWs | Last sex | 45.0 | 35.9 |
| Elhadi, 2013 [202] | 2011 | Alshamalia | All FSWs | Last sex | 41.0 | 24.1 |
| Elhadi, 2013 [202] | 2011 | Blue Nile | All FSWs | Last sex | 4.7 | 23.9 |
| Elhadi, 2013 [202] | 2011 | Gadarif | All FSWs | Last sex | 16.2 | 12.4 |
| Elhadi, 2013 [202] | 2011 | Gezira | All FSWs | Last sex | 8.2 | 5.0 |
| Elhadi, 2013 [202] | 2011 | Kassala | All FSWs | Last sex | 55.1 | 0.7 |
| Elhadi, 2013 [202] | 2011 | Khartoum | All FSWs | Last sex | 30.3 | 18.5 |
| Elhadi, 2013 [202] | 2011 | North Darfur | All FSWs | Last sex | 23.0 | 11.4 |
| Elhadi, 2013 [202] | 2011 | North Kodofan | All FSWs | Last sex | 15.8 | 8.9 |
| Elhadi, 2013 [202] | 2011 | Red Sea | All FSWs | Last sex | 18.7 | 13.7 |
| Elhadi, 2013 [202] | 2011 | River Nile | All FSWs | Last sex | 28.8 | 18.6 |
| Elhadi, 2013 [202] | 2011 | Sinnar | All FSWs | Last sex | 8.4 | 3.1 |
| Elhadi, 2013 [202] | 2011 | South Darfur | All FSWs | Last sex | 21.6 | 24.5 |
| Elhadi, 2013 [202] | 2011 | West Darfur | All FSWs | Last sex | 14.6 | 7.6 |
| Elhadi, 2013 [202] | 2011 | White Nile | All FSWs | Last sex | 12.5 | 5.0 |
| MOH, 2016 [30] | 2015-16 | Juba, South Sudan | All FSWs | Last sex | 72.4 | 72.4 |
| **Syria** |  |  |  |  |  |  |
| MOH, 2005 [104] | 2005 | NR | All FSWs | NR | 84.8 | 33.8 |
| **Tunisia** |  |  |  |  |  |  |
| Znazen, 2010 [182] | 2007 | Gabes, Sousse, Tunis | All FSWs | NR | NR | 60.6 |
| Hassen, 2003 [181] | NR | Sousse | All FSWs | NR | 65.0 | 36.8 |
| MOH, 2010 [203] | 2009 | Sfax, Sousse, Tunis | All FSWs | Last sex | 51.6 | 23.7 |
| **Yemen** |  |  |  |  |  |  |
| Stulhofer, 2008 [149] | 2008 | Aden | All FSWs | Last sex | 57.1 | NR |
| MOH, 2014 [150] | 2010 | Hodeida | All FSWs | Last sex | 34.9 | NR |
| **With regular client** |  |  |  |  |  |  |
| **Lebanon** |  |  |  |  |  |  |
| Mahfoud, 2010 [137] | 2007-08 | Greater Beirut | FSWs with regular client in past 1 M | Last sex | 92.0 | 99.0 |
| **Libya** |  |  |  |  |  |  |
| Valadez, 2013 [138] | 2010-11 | Tripoli | FSWs with regular client in past 6 M | Last sex | 76.7 | 56.8 |
| **Morocco** |  |  |  |  |  |  |
| MOH, 2012 [12] | 2011-12 | Agadir | FSWs with regular client in past 1 M | Last sex | 50.1 | 69.3^*^ |
| MOH, 2012 [12] | 2011-12 | Fes | FSWs with regular client in past 1 M | Last sex | 43.2 | 56.9^*^ |
| MOH, 2012 [12] | 2011-12 | Rabat | FSWs with regular client in past 1 M | Last sex | 55.9 | 81.7^*^ |
| MOH, 2012 [12] | 2011-12 | Tanger | FSWs with regular client in past 1 M | Last sex | 68.9 | 85.0^*^ |
| **Pakistan** |  |  |  |  |  |  |
| Bokhari, 2007 [139] | 2004 | Karachi | FSWs with regular client in past 7 days | Last sex | 25.5 | 3.3 |
| Bokhari, 2007 [139] | 2004 | Lahore | FSWs with regular client in past 7 days | Last sex | 47.0 | 20.1 |
| **Sudan** |  |  |  |  |  |  |
| MOH, 2016 [30] | 2015-16 | Juba, South Sudan | FSWs with regular client in past 6 M | Last sex | 68.0 | NR |
| **Tunisia** |  |  |  |  |  |  |
| Hsairi, 2012 [31] | 2011 | Sfax, Sousse, Tunis | FSWs with regular client in past 1 M | Last sex | 44.3 | 41.5 |
| **Yemen** |  |  |  |  |  |  |
| Stulhofer, 2008 [149] | 2008 | Aden | FSWs with regular client in past 1 M | Last sex | 56.7 | 57.8 |
| **With one-time client** |  |  |  |  |  |  |
| **Lebanon** |  |  |  |  |  |  |
| Mahfoud, 2010 [137] | 2007-08 | Greater Beirut | FSWs with one-time client in past 1 M | Last sex | 96.0 | 100 |
| **Libya** |  |  |  |  |  |  |
| Valadez, 2013 [138] | 2010-11 | Tripoli | FSWs with one-time client in past 6 M | Last sex | 83.1 | 63.4 |
| **Morocco** |  |  |  |  |  |  |
| MOH, 2012 [12] | 2011-12 | Agadir | FSWs with one-time client in past 1 M | Last sex | 58.3 | NR |
| MOH, 2012 [12] | 2011-12 | Fes | FSWs with one-time client in past 1 M | Last sex | 54.6 | NR |
| MOH, 2012 [12] | 2011-12 | Rabat | FSWs with one-time client in past 1 M | Last sex | 60.3 | NR |
| MOH, 2012 [12] | 2011-12 | Tanger | FSWs with one-time client in past 1 M | Last sex | 72.5 | NR |
| **Pakistan** |  |  |  |  |  |  |
| Bokhari, 2007 [139] | 2004 | Karachi | FSWs with one-time client in past 7 days | Last sex | 28.5 | 2.4 |
| Bokhari, 2007 [139] | 2004 | Lahore | FSWs with one-time client in past 7 days | Last sex | 47.9 | 21.8 |
| **Sudan** |  |  |  |  |  |  |
| MOH, 2016 [30] | 2015-16 | Juba, South Sudan | FSWs with one-time client in past 6 M | Last sex | 61.0 | NR |
| **Tunisia** |  |  |  |  |  |  |
| Hsairi, 2012 [31] | 2011 | Sfax, Sousse, Tunis | FSWs with one-time client in past 1 M | Last sex | 54.8 | 45.5 |
| **Yemen** |  |  |  |  |  |  |
| Stulhofer, 2008 [149] | 2008 | Aden | FSWs with one-time client in past 7 days | Last sex | 57.4 | 49.6 |
| **With non-paying partner** |  |  |  |  |  |  |
| **Egypt** |  |  |  |  |  |  |
| MOH, 2006 [129] | 2006 | Cairo | FSWs with non-paying partner | Last sex | 6.8 | NR |
| MOH, 2010 [130] | 2010 | Cairo | FSWs with non-paying partner | Last sex | 11.0 | 5.5 |
| MOH, 2010 [130] | 2010 | Cairo | FSWs with non-paying partner | Past 12 M | 27.4 | NR |
| Kabbash, 2012 [159] | 2009-10 | Greater Cairo | FSWs who heard of condoms and with non-paying partner in past 6 M | Last sex | 13.4 | 10.3^†^ |
| **Iran** |  |  |  |  |  |  |
| Sajadi, 2013 [132] | 2010 | National | FSWs with non-paying partner in past 7 days | Last sex | 36.3 | 28.0 |
| Navadeh, 2012 [131] | 2010 | Kerman | All FSWs | Last sex | 78.3 | NR |
| Kazerooni, 2014 [133] | 2010-11 | Shiraz | All FSWs | Last sex | 45.8 | 27.1^*^ |
| Kazerooni, 2014 [133] | 2010-11 | Shiraz | All FSWs | Past 1 M | 77.4 | NR |
| **Lebanon** |  |  |  |  |  |  |
| Mahfoud, 2010 [137] | 2007-08 | Greater Beirut | FSWs with non-paying partner in past 1 M | Last sex | 48.0 | 64.0 |
| **Pakistan** |  |  |  |  |  |  |
| Bokhari, 2007 [139] | 2004 | Karachi | FSWs with non-paying partner in past 7 days | Last sex | 22.5 | 8.3 |
| Bokhari, 2007 [139] | 2004 | Lahore | FSWs with non-paying partner in past 7 days | Last sex | 21.8 | 8.0 |
| NACP, 2005 [14] | 2004-05 | Karachi | FSWs with non-paying partner | Last sex | 22.2 | NR |
| NACP, 2005 [14] | 2004-05 | Rawalpindi | FSWs with non-paying partner | Last sex | 13.3 | NR |
| NACP, 2005 [14] | 2004-05 | Karachi | FSWs with non-paying partner in past 1 M | Past 1 M | 48.6 | 19.1 |
| NACP, 2005 [14] | 2004-05 | Rawalpindi | FSWs with non-paying partner in past 1 M | Past 1 M | 26.7 | 4.8 |
| Hawkes, 2009 [141] | 2007 | Abbottabad, Rawalpindi | FSWs with non-paying partner | NR | 49.0 | NR |
| Punjab NACP, 2015 [201] | 2014 | Punjab | FSWs with non-paying partner | Past 1 M | NR | 15.1 |
| NACP, 2017 [22] | 2016-17 | National | FSWs with non-paying partner | Last sex | NR | 10.9 |
| **Somalia** |  |  |  |  |  |  |
| Testa, 2008 [143] | 2008 | Hargeisa | FSWs with non-paying partner | Last sex | 4.9 | 8.3 |
| IOM, 2017 [144] | 2014 | Hargeisa | All FSWs | Last sex | 18.8 | 18.7 |
| **Sudan** |  |  |  |  |  |  |
| MOH, 2016 [30] | 2015-16 | Juba, South Sudan | FSWs with non-paying partner | Last sex | 75.0 | 71.0 |
| **Syria** |  |  |  |  |  |  |
| MOH, 2005 [104] | 2005 | NR | FSWs with non-paying partner | NR | 68.6 | 28.2 |
| **Tunisia** |  |  |  |  |  |  |
| MOH, 2010 [203] | 2009 | Sfax, Sousse, Tunis | All FSWs | NR | NR | 19.2 |
| Hsairi, 2012 [31] | 2011 | Sfax, Sousse, Tunis | FSWs with non-paying partner in past 1 M | Last sex | 12.1 | 11.6 |
| **Yemen** |  |  |  |  |  |  |
| Stulhofer, 2008 [149] | 2008 | Aden | FSWs with non-paying partner | Last sex | 28.8 | 25.7 |
| **With regular non-paying partner** | |  |  |  |  |  |
| **Iran** |  |  |  |  |  |  |
| Moayedi-Nia, 2016 [134] | 2012-13 | Tehran | FSWs with a stable partner | NR | 49.0 | NR |
| **Morocco** |  |  |  |  |  |  |
| MOH, 2012 [12] | 2011-12 | Agadir | FSWs with regular partner in past 1 M | Last sex | 20.3 | 48.7^*^ |
| MOH, 2012 [12] | 2011-12 | Fes | FSWs with regular partner in past 1 M | Last sex | 36.9 | 60.8^*^ |
| MOH, 2012 [12] | 2011-12 | Rabat | FSWs with regular partner in past 1 M | Last sex | 23.8 | 82.8^*^ |
| MOH, 2012 [12] | 2011-12 | Tanger | FSWs with regular partner in past 1 M | Last sex | 43.3 | 60.6^*^ |
| **Pakistan** |  |  |  |  |  |  |
| Hawkes, 2009 [141] | 2007 | Abbottabad, Rawalpindi | FSWs with regular non-paying partner | Last sex | 46.0 | 15.0 |
| NACP, 2012 [20] | 2011 | National | FSWs with regular non-paying partner | NR | NR | 20.6 |
| **Sudan** |  |  |  |  |  |  |
| MOH, 2016 [30] | 2015-16 | Juba, South Sudan | FSWs with regular partner in past 6 M | Last sex | NR | 40 |
| **With occasional non-paying partner** | |  |  |  |  |  |
| **Morocco** |  |  |  |  |  |  |
| MOH, 2012 [12] | 2011-12 | Agadir | FSWs with occasional partner in past 1 M | Last sex | 59.0 | 2.7^*^ |
| MOH, 2012 [12] | 2011-12 | Fes | FSWs with occasional partner in past 1 M | Last sex | 43.8 | 46.3^*^ |
| MOH, 2012 [12] | 2011-12 | Rabat | FSWs with occasional partner in past 1 M | Last sex | 64.8 | 50.0^*^ |
| MOH, 2012 [12] | 2011-12 | Tanger | FSWs with occasional partner in past 1 M | Last sex | 80.1 | 64.1^*^ |
| **ANAL SEX** |  |  |  |  |  |  |
| **With clients** |  |  |  |  |  |  |
| **Iran** |  |  |  |  |  |  |
| Kazerooni, 2014 [133] | 2010-11 | Shiraz | All FSWs | Past 1 M | 66.7 | NR |
| **Libya** |  |  |  |  |  |  |
| Valadez, 2013 [138] | 2010-11 | Tripoli | FSWs reporting anal sex in past 1 M | Last sex | 0 | NR |
| **Morocco** |  |  |  |  |  |  |
| MOH, 2012 [12] | 2011-12 | Agadir | FSWs reporting anal sex in past 1 M | Last sex | 52.6 | 63.6^*^ |
| MOH, 2012 [12] | 2011-12 | Fes | FSWs reporting anal sex in past 1 M | Last sex | 35.5 | 55.6^*^ |
| MOH, 2012 [12] | 2011-12 | Rabat | FSWs reporting anal sex in past 1 M | Last sex | 86.5 | 33.3^*^ |
| MOH, 2012 [12] | 2011-12 | Tanger | FSWs reporting anal sex in past 1 M | Last sex | 68.2 | 86.7^*^ |
| **Pakistan** |  |  |  |  |  |  |
| Bokhari, 2007 [139] | 2004 | Karachi | FSWs reporting anal sex with regular client | Last sex | 6.8 | NR |
| Bokhari, 2007 [139] | 2004 | Lahore | FSWs reporting anal sex with regular client | Last sex | 22.3 | NR |
| Bokhari, 2007 [139] | 2004 | Karachi | FSWs reporting anal sex with one-time client | Last sex | 6.7 | NR |
| Bokhari, 2007 [139] | 2004 | Lahore | FSWs reporting anal sex with one-time client | Last sex | 37.5 | NR |
| NACP, 2005 [14] | 2004-05 | Karachi | FSWs reporting anal sex in past 1 M | Last sex | 17.0 | NR |
| NACP, 2005 [14] | 2004-05 | Rawalpindi | FSWs reporting anal sex in past 1 M | Last sex | 17.2 | NR |
| NACP, 2005 [14] | 2005 | Faisalabad | FSWs reporting anal sex | Last sex | 25.0 | NR |
| NACP, 2005 [14] | 2005 | Hyderabad | FSWs reporting anal sex | Last sex | 14.0 | NR |
| NACP, 2005 [14] | 2005 | Karachi | FSWs reporting anal sex | Last sex | 29.0 | NR |
| NACP, 2005 [14] | 2005 | Lahore | FSWs reporting anal sex | Last sex | 55.0 | NR |
| NACP, 2005 [14] | 2005 | Multan | FSWs reporting anal sex | Last sex | 17.0 | NR |
| NACP, 2005 [14] | 2005 | Peshawar | FSWs reporting anal sex | Last sex | 17.0 | NR |
| NACP, 2005 [14] | 2005 | Quetta | FSWs reporting anal sex | Last sex | 14.0 | NR |
| NACP, 2005 [14] | 2005 | Sukkur | FSWs reporting anal sex | Last sex | 35.0 | NR |
| NACP, 2007 [140] | 2006 | National | FSWs reporting anal sex | Last sex | 7.9 | NR |
| Hawkes, 2009 [141] | 2007 | Abbottabad & Rawalpindi | FSWs reporting anal sex | Last sex | 61.0 | NR |
| NACP, 2010 [142] | 2009 | Punjab | FSWs reporting anal sex | Last sex | 5.2 | NR |
| NACP, 2012 [20] | 2011 | Karachi | FSWs reporting anal sex | Last sex | 52.0 | NR |
| NACP, 2012 [20] | 2011 | DG Khan | FSWs reporting anal sex | Last sex | 36.0 | NR |
| NACP, 2012 [20] | 2011 | Faisalabad | FSWs reporting anal sex | Last sex | 46.0 | NR |
| NACP, 2012 [20] | 2011 | Haripur | FSWs reporting anal sex | Last sex | 36.0 | NR |
| NACP, 2012 [20] | 2011 | Lahore | FSWs reporting anal sex | Last sex | 49.0 | NR |
| NACP, 2012 [20] | 2011 | Larkana | FSWs reporting anal sex | Last sex | 13.0 | NR |
| NACP, 2012 [20] | 2011 | Multan | FSWs reporting anal sex | Last sex | 23.0 | NR |
| NACP, 2012 [20] | 2011 | Peshawar | FSWs reporting anal sex | Last sex | 12.0 | NR |
| NACP, 2012 [20] | 2011 | Quetta | FSWs reporting anal sex | Last sex | 56.0 | NR |
| NACP, 2012 [20] | 2011 | Rawalpindi | FSWs reporting anal sex | Last sex | 10.0 | NR |
| NACP, 2012 [20] | 2011 | Sargodha | FSWs reporting anal sex | Last sex | 19.0 | NR |
| NACP, 2012 [20] | 2011 | Sukkur | FSWs reporting anal sex | Last sex | 39.0 | NR |
| Punjab NACP, 2015 [201] | 2014 | Faisalabad | FSWs reporting anal sex in past 1 M | Last sex | 26.2 | NR |
| Punjab NACP, 2015 [201] | 2014 | Lahore | FSWs reporting anal sex in past 1 M | Last sex | 15.2 | NR |
| Punjab NACP, 2015 [201] | 2014 | Multan | FSWs reporting anal sex in past 1 M | Last sex | 16.0 | NR |
| Punjab NACP, 2015 [201] | 2014 | Sargodha | FSWs reporting anal sex in past 1 M | Last sex | 18.9 | NR |
| NACP, 2017 [22] | 2016-17 | Bannu | FSWs reporting anal sex | Last sex | 60.2 | NR |
| NACP, 2017 [22] | 2016-17 | Bahawalpur | FSWs reporting anal sex | Last sex | 11.9 | NR |
| NACP, 2017 [22] | 2016-17 | DG Khan | FSWs reporting anal sex | Last sex | 4.9 | NR |
| NACP, 2017 [22] | 2016-17 | Gujranwala | FSWs reporting anal sex | Last sex | 19.7 | NR |
| NACP, 2017 [22] | 2016-17 | Gujrat | FSWs reporting anal sex | Last sex | 24.6 | NR |
| NACP, 2017 [22] | 2016-17 | Hyderabad | FSWs reporting anal sex | Last sex | 30.8 | NR |
| NACP, 2017 [22] | 2016-17 | Karachi | FSWs reporting anal sex | Last sex | 4.1 | NR |
| NACP, 2017 [22] | 2016-17 | Kasur | FSWs reporting anal sex | Last sex | 10.4 | NR |
| NACP, 2017 [22] | 2016-17 | Larkana | FSWs reporting anal sex | Last sex | 1.6 | NR |
| NACP, 2017 [22] | 2016-17 | Mirpurkhas | FSWs reporting anal sex | Last sex | 8.5 | NR |
| NACP, 2017 [22] | 2016-17 | Nawabshah | FSWs reporting anal sex | Last sex | 1.4 | NR |
| NACP, 2017 [22] | 2016-17 | Peshawar | FSWs reporting anal sex | Last sex | 13.2 | NR |
| NACP, 2017 [22] | 2016-17 | Quetta | FSWs reporting anal sex | Last sex | 42.9 | NR |
| NACP, 2017 [22] | 2016-17 | Rawalpindi | FSWs reporting anal sex | Last sex | 0 | NR |
| NACP, 2017 [22] | 2016-17 | Sheikhupura | FSWs reporting anal sex | Last sex | 27.5 | NR |
| NACP, 2017 [22] | 2016-17 | Sialkot | FSWs reporting anal sex | Last sex | 6.2 | NR |
| NACP, 2017 [22] | 2016-17 | Sukkur | FSWs reporting anal sex | Last sex | 18.1 | NR |
| NACP, 2017 [22] | 2016-17 | Turbat | FSWs reporting anal sex | Last sex | 6.9 | NR |
| **With non-paying partner** |  |  |  |  |  |  |
| **Iran** |  |  |  |  |  |  |
| Kazerooni, 2014 [133] | 2010-11 | Shiraz | FSWs reporting anal sex | Past 1 M | 39.0 | NR |
| **CLIENTS OF FSWS** |  |  |  |  |  |  |
| **Afghanistan** |  |  |  |  |  |  |
| Todd, 2012 [123] | 2010-11 | National | Army recruits ever clients of FSWs | Last sex | 17.9 | 9.3 |
| **Djibouti** |  |  |  |  |  |  |
| Trellu-Kane, 2005 [7] | 2005 | Djibotui | Men aged 13-24 years clients of FSWs in past 12 M | Last sex | 53.0 | NR |
| **Morocco** |  |  |  |  |  |  |
| MOH, 2007 [125] | 2007 | National | Men aged 15-24 ever clients of FSWs | Ever | 77.2 | 35.0 |
| MOH, 2013 [120] | 2013 | National | Men aged 15-24 years clients of FSWs in past 12 M | Past 12 M | 90.4 | 45.2 |
| **Pakistan** |  |  |  |  |  |  |
| Bokhari, 2007 [139] | 2004 | Karachi | Truck drivers clients of FSWs in past 12 M | Last sex | 1.7 | NR |
| Bokhari, 2007 [139] | 2004 | Lahore | Truck drivers clients of FSWs in past 12 M | Last sex | 6.9 | NR |
| Faisel, 2005 [39] | 2004-05 | Lahore | Migrant men clients of FSWs in past 12 M | Last sex | 10.0 | 15.0^*^ |
| Mir, 2013 [126] | 2007 | National | Men clients of FSWs in past 12 M | Past 12 M | 33.1 | 17.3 |
| **Sudan** |  |  |  |  |  |  |
| UNHCR, 2007 [27] | 2006 | Juba, South Sudan | Men clients of FSWs in past 12 M | Last sex | 0 | NR |

The table is sorted by year(s) of data collection.

^*^Consistent condom use among FSWs who reported condom use with client/partner.

^†^Consistent condom use among FSWs who ever heard of condoms.

*Abbreviations*: *CI* confidence interval, *FSWs* female sex workers, *IOM* International Organization for Migration, *M* month(s), *MOH* Ministry of Health, *NACP* National AIDS Control Programme, *NAP* National AIDS Program, *NR* not reported, *SAR AIDS HDS* South Asia Region AIDS Human Development Sector, *STI* sexually transmitted infections, *UNHCR* United Nations High Commissioner for Refugees

**Table S12** Measures of injecting drug use and overlap with people who inject drugs (PWID) among FSWs in the Middle East and North Africa

| **Country**  **Author, year [citation]** | **Year(s) of data collection** | **City/**  **province** | **Drug use** | | | **Injecting drug use** | | | **Sex with PWID** | | |
| --- | --- | --- | --- | --- | --- | --- | --- | --- | --- | --- | --- |
|  |  |  | **Pop** | **Time frame** | **Proportion (%)** | **Pop** | **Time frame** | **Proportion (%)** | **Pop** | **Time frame** | **Proportion (%)** |
| **FSWS** |  |  |  |  |  |  |  |  |  |  |  |
| **Afghanistan** |  |  |  |  |  |  |  |  |  |  |  |
| Todd, 2010 [151] | 2006-08 | Jalalabad, Kabul, Mazar-i-Sharif | All FSWs | Ever | 6.9 | All FSWs | Ever | 0.4 | NR | NR | NR |
| NACP, 2010 [128] | 2009 | Kabul | All FSWs | Ever | 1.9 | All FSWs | Ever | 0 | All FSWs | Past 1 M | 0.5 |
| NACP, 2012 [6] | 2012 | Kabul | All FSWs | Ever | 1.7 | All FSWs | Ever | 0.1 | All FSWs | Past 12 M | 3.8 |
| NACP, 2012 [6] | 2012 | Herat | All FSWs | Ever | 11.7 | All FSWs | Ever | 7.1 | All FSWs | Past 12 M | 13.6 |
| NACP, 2012 [6] | 2012 | Mazar-i-Sharif | All FSWs | Ever | 5.5 | All FSWs | Ever | 0 | All FSWs | Past 12 M | 6.5 |
| **Egypt** |  |  |  |  |  |  |  |  |  |  |  |
| MOH, 2006 [129] | 2006 | Cairo | All FSWs | Ever | 78.8 | All FSWs | Past 12 M | 9.3 | NR | NR | NR |
| Kabbash, 2012 [159] | 2009-10 | Cairo | All FSWs | Ever | 49.0 | All FSWs | Past 12 M | 5.6 | NR | NR | NR |
| MOH, 2010 [130] | 2010 | Cairo | All FSWs | Ever | 51.5 | All FSWs | Past 12 M | 6.0 | NR | NR | NR |
| **Iran** |  |  |  |  |  |  |  |  |  |  |  |
| Kassaian, 2012 [161] | 2009-10 | Isfahan | All FSWs | Ever | 61.3 | All FSWs | NR | 19.0 | NR | NR | NR |
| Kassaian, 2012 [161] | 2009-10 | Isfahan | NR | NR | NR | Ever DU | Ever | 24.1 | NR | NR | NR |
| Sajadi, 2013 [132] | 2010 | National | All FSWs | Ever | 73.8 | Ever DU | Ever | 20.5 | NR | NR | NR |
| Sajadi, 2013 [132] | 2010 | National | NR | NR | NR | Ever IDU | Active IDU | 26.6 | NR | NR | NR |
| Mirzazadeh, 2016 [135] | 2010 | National | NR | NR | NR | All FSWs | Ever | 13.6 | NR | NR | NR |
| Navadeh, 2012 [131] | 2010 | Kerman | NR | NR | NR | All FSWs | Ever | 18.0 | NR | NR | NR |
| Kazerooni, 2014 [133] | 2010-11 | Shiraz | All FSWs | Ever | 69.9 | Ever DU | Ever | 16.4 | NR | NR | NR |
| Moayedi-Nia, 2016 [134] | 2012-13 | Tehran | All FSWs | Ever | 90.7 | NR | NR | NR | NR | NR | NR |
| Moayedi-Nia, 2016 [134] | 2012-13 | Tehran | Ever DU | Current | 50.9 | Active DU | Ever | 25.5 | NR | NR | NR |
| Taghizadeh, 2015 [162] | 2014 | Sari | All FSWs | Current | 59.0 | Active DU | Current | 1.1 | NR | NR | NR |
| Asadi-Ali, 2018 [163] | 2015 | Northern Iran | All FSWs | Past 12 M | 39.7 | All FSWs | NR | NR | NR | NR | NR |
| Mirzazadeh, 2016 [135] | 2015 | National | All FSWs | Ever | 59.8 | All FSWs | Ever | 6.1 | NR | NR | NR |
| Karami, 2017 [11] | 2016 | Tehran | NR | NR | NR | NR | NR | NR | All FSWs | NR | 23.6 |
| **Lebanon** |  |  |  |  |  |  |  |  |  |  |  |
| Naman, 1989 [164] | 1985-87 | NR | NR | NR | NR | All FSWs | NR | 1.4 | NR | NR | NR |
| Mahfoud, 2010 [137] | 2007-08 | Beirut | NR | NR | NR | All FSWs | Ever | 0 | NR | NR | NR |
| **Libya** |  |  |  |  |  |  |  |  |  |  |  |
| Valadez, 2013 [138] | 2010-11 | Tripoli | All FSWs | Past 6 M | 1.2 | All FSWs | Ever | 0 | NR | NR | NR |
| **Morocco** |  |  |  |  |  |  |  |  |  |  |  |
| MOH, 2012 [12] | 2011-12 | Agadir | All FSWs | Ever | 13.2 | Ever DU | Ever | 0.3 | NR | NR | NR |
| MOH, 2012 [12] | 2011-12 | Fes | All FSWs | Ever | 17.7 | Ever DU | Ever | 6.8 | NR | NR | NR |
| MOH, 2012 [12] | 2011-12 | Rabat | All FSWs | Ever | 8.1 | Ever DU | Ever | 0 | NR | NR | NR |
| MOH, 2012 [12] | 2011-12 | Tanger | All FSWs | Ever | 7.9 | Ever DU | Ever | 11.8 | NR | NR | NR |
| MOH, 2012 [12] | 2011-12 | Agadir | Ever DU | Past 6 M | 81.6 | NR | NR | NR | NR | NR | NR |
| MOH, 2012 [12] | 2011-12 | Fes | Ever DU | Past 6 M | 95.0 | NR | NR | NR | NR | NR | NR |
| MOH, 2012 [12] | 2011-12 | Rabat | Ever DU | Past 6 M | 85.8 | NR | NR | NR | NR | NR | NR |
| MOH, 2012 [12] | 2011-12 | Tanger | Ever DU | Past 6 M | 79.4 | NR | NR | NR | NR | NR | NR |
| **Pakistan** |  |  |  |  |  |  |  |  |  |  |  |
| Baqi, 1998 [167] | 1993-94 | Karachi | All FSWs | Current | 1.2 | All FSWs | Ever | 0 | NR | NR | NR |
| Bokhari, 2007 [139] & NACP, 2005 [14] | 2004 | Karachi | NR | NR | NR | All FSWs | Past 12 M | 4.4 | All FSWs | NR | 18.2 |
| Bokhari, 2007 [139] & NACP, 2005 [14] | 2004 | Lahore | NR | NR | NR | All FSWs | Past 12 M | 1.2 | All FSWs | NR | 22.8 |
| NACP, 2005 [14] | 2004-05 | Karachi | All FSWs | Current | 23.1 | All FSWs | Current | 4.6 | NR | NR | NR |
| NACP, 2005 [14] | 2004-05 | Rawalpindi | All FSWs | Current | 8.9 | All FSWs | Current | 0 | NR | NR | NR |
| NACP, 2005 [14] | 2005 | Faisalabad | NR | NR | NR | All FSWs | Past 6 M | 8.0 | All FSWs | Past 6 M | 33.0 |
| NACP, 2005 [14] | 2005 | Hyderabad | NR | NR | NR | All FSWs | Past 6 M | 0 | All FSWs | Past 6 M | 5.0 |
| NACP, 2005 [14] | 2005 | Karachi | NR | NR | NR | All FSWs | Past 6 M | 1.0 | All FSWs | Past 6 M | 3.0 |
| NACP, 2005 [14] | 2005 | Lahore | NR | NR | NR | All FSWs | Past 6 M | 2.5 | All FSWs | Past 6 M | 19.0 |
| NACP, 2005 [14] | 2005 | Multan | NR | NR | NR | All FSWs | Past 6 M | 3.0 | All FSWs | Past 6 M | 8.0 |
| NACP, 2005 [14] | 2005 | Peshawar | NR | NR | NR | All FSWs | Past 6 M | 0 | All FSWs | Past 6 M | 17.0 |
| NACP, 2005 [14] | 2005 | Quetta | NR | NR | NR | All FSWs | Past 6 M | 5.0 | All FSWs | Past 6 M | 15.0 |
| NACP, 2005 [14] | 2005 | Sukkur | NR | NR | NR | All FSWs | Past 6 M | 8.0 | All FSWs | Past 6 M | 8.0 |
| NACP, 2007 [140] | 2006 | Bannu | NR | NR | NR | All FSWs | Past 6 M | 3.2 | All FSWs | Past 6 M | 6.8 |
| NACP, 2007 [140] | 2006 | Faisalabad | NR | NR | NR | All FSWs | Past 6 M | 7.5 | All FSWs | Past 6 M | 31.0 |
| NACP, 2007 [140] | 2006 | Gujranwala | NR | NR | NR | All FSWs | Past 6 M | 5.3 | All FSWs | Past 6 M | 30.3 |
| NACP, 2007 [140] | 2006 | Hyderabad | NR | NR | NR | All FSWs | Past 6 M | 3.3 | All FSWs | Past 6 M | 2.3 |
| NACP, 2007 [140] | 2006 | Karachi | NR | NR | NR | All FSWs | Past 6 M | 0.7 | All FSWs | Past 6 M | 4.2 |
| NACP, 2007 [140] | 2006 | Lahore | NR | NR | NR | All FSWs | Past 6 M | 1.6 | All FSWs | Past 6 M | 16.9 |
| NACP, 2007 [140] | 2006 | Larkana | NR | NR | NR | All FSWs | Past 6 M | 1.0 | All FSWs | Past 6 M | 0.3 |
| NACP, 2007 [140] | 2006 | Multan | NR | NR | NR | All FSWs | Past 6 M | 1.0 | All FSWs | Past 6 M | 2.3 |
| NACP, 2007 [140] | 2006 | Peshawar | NR | NR | NR | All FSWs | Past 6 M | 1.7 | All FSWs | Past 6 M | 6.7 |
| NACP, 2007 [140] | 2006 | Quetta | NR | NR | NR | All FSWs | Past 6 M | 1.5 | All FSWs | Past 6 M | 3.3 |
| NACP, 2007 [140] | 2006 | Sargodha | NR | NR | NR | All FSWs | Past 6 M | 1.3 | All FSWs | Past 6 M | 12.5 |
| NACP, 2007 [140] | 2006 | Sukkur | NR | NR | NR | All FSWs | Past 6 M | 0 | All FSWs | Past 6 M | 0 |
| Hawkes, 2009 [141] | 2007 | Abbottabad, Rawalpindi | NR | NR | NR | All FSWs | Past 12 M | 3.0 | All FSWs | Past 12 M | 36.0 |
| Khan, 2011 [17] | 2007 | Lahore | NR | NR | NR | All FSWs | NR | 0.4 | NR | NR | NR |
| NACP, 2010 [142] | 2009 | Punjab | NR | NR | NR | All FSWs | Past 6 M | 6.0 | All FSWs | Past 6 M | 7.0 |
| NACP, 2012 [20] | 2011 | DG Khan | NR | NR | NR | All FSWs | Past 6 M | 5.1 | All FSWs | Past 6 M | 1.1 |
| NACP, 2012 [20] | 2011 | Faisalabad | NR | NR | NR | All FSWs | Past 6 M | 6.4 | All FSWs | Past 6 M | 13.8 |
| NACP, 2012 [20] | 2011 | Haripur | NR | NR | NR | All FSWs | Past 6 M | 2.4 | All FSWs | Past 6 M | 1.9 |
| NACP, 2012 [20] | 2011 | Karachi | NR | NR | NR | All FSWs | Past 6 M | 1.9 | All FSWs | Past 6 M | 5.6 |
| NACP, 2012 [20] | 2011 | Lahore | NR | NR | NR | All FSWs | Past 6 M | 5.1 | All FSWs | Past 6 M | 7.2 |
| NACP, 2012 [20] | 2011 | Larkana | NR | NR | NR | All FSWs | Past 6 M | 0.3 | All FSWs | Past 6 M | 0.5 |
| NACP, 2012 [20] | 2011 | Multan | NR | NR | NR | All FSWs | Past 6 M | 16.8 | All FSWs | Past 6 M | 24.8 |
| NACP, 2012 [20] | 2011 | Peshawar | NR | NR | NR | All FSWs | Past 6 M | 0 | All FSWs | Past 6 M | 0.3 |
| NACP, 2012 [20] | 2011 | Quetta | NR | NR | NR | All FSWs | Past 6 M | 6.7 | All FSWs | Past 6 M | 30.3 |
| NACP, 2012 [20] | 2011 | Rawalpindi | NR | NR | NR | All FSWs | Past 6 M | 1.3 | All FSWs | Past 6 M | 2.1 |
| NACP, 2012 [20] | 2011 | Sargodha | NR | NR | NR | All FSWs | Past 6 M | 5.2 | All FSWs | Past 6 M | 23.2 |
| NACP, 2012 [20] | 2011 | Sukkur | NR | NR | NR | All FSWs | Past 6 M | 6.1 | All FSWs | Past 6 M | 39.7 |
| PNACP, 2015 [201] | 2014 | Faisalabad | NR | NR | NR | All FSWs | Past 6 M | 1.4 | All FSWs | Past 6 M | 0.5 |
| PNACP, 2015 [201] | 2014 | Lahore | NR | NR | NR | All FSWs | Past 6 M | 1.0 | All FSWs | Past 6 M | 3.1 |
| PNACP, 2015 [201] | 2014 | Multan | NR | NR | NR | All FSWs | Past 6 M | 4.0 | All FSWs | Past 6 M | 3.8 |
| PNACP, 2015 [201] | 2014 | Sargodha | NR | NR | NR | All FSWs | Past 6 M | 2.1 | All FSWs | Past 6 M | 2.6 |
| NACP, 2017 [22] | 2016-17 | Bahawalpur | NR | NR | NR | All FSWs | Past 12 M | 1.1 | All FSWs | Past 12 M | 0.3 |
| NACP, 2017 [22] | 2016-17 | Bannu | NR | NR | NR | All FSWs | Past 12 M | 0 | All FSWs | Past 12 M | 0.5 |
| NACP, 2017 [22] | 2016-17 | DG Khan | NR | NR | NR | All FSWs | Past 12 M | 0.3 | All FSWs | Past 12 M | 0 |
| NACP, 2017 [22] | 2016-17 | Gujranwala | NR | NR | NR | All FSWs | Past 12 M | 0.7 | All FSWs | Past 12 M | 0.3 |
| NACP, 2017 [22] | 2016-17 | Gujrat | NR | NR | NR | All FSWs | Past 12 M | 5.6 | All FSWs | Past 12 M | 19.4 |
| NACP, 2017 [22] | 2016-17 | Hyderabad | NR | NR | NR | All FSWs | Past 12 M | 10.4 | All FSWs | Past 12 M | 25.5 |
| NACP, 2017 [22] | 2016-17 | Karachi | NR | NR | NR | All FSWs | Past 12 M | 0 | All FSWs | Past 12 M | 3.4 |
| NACP, 2017 [22] | 2016-17 | Kasur | NR | NR | NR | All FSWs | Past 12 M | 0.3 | All FSWs | Past 12 M | 5.5 |
| NACP, 2017 [22] | 2016-17 | Larkana | NR | NR | NR | All FSWs | Past 12 M | 0.5 | All FSWs | Past 12 M | 0.5 |
| NACP, 2017 [22] | 2016-17 | Mirpurkhas | NR | NR | NR | All FSWs | Past 12 M | 0.5 | All FSWs | Past 12 M | 4.9 |
| NACP, 2017 [22] | 2016-17 | Nawabshah | NR | NR | NR | All FSWs | Past 12 M | 9.3 | All FSWs | Past 12 M | 3.8 |
| NACP, 2017 [22] | 2016-17 | Peshawar | NR | NR | NR | All FSWs | Past 12 M | 1.1 | All FSWs | Past 12 M | 14.0 |
| NACP, 2017 [22] | 2016-17 | Quetta | NR | NR | NR | All FSWs | Past 12 M | 9.3 | All FSWs | Past 12 M | 54.9 |
| NACP, 2017 [22] | 2016-17 | Rawalpindi | NR | NR | NR | All FSWs | Past 12 M | 0.3 | All FSWs | Past 12 M | 4.9 |
| NACP, 2017 [22] | 2016-17 | Sheikhupura | NR | NR | NR | All FSWs | Past 12 M | 5.5 | All FSWs | Past 12 M | 45.2 |
| NACP, 2017 [22] | 2016-17 | Sialkot | NR | NR | NR | All FSWs | Past 12 M | 0 | All FSWs | Past 12 M | 0 |
| NACP, 2017 [22] | 2016-17 | Sukkur | NR | NR | NR | All FSWs | Past 12 M | 5.5 | All FSWs | Past 12 M | 16.5 |
| NACP, 2017 [22] | 2016-17 | Turbat | NR | NR | NR | All FSWs | Past 12 M | 2.8 | All FSWs | Past 12 M | 25.0 |
| **Somalia** |  |  |  |  |  |  |  |  |  |  |  |
| Burans, 1990 [174] | NR | Mogadishu | All FSWs | Current | 13.5 | All FSWs | NR | 0 | NR | NR | NR |
| Testa, 2008 [143] | 2008 | Hargeisa | All FSWs | Past 1 M | 0.6 | All FSWs | Past 12 M | 0 | NR | NR | NR |
| IOM, 2017 [144] | 2014 | Hargeisa | All FSWs | Ever | 85.2 | All FSWs | Past 12 M | 0.6 | NR | NR | NR |
| IOM, 2017 [144] | 2014 | Hargeisa | All FSWs | Past 1 M | 4.7 | NR | NR | NR | NR | NR | NR |
| **Sudan** |  |  |  |  |  |  |  |  |  |  |  |
| Elhadi, 2013 [202] | 2011 | Alshamalia | NR | NR | NR | All FSWs | Ever | 1.5 | NR | NR | NR |
| Elhadi, 2013 [202] | 2011 | Blue Nile | NR | NR | NR | All FSWs | Ever | 0.9 | NR | NR | NR |
| Elhadi, 2013 [202] | 2011 | Gadarif | NR | NR | NR | All FSWs | Ever | 0.5 | NR | NR | NR |
| Elhadi, 2013 [202] | 2011 | Gezira | NR | NR | NR | All FSWs | Ever | 0.4 | NR | NR | NR |
| Elhadi, 2013 [202] | 2011 | Kassala | NR | NR | NR | All FSWs | Ever | 0.9 | NR | NR | NR |
| Elhadi, 2013 [202] | 2011 | Khartoum | NR | NR | NR | All FSWs | Ever | 2.3 | NR | NR | NR |
| Elhadi, 2013 [202] | 2011 | North Darfur | NR | NR | NR | All FSWs | Ever | 5.0 | NR | NR | NR |
| Elhadi, 2013 [202] | 2011 | North Kodofan | NR | NR | NR | All FSWs | Ever | 0.1 | NR | NR | NR |
| Elhadi, 2013 [202] | 2011 | Red Sea | NR | NR | NR | All FSWs | Ever | 0 | NR | NR | NR |
| Elhadi, 2013 [202] | 2011 | River Nile | NR | NR | NR | All FSWs | Ever | 0.6 | NR | NR | NR |
| Elhadi, 2013 [202] | 2011 | Sinnar | NR | NR | NR | All FSWs | Ever | 1.0 | NR | NR | NR |
| Elhadi, 2013 [202] | 2011 | South Darfur | NR | NR | NR | All FSWs | Ever | 2.6 | NR | NR | NR |
| Elhadi, 2013 [202] | 2011 | West Darfur | NR | NR | NR | All FSWs | Ever | 1.6 | NR | NR | NR |
| Elhadi, 2013 [202] | 2011 | White Nile | NR | NR | NR | All FSWs | Ever | 1.6 | NR | NR | NR |
| MOH, 2016 [30] | 2015-16 | Juba, South Sudan | All FSWs | Past 6 M | 14.0 | NR | NR | NR | NR | NR | NR |
| **Syria** |  |  |  |  |  |  |  |  |  |  |  |
| MOH, 2005 [104] | 2005 | NR | NR | NR | NR | All FSWs | Ever | 10.0 | NR | NR | NR |
| **Tunisia** |  |  |  |  |  |  |  |  |  |  |  |
| MOH, 2010 [203] | 2009 | Sfax, Sousse, Tunis | All FSWs | Ever | 31.3 | NR | NR | NR | NR | NR | NR |
| Hsairi, 2012 [31] | 2011 | Sfax | All FSWs | Ever | 29.2 | Ever DU | Past 12 M | 0 | NR | NR | NR |
| Hsairi, 2012 [31] | 2011 | Sousse | All FSWs | Ever | 24.8 | Ever DU | Past 12 M | 4.7 | NR | NR | NR |
| Hsairi, 2012 [31] | 2011 | Tunis | All FSWs | Ever | 18.8 | Ever DU | Past 12 M | 8.8 | NR | NR | NR |
| **Yemen** |  |  |  |  |  |  |  |  |  |  |  |
| Stulhofer, 2008 [149] | 2008 | Aden | All FSWs | Past 1 M | 2.4 | All FSWs | Past 1 M | 2.1 | NR | NR | NR |
| **CLIENTS OF FSWS** |  |  |  |  |  |  |  |  |  |  |  |
| **Afghanistan** |  |  |  |  |  |  |  |  |  |  |  |
| Todd, 2012 [123] | 2010-11 | National | Army recruits-clients | Ever | 32.9 | NR | NR | NR | NR | NR | NR |
| **Somalia** |  |  |  |  |  |  |  |  |  |  |  |
| Burans, 1990 [174] | NR | Mogadishu | NR | NR | NR | STI clinic attendees | NR | 0 | NA | NA | NA |
| Rehan, 2003 [193] | 1999 | Lahore, Karachi, Peshawar, Quetta | STI clinic attendees | NR | 10.5 | NR | NR | NR | NA | NA | NA |

The table is sorted by year(s) of data collection.

*Abbreviations*: *DU* drug users, *FSWs* female sex workers, *IDU* injecting drug users, *IOM* International Organization for Migration, *M* month(s), *MOH* Ministry of Health, *NA* not applicable, *NACP* National AIDS Control Programme, *NR* not reported, *PNACP* Punjab National AIDS Control Programme, *Prp* proportion, *PWID* people who inject drugs

**Table S13** HIV/AIDS knowledge among FSWs in the Middle East and North Africa

|  | **Afghanistan** | **Egypt** | **Iran** | **Lebanon** | **Morocco** | **Pakistan** | **Somalia** | **Sudan** | **Syria** | **Tunisia** | **Yemen** |
| --- | --- | --- | --- | --- | --- | --- | --- | --- | --- | --- | --- |
| **Aware of HIV/AIDS** |  |  |  |  |  |  |  |  |  |  |  |
| Ever heard of HIV/AIDS (%) | 25.4 [6], 32.4 [128], 37.8 [6], 39.9 [6], 54.0 [5], 75.0 [5] | 100.0 [129], 89.0 [130] | 92.7 [132], 98.7 [162] |  | 84.3 [12], 99.0 [12], 99.6 [12], 100.0 [12] | 35.0 [141], 64.1 [15], 68.4 [139], 66.9 [22], 68.7 [140], 75.2 [139], 80.3 [14], 80.4 [20], 80.7 [14], 83.0 [17], 87.3 [201] | 64.9 [176], 96.2 [143], 97.3 [144] | 98.4 [146] | 97.5 [204] | 94.2 [31], 95.0 [203] |  |
| **Aware of sex as a mode of HIV transmission** | | | | | | | | | | | |
| In all FSWs (%) | 59.0 [5], 72.0 [5] |  |  |  | 72.0 [86] | 50.8 [22], 63.8 [14], 68.6 [14] |  | 78.5 [30], 85.4 [146] | 94.9 [204] |  |  |
| In FSWs who ever heard of HIV (%) |  |  |  |  |  | 68.9 [140], 70.2 [140], 71.9 [140], 74.0 [140], 75.5 [140], 75.9 [140], 81.7 [15], 84.6 [140], 84.8 [201], 86.5 [140], 86.6 [140], 87.1 [140], 87.3 [140], 93.7 [140], 94.3 [20] |  |  |  |  |  |
| **Aware of HIV transmission through unprotected sex** | | | | | | | | | | | |
| In all FSWs (%) | 14.1 [128], 24.2 [6], 32.0 [5], 34.7 [6], 34.8 [6], 47.0 [5] |  | 89.8 [132] | 88.0 [137] | 50.6 [12], 58.4 [12], 59.8 [12], 61.0 [12], 72.0 [86] | 15.4 [14], 26.0 [139], 39.7 [142], 45.3 [14], 46.8 [22], 54.5 [139], 75.8 [20] | 51.6 [144], 70.6 [143] | 57.9 [30] | 76.6 [204] |  | 77.9 [149] |
| In FSWs who ever heard of HIV (%) |  | 49.4 [130] |  |  |  | 38.6 [140], 44.0 [140], 44.9 [140], 47.6 [140], 47.8 [140], 60.4 [15], 68.5 [140], 71.7 [140], 72.9 [140], 73.2 [20], 78.2 [140], 78.8 [140], 81.2 [140], 86.3 [201], 86.8 [140] |  |  |  | 66.1 [31], 78.9 [203], 83.1 [31], 86.7 [31] |  |
| **Aware of sharing needles as a mode of HIV transmission** | | | | | | | | | | | |
| In all FSWs (%) | 30.7 [128] |  | 95.4 [132] | 91.0 [137] | 84.9 [12], 93.4 [12], 95.3 [12], 99.6 [12] | 11.5 [14], 18.9 [22], 57.0[14], 63.3 [139], 72.1 [139] | 95.8 [143], 99.5 [144] | 91.3 [146] | 86.4 [104] |  |  |
| In FSWs who ever heard of HIV (%) |  | 88.2 [130] |  |  |  | 32.6 [20], 37.3 [140], 42.4 [15], 67.1 [201] |  |  |  | 92.4 [31], 92.9 [31], 96.4 [31] |  |

*Abbreviations:* *FSWs* female sex workers

**Table S14** Perception of risk among FSWs in the Middle East and North Africa

| **Perception of being at risk of HIV** | **Iran** | **Lebanon** | **Pakistan** | **Sudan** | **Syria** | **Yemen** |
| --- | --- | --- | --- | --- | --- | --- |
| No risk (%) |  |  |  | 4.9 [148], 7.0 [200], 11.2 [30], 12.5 [148], 14.3 [148], 15.1 [148], 15.9 [146], 21.4 [148], 22.7 [148], 23.7 [148], 25.8 [148], 26.9 [148], 27.6 [148], 29.0 [200], 34.2 [148], 35.8 [148], 37.8 [148], 44.4 [148] | 16.1 [204] |  |
| At risk |  |  |  |  |  |  |
| Among all FSWs (%) | 48.5 [132] | 44.0 [137] | 22.8 [22], 23.0 [14], 25.2 [14], 45.0 [17] |  |  |  |
| Among FSWs who ever heard of HIV (%) |  |  | 28.0 [15], 38.0 [140], 45.1 [20], 65.9 [201] |  |  |  |
| Low risk (%) |  |  |  | 7.1 [148], 8.6 [148], 8.8 [148], 11.6 [148], 12.1 [148], 12.1 [148], 12.4 [148], 13.7 [148], 18.3 [148], 19.8 [148], 24.1 [148], 27.0 [146], 27.3 [148], 31.5 [148], 32.1 [148], 46.9 [30] | 46.2 [104] |  |
| Medium risk (%) |  |  |  | 5.3 [148], 5.5 [148], 9.1 [148], 10.6 [148], 11.2 [148], 11.4 [148], 15.3 [148], 16.3 [148], 16.4 [148], 19.9 [148], 20.2 [148], 22.9 [148], 23.5 [148], 27.3 [30], 32.9 [148], 36.1 [146] |  |  |
| High risk (%) |  |  |  | 5.9 [148], 6.6 [148], 7.4 [148], 8.6 [148], 9.6 [148], 10.9 [148], 13.8 [148], 14.3 [148], 14.5 [148], 14.6 [30], 15.5 [148], 15.8[148], 20.7 [148], 21.0 [146], 21.4 [148], 32.0 [148] | 18.7 [204] | 14.1 [149] |

*Abbreviations*: *FSWs* female sex workers

**Table S15** HIV testing among FSWs in the Middle East and North Africa

| **HIV testing** | **Afg** | **Alg** | **Egypt** | **Iran** | **Leb** | **Lib** | **Mor** | **Pakistan** | **Somal** | **Sudan** | **Syria** | **Tunisia** | **Yemen** |
| --- | --- | --- | --- | --- | --- | --- | --- | --- | --- | --- | --- | --- | --- |
| **Ever tested** |  |  |  |  |  |  |  |  |  |  |  |  |  |
| Ever tested among all FSWs (%) | 4.0 [5], 4.3 [128], 12.0 [5], 21.7 [6], 93.2 [6], 96.2 [6] |  |  | 45.0 [205], 80.6 [205], 99.4 [162] | 79.0 [137] |  | 24.3 [12], 33.5 [12], 34.8 [12], 36.0 [12] | 4.9 [14], 6.0 [141], 8.5 [14], 17.2 [22] | 5.0 [143], 29.6 [144] | 4.4 [148], 5.2 [148], 5.4 [148], 8.0 [148], 8.6 [148], 9.4 [148], 10.4 [148], 12.2 [148], 14.4 [148], 14.6 [148], 17.6 [148], 17.9 [148], 22.0 [148], 23.9 [148], 78.7 [30] | 45.0 [104] |  | 20.1 [149] |
| Ever tested among FSWs who ever heard of HIV (%) |  |  | 3.4 [130] |  |  |  |  | 0.5 [140], 0.5 [140], 1.5 [140], 2.8 [140], 2.8 [140], 3.3 [140], 4.1 [140], 6.2 [15], 8.3 [140], 8.5 [140], 14.4 [140], 15.7 [20], 15.8 [140], 16.5 [140], 55.9 [201] |  |  |  | 21.8 [31], 27.7 [31], 38.0 [31], 15.5 [203] |  |
| Ever received results among FSWs who ever tested for HIV (%) | 78.6 [6], 81.0 [6], 96.9 [6] |  |  |  | 99.0 [137] |  | 91.9 [12], 95.5 [12], 96.0 [12], 96.7 [12] | 60.0 [201] |  |  | 75.8 [104] | 87.2 [31] |  |
| Ever tested and received results among all FSWs (%) |  |  |  |  |  |  |  | 0.7 [139], 0.9 [139] | 4.0 [143] |  |  | 8.8 [203] |  |
| **Tested in past 12 M** |  |  |  |  |  |  |  |  |  |  |  |  |  |
| Tested in past 12 months among all FSWs (%) |  |  |  | 35.9 [206] |  |  | 13.4 [12], 17.9 [12], 20.3 [12], 25.3 [12] |  |  | 0.9 [148], 2.5 [148], 3.1 [148], 4.5 [148], 5.2 [148], 6.2 [148], 8.1 [148], 8.5 [148], 9.6 [148], 11.1 [148], 12.1 [148], 12.4 [148], 12.7 [148], 19.1 [148] | 38.0 [204] | 14.3 [31] |  |
| Tested in past 12 months among FSWs who ever tested for HIV (%) | 43.1 [6], 57.1 [6], 75.0 [6] |  | 33.3 [130] |  | 82.0 [137] |  | 58.9 [12], 59.4 [12], 65.1 [12],  71.7 [12] |  | 47.7 [143], 77.2 [144] |  |  |  | 38.9 [149] |
| Received results in past 12 M among all FSWs (%) |  |  |  |  |  |  |  |  |  | 0.4 [148], 1.7 [148], 2.4 [148], 4.1 [148], 5.4 [148], 6.0 [148], 7.8 [148], 8.3 [148], 9.2 [148], 10.0 [148], 10.8 [148], 11.5 [148], 11.6 [148], 18.4 [148] |  |  |  |
| Received results among FSWs who tested for HIV in past 12 M (%) |  |  |  | 79.0 [206] |  |  |  |  | 86.7 [144], 100.0 [143] | 38.5 [148], 51.8 [148], 86.0 [148], 89.8 [148], 91.6 [148], 93.3 [148], 93.5 [148], 93.5 [148], 93.8 [148], 93.9 [148], 96.0 [148]¸96.4 [148], 99.3 [148], 100.0 [148] |  |  |  |
| Tested & received results in past 12 M among all FSWs (%) |  | 20.0 [53], 29.5 [53] | 1.1 [71, 130], 100 [71] (identified by NGO) | 27.5 [205], 32.9 [134], 70.4 [205] |  | 38.6 [138] | 14.2 [12], 16.3 [12], 18.5 [12], 25.0 [12] | 14.1 [142], 15.5 [142] |  | 7.0 [146] |  | 13.4 [31], 14.1 [203] | 6.0 [150] |

*Abbreviations*: *Afg* Afghanistan, *Alg* Algeria, *FSWs* female sex workers, *Leb* Lebanon, *Lib* Libya, *M* month(s), *Mor* Morocco, *Somal* Somalia

**References**

1. Moher D, Liberati A, Tetzlaff J, Altman DG, Group P: **Preferred reporting items for systematic reviews and meta-analyses: the PRISMA statement**. *PLoS medicine* 2009, **6**(7):e1000097.

2. Abu-Raddad L, Akala FA, Semini I, Riedner G, Wilson D, Tawil O: **Characterizing the HIV/AIDS epidemic in the Middle East and North Africa : Time for strategic action**. Washington DC: The World Bank Press; 2010.

3. Mumtaz G, Hilmi N, McFarland W, Kaplan RL, Akala FA, Semini I, Riedner G, Tawil O, Wilson D, Abu-Raddad LJ: **Are HIV epidemics among men who have sex with men emerging in the middle east and north Africa?: A systematic review and data synthesis**. *PLoS Medicine* 2011, **8 (8) (no pagination)**(e1000444).

4. Mumtaz GR, Weiss HA, Thomas SL, Riome S, Setayesh H, Riedner G, Semini I, Tawil O, Akala FA, Wilson D *et al*: **HIV among people who inject drugs in the Middle East and North Africa: systematic review and data synthesis**. *PLoS medicine* 2014, **11**(6):e1001663.

5. SAR AIDS Human Development Sector-The World Bank: **Mapping and situation assessment of key populations at high risk of HIV in three cities of Afghanistan**. In*.*, vol. 23; 2008.

6. National AIDS Control Program, Johns Hopkins University Bloomberg School of Public Health HIV Surveillance Project: **Integrated biological & behavioral surveillance (IBBS) in selected cities of Afghanistan: findings of 2012 IBBS survey and comparison to 2009 IBBS survey**. In*.* Kabul, Afghanistan; 2012.

7. Trellu-Kane M.: **Étude sur les Connaissances, Attitudes et Pratiques des jeunes djiboutiens** In*.*; 2005.

8. Jacobsen J.O. STJ, Loo V.,: **Estimating the size of key affected populations at elevated risk for HIV in Egypt**. In*.* Cairo, Egypt; 2014.

9. Karami M, Mirzaei M, Khazaei S, Bathaei SJ: **Estimating the population of female sex workers in Hamadan, Western iran, 2014**. *International Journal of High Risk Behaviors and Addiction* 2017, **6**(4):e63195.

10. Sharifi H, Karamouzian M, Baneshi MR, Shokoohi M, Haghdoost A, McFarland W, Mirzazadeh A: **Population size estimation of female sex workers in Iran: Synthesis of methods and results**. *PLoS One* 2017, **12**(8):e0182755.

11. Karami M, Khazaei S, Poorolajal J, Soltanian A, Sajadipoor M: **Estimating the population size of female sex worker population in Tehran, Iran: Application of direct capture-recapture method**. *AIDS and behavior* 2017, **21**:2394-2400.

12. Ministry of Health-Morocco, The Joint United Nations Programme on HIV/AIDS (UNAIDS), THe Global Fund: **HIV integrated behavioral and biological surveillance surveys-Morocco 2011: Female sex workers in Agadir, Fes, Rabat and Tanger**. In*.* Morocco; 2012.

13. Huygens P, Mellakh K: **Cartographie des professionnelles du sexe et des hommes ayant des relations sexuelles avec des hommes au Maroc: Agadir, Inezgane, Ait Melloul**. In*.*: Fonds Mondial; 2013.

14. National AIDS Control Program-Ministry of Health: **Integrated biological & behavioral surveillance: a pilot study in Karachi & Rawalpindi**. In*.* Pakistan; 2005.

15. National AIDS Control Program: **HIV second generation surveillance in Pakistan: National report round I**. In*.* Pakistan: Canada-Pakistan HIV/AIDS Surveillance Project; 2005.

16. Emmanuel F, Blanchard J, Zaheer HA, Reza T, Holte-McKenzie M: **The HIV/AIDS Surveillance Project mapping approach: an innovative approach for mapping and size estimation for groups at a higher risk of HIV in Pakistan**. *Aids* 2010, **24 Suppl 2**:S77-84.

17. Khan MS, Unemo M, Zaman S, Lundborg CS: **HIV, STI prevalence and risk behaviours among women selling sex in Lahore, Pakistan**. *BMC Infectious Diseases* 2011, **11 (no pagination)**(119).

18. National AIDS Control Programme Pakistan: **Mapping and behavioural study of adolescents in 7 districts of Pakistan: Karachi, Larkana, Quetta, Faisalabad, Lahore, Mardan, and Peshawar.** In*.* Islamabad, Pakistan; 2008.

19. Emmanuel F, Thompson LH, Athar U, Salim M, Sonia A, Akhtar N, Blanchard JF: **The organisation, operational dynamics and structure of female sex work in Pakistan**. *Sexually Transmitted Infections* 2013, **89**(SUPPL. 2):ii29-ii33.

20. National AIDS Control Program: **HIV second generation surveillance in Pakistan. National Report Round IV 2011**. In*.* Islamabad, Pakistan; 2012.

21. Punjab AIDS Control Program, AP Consultancies, Bridge Consultants Foundation: **Mapping of most at risk populations-Punjab 2014**. In*.* Lahore, Pakistan; 2015.

22. National AIDS Control Program: **Integrated biological & behavioral surveillance in Pakistan 2016-17: 2nd generation HIV surveillance in Pakistan round 5**. In*.* Islamabad, Pakistan; 2017: 159.

23. World Health Organization: **HIV Surveillance Systems: Regional update 2011**. In*.*; 2011.

24. Somalia Ministry of Health: **Mapping and size estimation of key populations in Somalia**. In*.* Somalia; 2016.

25. Sudan National AIDS Control Program: **Situation analysis: Behavioral & epidemiological surveys and response analysis**. In*.* Khartoum, Sudan; 2002.

26. Sudan National AIDS Control Program: **Mapping and behavioural survey of tea sellers and female sex workers in South Darfur State**. In*.* Khartoum, Sudan; 2005.

27. United Nations High Commissioner for Refugees (UNHCR): **HIV Behavioural Surveillance Survey Juba Municipality, South Sudan**. In*.*; 2007.

28. South Sudan HIV/AIDS Commission: **South Sudan Global AIDS Response Progress Report 2016**. In*.* South Sudan; 2015.

29. South Sudan HIV/AIDS Commission: **South Sudan Global AIDS Response Progress Report 2016**. In*.* South Sudan; 2016

30. Government of the Republic of South Sudan-Ministry of Health: **A Bio-Behavioral HIV Survey of Female Sex Workers in South Sudan**. In*.* South Sudan; 2016.

31. Hsairi M., Ben Abdallah S.: **Enquête sérocomportementale du VIH auprès des travailleuses du sexe clandestines en Tunisie**. In*.* Tunis, Tunisia; 2012.

32. Ministry of Health-Republic of Yemen: **Population size estimates among most at risk populations in five major cities in Yemen**. In*.* Yemen; 2010.

33. Mansoor AB, Fungladda W, Kaewkungwal J, Wongwit W: **Gender differences in kap related to HIV/AIDS among freshmen in Afghan Universities**. *Southeast Asian Journal of Tropical Medicine and Public Health* 2008, **39**(3):404-418.

34. Shokoohi M, Baneshi MR, Haghdoost AA: **Size estimation of groups at high risk of HIV/AIDS using network scale up in Kerman, Iran**. *International Journal of Preventive Medicine* 2012, **3**(7):471-476.

35. Khalajabadi Farahani F, Akhondi MM, Shirzad M, Azin A: **Hiv/Sti Risk-Taking Sexual Behaviours and Risk Perception among Male University Students in Tehran: Implications for Hiv Prevention among Youth**. *Journal of biosocial science* 2018, **50**(1):86-101.

36. Melikian L, Prothro ET: **Sexual behavior of university students in the Arab Near East**. *J Abnorm Psychol* 1954, **49**(1):59-64.

37. Melikian LH: **Social change and sexual behavior of Arab university students**. *The Journal of social psychology* 1967, **73**(2):169-175.

38. Ghandour LA, Mouhanna F, Yasmine R, El Kak F: **Factors associated with alcohol and/or drug use at sexual debut among sexually active university students: cross-sectional findings from Lebanon**. *BMC public health* 2014, **14**:671.

39. Faisel A., Cleland J.: **Study of the sexual behaviours and prevalence of STIs among migrant men in Lahore, Pakistan.** In*.*; 2005.

40. Minhas M.R., Haider K.: **Frequency of risk factors for acquiring HIV/AIDS among hostellers of professional institutes in Pakistan**. In: *International AIDS Society, MoPe1110C26: 2005*; 2005.

41. Ismail SO, Ahmed HJ, Grillner L, Hederstedt Issa BA, Bygdeman S: **Sexually transmitted diseases in men in Mogadishu, Somalia**. *International Journal of STD and AIDS* 1990, **1**(2):102-106.

42. Ismail SO, Ahmed HJ, Jama MA, Omer K, Omer FM, Brundin M, Olofsson MB, Grillner L, Bygdeman S: **Syphilis, gonorrhoea and genital chlamydial infection in a Somali village**. *Genitourin Med* 1990, **66**(2):70-75.

43. McCarthy MC, Hyams KC, El-Tigani El-Hag A, El-Dabi MA, El-Sadig El-Tayeb M, Khalid IO, George JF, Constantine NT, Woody JN: **HIV-1 and hepatitis B transmission in Sudan**. *Aids* 1989, **3**(11):725-729.

44. Holt BY, Brady W, Belay E, Toole M, Effler P, Friday J, Parker K: **Planning STI/HIV prevention among refugees and mobile populations: Situation assessment of Sudanese refugees**. *Disasters* 2003, **27**(1):1-15.

45. Ministry of Health-United Arab Emirates: **United Arab Emirates – Global AIDS Response Progress Report 2014**. In*.* United Arab Emirates; 2014.

46. **Additional country-level data provided through the MENA HIV/AIDS Epidemiology Synthesis Project database by the World Health Organization Regional Office for the Eastern Mediterranean**. 2013.

47. Jenkins C., Robalino D.A.: **HIV/AIDS in the Middle East and North Africa: The costs of inaction**. Washigton, D.C.: The World Bank; 2003.

48. Ministere de la Sante et de la Population et de la Reforme Hospitaliere, Programme National de Lutte Contre les MST et le SIDA: **Plan a Moyen Terme: 3 ans**. In*.* Geneva, Switzerland; 1990.

49. Addad B, S. Hamdi, A. Bouguermauh,: **Prevalence des MST en Consultation de Gynecologie - Obstetrique et dans le Milieu Carceral de la Prostitution**. In: *VIII International Conference on AIDS in Africa, Marrakech, Morocco, 12/12-16, Abstract WPC076: 1993*; 1993.

50. Ministere de la Sante et de la Population et de la Reforme Hospitaliere, Direction de la Prevention Comite National de Lutte contre les IST/VIH/SIDA: **Plan national strategique de lutte contre les IST/VIH/Sida 2008-2012**. In*.* Geneva, Switzerland; 2009.

51. The Joint United Nations Programme on HIV/AIDS (UNAIDS), World Health Organization, The United Nations Children's Fund (UNICEF): **UNAIDS/WHO/UNICEF Epidemiological Fact Sheets on HIV and AIDS, 2008 update**. In*.*

52. Ministere de la Sante et de la Population et de la Reforme Hospitaliere, Direction de la Prevention Comite National de Lutte contre les IST/VIH/SIDA: **Plan national strategique de lutte contre les IST/VIH/SIDA 2013-2015**. In*.* Geneva, Switzerland; 2016.

53. Ministere de la Sante et de la Population et de la Reforme Hospitaliere: **Rapport d'activite sur la riposte nationale au VIH/SIDA, Algerie 2014**. In*.* Algerie; 2014.

54. Ministere de la Sante et de la Population et de la Reforme Hospitaliere: **Rapport narratif de la riposte au VIH/SIDA**. In*.* Algerie; 2017.

55. Ministere de la Sante et de la Population et de la Reforme Hospitaliere: **Country progress report- Algeria: Global AIDS monitoring 2018**. In*.* Algeria; 2018.

56. Ministry of Health-Kingdom of Bahrain: **UNGASS Country Progress Report - Kingdom of Bahrain: January 2010 - December 2011**. In*.*; 2012.

57. Bailly C, M. Santiago, M. Abbate, et al.: **Situation in Djibouti: Sero Epidemiological Survey**. In: *III International Conference: AIDS and Associated Cancers in Africa, Sept 14-16, Poster.* 1988.

58. Organisation Mondiale pour la Sante-Djibouti: **Etudes epidemiologiques sur le VIH/SIDA et les IST a Djibouti de 1986 a 2001**. *Bulletin Epidémiologique Hebdomadaire de l’OMS* 2001, **49**.

59. Ministry of Health-Djibouti: **Rapport de la Surveillance de l'Infection a VIH par Pasles Sentinelles en Republique de Djibouti, juillet - octobre 1993**. In*.*; 1993.

60. Shrestha PN: **Forthcoming WER Global Update of AIDS Cases Reported to the World Health Organization (WHO)**. In*.*; 1999.

61. U.S. Department of State: **HIV/AIDS-- Recipe for Crisis: Djibouti's Mobile Population, Lack of Resources and Social Conservatism Equal World's 12th**. *Unclassified Cable, November, Djibouti, 001995* 2000.

62. Bahdon DG: **Situation de L'Infection a VIH/SIDA et des Maladies Sexuellement Transmissibles en Republique de Djibouti**. In*.*; 1998.

63. Ministere de la Sante Publique et des Affaires Sociales- Programme National de Lutte Contre le SIDA: **Rapport Epidemiologique de la Situation de L'Infection a VIH/SIDA du 4eme Trimestre 1998, Djibouti**. In*.*; 1999.

64. Ministry of Health- Djibouti, Programme de Lutte contre le SIDA: **Seroprevalence du VIH Chez les Femmes Travailleuses du Sexe a Djibouti**. In*.*; 2008.

65. Comite Technique Intersectoriel de Lutte contre le SIDA le PALUDISME et la Tuberculose (CTILSPT): **Rapport de suivi de la declaration d'engagement sur le VIH/SIDA-UNGASS 2010**. In*.* Djibouti; 2010.

66. Mourad A, S. Mostafa, D. Watts: **Low Prevalence of HIV Infection in Egyptian Nationals**. In: *VIII International Conference on AIDS, Amsterdam, 7/19-24, Abstract PuC 8147: 1992*; 1992.

67. Egypt Ministry of Health and Population, World Health Organization (WHO): **HIV/ AIDS Surveillance in Egypt: 2001**. In: *Eleventh Inter-Country Meeting of National AIDS Programme Managers, 7/23-26, Casablanca, Morocco: 2001*; 2001.

68. Murugasampillay S: **Epidemiology and Surveillance of Human Immunodeficiency Virus (HIV), Acquired Immunodeficiency Syndrome (AIDS) and Sexually Transmitted Infections**. In*.* Geneva, Switzerland; 1995: 1-45.

69. United States Census Bureau: **HIV/AIDS surveillance database**. In*.* Washington, DC; 2017.

70. Arafa M., Sallam S.: **Epidemiologic study of HIV/AIDs among high risk groups in Alexandria, Egypt**. In: *International AIDS Conference, CDB400: 2007*; 2007.

71. National AIDS Program-Egypt: **UNGASS country progress report**. In*.* Cairo, Egypt; 2014.

72. National AIDS Program-Egypt: **Country progress report-Egypt: Global AIDS monitoring 2017**. In*.* Egypt; 2017.

73. Iran National AIDS Control Programme: **Results of HIV Tests**. In*.* Iran; 1994.

74. Eltayeb EM: **HIV Surveillance in the Islamic Republic of Iran**. In*.* Geneva, Switzerland; 1995.

75. Feizzadeh A, Nedjat S, Asghari S, Keshtkar A, Heshmat R, Setayesh H, Majdzadeh R: **Evidence-based approach to HIV/AIDS policy and research prioritization in the Islamic Republic of Iran**. *Eastern Mediterranean health journal = La revue de sante de la Mediterranee orientale = al-Majallah al-sihhiyah li-sharq al-mutawassit* 2010, **16**(3):259-265.

76. Office of Deputy Minister of Health in Health Affairs-Center for Disease Management, The Joint United Nations Programme on HIV/AIDS (UNAIDS), The Iranian Center for AIDS Research: **Islamic Republic of Iran country report on UNGASS declaration of commitment**. In*.* Iran; 2006.

77. El-Tayeb E.M.: **Strengthening AIDS/HIV Surveillance in Jordan: 17-31 October 1994**. In*.*; 1995.

78. Lebanon National AIDS Programme, World Health Organization: **Republic of Lebanon National AIDS Programme - Strategic Plan: 1995 - 1999**. In*.* Beirut, Lebanon; 1994.

79. Riedner G: **HIV Surveillance among Key Populations at Risk in MENA Region: Recent Developments**. In: *2nd Global HIV/AIDS Surveillance Meeting, Bangkok, Thailand, 3/2-5, Tuesday Session VI, presentation no 1: 2009*; 2009.

80. National AIDS Control Program-Lebanon: **UNGASS country progress report 2010**. In*.* Lebanon; 2010.

81. Shazly M.: **Strengthening of HIV/AIDS Surveillance in the Libyan Arab Jamahiriya: 20 December 1990 - 19 January 1991**. In*.*; 1991.

82. Benslimane A, Rivjad M., Sekkat S. ea: **Incidence of HIV Infections in Morocco**. In: *II International Symposium: AIDS and Associated Cancers in Africa: 1987; Naples, Italy,10/7-9, Abstract TH-84.*; 1987.

83. Riyad M, Serrhini O, Sekkat S, al. e: **Transmission Sexuelle du HIV au Maroc**. In: *V International Conference: AIDS in Africa: 1990; Kinshasa, Zaire, Oct. 10-12, Poster T.P.C.5.*; 1990.

84. Royaume du Maroc Ministere de la Sante: **Situation Epidemiologique des IST et VIH-SIDA au Maroc**. In*.* Morocco; 2008.

85. Ministere de la Sante-Royaume du Maroc: **Historique de la surveillance sentinelle VIH 1999-2012 Maroc**. 2013.

86. Ministry of Health-Morocco: **Implementation of the Declaration of Commitment on HIV/AIDS: 2006 national report**. In*.* Morocco; 2006.

87. Bennani A., Alami K.: **Surveillance sentinelle du VIH: Resultats 2005 et tendances de la seroprevalence du VIH**. In*.*; 2006.

88. Royaume du Maroc Ministere de la Sante: **Mise en oeuvre de la declaration d'engagement sur le VIH/SIDA: Rapport national 2010**. 2010.

89. Ministere de la Sante-Royaume du Maroc: **Rapport sur les estimations de l'epidemie du VIH/sida au Maroc**. In*.*; 2013.

90. Loudyi B, A. B. Moussa, F. Barodi, et al.,: **Utilisation du Preservatif Chez les Professionnelles dd Sexe dans la Region de Fes**. In: *17th International Conference on AIDS and STIs in Africa, Cape Town, South Africa, 12/7-11, Poster, p 173: 2013*; 2013.

91. Girgis TH: **Strengthening of HIV/AIDS Surveillance Activities in Pakistan**. In*.*; 1990.

92. Rizvi AA, A. Ali Shah, S. Sheikh, et al.: **To Develop Changed Sex Behaviour in CSWs**. In: *5th International Congress on AIDS in Asia and the Pacific, Kuala Lumpur, Malaysia, 10/20-27, Abstract SCD02-02: 1999*; 1999.

93. Shah SA, A. K. Ghauri, M. A. Memon, et al.: **Voluntary HIV Counseling and Testing in a Peer Outreach Program for Female and Male Sex Workers in Sindh Province, Pakistan**. In: *6th International Congress on AIDS in Asia and the Pacific, Melbourne, Australia, 10/5-10, Abstract Mo0572: 2001*; 2001.

94. Pasha MSK, Qazi M. S.,: **Women's Health and HIV/AIDS: Researching with Home Based Sex Workers in Quetta, Pakistan**. In: *XVII International AIDS Conference, Mexico City, Mexico, 8/3-8, Abstract ThPe0326: 2008*; 2008.

95. Pasha MSK, Malik, M., Gull S.,: **HIV/STIs Epidemiology among Female Sex Workers: Faisalabad, Pakistan**. In: *9th International Congress on AIDS in Asia and the Pacific, Bali, Indonesia, 8/9-13, Abstract TuPA020: 2009*; 2009.

96. Pasha MSK, Baig M. A.,: **Anal Sex, among Female Sex Workers & Their Clients - Karachi, Pakistan**. In: *10th International Congress on AIDS in Asia and the Pacific, Busan, Korea, 8/26-30, Session SaOA04-05: 2011*; 2011.

97. Mir AS, Malick N. Z.,: **A Holistic Integrated Approach to HIV Prevention and Harm Reduction in among Female Sex Workers**. In: *11th International Congress on AIDS in Asia and the Pacific, Bangkok, Thailand, 11/18-22, Abstract 1977: 2013*; 2013.

98. Omar M. GA, Burans J., et al.,: **Ongoing Surveillance for HIV Amongst STD Patients in Somalia**. In: *IV International Conference on AIDS, Stockholm, 6/15-16, Poster 5557: 1988*; 1988.

99. Duffy G: **Report on STD/HIV Prevalence Study in Somaliland: Part 2**. In*.*; 1999.

100. Ahmed S.M., Kheir E.H.H.M. : **Sudanese Sexual Behaviour in the Context of Socio-Cultural Norms and the Transmission of HIV**. In: *Anthropological Studies Relevant to the Sexual Transmission of HIV. Volume 11*, edn. Sonderborg, Denmark; 1990: 19-22.

101. Basha H.M.: **Vulnerable Population Research in Darfur**. In*.*; 2006.

102. Elrashied S. M.: **HIV Sero-Prevalence and Related Risky Sexual Behaviours among Female Sex Workers (FSWs) in Khartoum State, Sudan**. In: *5th International AIDS Society Conference on HIV Pathogenesis, Treatment and Prevention, Cape Town, South Africa, 7/19-22, Online Session WEPEC103: 2009*; 2009.

103. El-Tayeb E. M.: **HIV/STD Surveillance in the Syrian Arab Republic: 20 May - 19 June 1995**. In*.* Damascus, Syria; 1995.

104. Ministry of Health-Syria: **National strategy for HIV/AIDS control**. In*.* Damascus, Syria; 2005.

105. Syria National AIDS Control Program: **HIV and AIDS situation epidemic in Syria**. In*.* Damascus, Syria; 2008.

106. Al Sayed S.: **Syrian Arab Republic UNGASS country progress report 2010**. In*.* Damascus, Syria; 2010.

107. Van de Perre P. CM: **HIV Infection in Prostitutes in Africa**. In: *AIDS in Children, Adolescents and Heterosexual Adults.* edn.: Elsevier Science Publishing Company, Inc.; 1988: 166-167.

108. Giraldo G. SD, Mugerwa R., et al.,: **Seroepidemiologic Analyses on Populations from Uganda and Tunisia-High and Low Risk African Regions for HIV Infections** In: *IV International Conference on AIDS, Stockholm, 6/13-14, Poster 5038: 1988*; 1988.

109. Gharbi Y. GM, Blibech R., et al.: **Epidemiology of HIV Infection in Tunisia**. In: *II International Symposium: AIDS and Associated Cancers in Africa, Naples, Italy, 10/7-9, Abstract TH-49: 1987*; 1987.

110. Taibi J: **Statistics on AIDS Cases Reported**. *Joint Publication Research Service: Epidemiology,* 1989, **10**:15.

111. Tunisia Ministere de la Sante Publique: **Plan a Moyen Terme 1990-1993**. In*.*; 1990.

112. Fekih Z. LF, Sidhom M.: **The Profile of HIV-Infected Tunisians Results of 5 Years Surveillance: 1986-1990**. In*.* Tunisia; 1991.

113. Programme de Lutte contre les IST/SIDA: **Analyse de la situation et de la reponse au VIH/SIDA en Tunisie**. In*.* Tunisia; 2005.

114. Ministere de la Sante Publique-Tunisie: **Appui au partenariat et renforcement de la riposte a la menace d'extension du VIH/SIDA en Tunisie**. In*.* Tunisie; 2006.

115. The Joint United Nations Programme on HIV/AIDS (UNAIDS): **Notes on AIDS in the Middle East and North Africa**. In*.* RST, MENA; 2008.

116. Ministere de la Sante Publique-Tunisie: **Rapport de situation national a l'intention de l'UNGASS 2010**. In*.* Tunisie; 2010.

117. Bahaa T., Elkamhawi S., Abdel Rahman I., Moustafa M., Shawky S., Kabore I., Soliman C.: **Gender influence on VCT seeking in Egypt**. In: *International AIDS Society, WEPE0255: 2010*; 2010.

118. Kahhaleh JG, El Nakib M, Jurjus AR: **Knowledge, attitudes, beliefs and practices in Lebanon concerning HIV/AIDS, 1996-2004**. *Eastern Mediterranean Health Journal* 2009, **15**(4):920-933.

119. Bennani A., El Rhilani H., El Kettani A., Latifi A., El Omari B., Alami K., Johnston L.G.: **Estimates of the size of key populations at risk for HIV infection: female sex workers and men who have sex with men, injecting drug users in Morocco in 2013**. In: *International AIDS COnference, WEPE180: 2014*; 2014.

120. Royaume du Maroc-Ministere de la Sante: **Enquete connaissances, attitudes et pratiques des jeunes en matiere d'IST et VIH/SIDA**. In*.*; 2013.

121. Projects and Research Department (AFROCENTER GROUP): **Baseline study on knowledge, attitudes, and practices on sexual behaviors and HIV/AIDS prevention amongst young people in selected states in Sudan**. In*.* Sudan; 2005.

122. Todd CS, Barbera-Lainez Y, Doocy SC, Ahmadzai A, Delawar FM, Burnham GM: **Prevalence of human immunodeficiency virus infection, risk behavior, and HIV knowledge among tuberculosis patients in Afghanistan**. *Sexually Transmitted Diseases* 2007, **34**(11):878-882.

123. Todd CS, Nasir A, Mansoor GF, Sahibzada SM, Jagodzinski LL, Salimi F, Khateri MN, Hale BR, Barthel RV, Scott PT: **Cross-sectional assessment of prevalence and correlates of blood-borne and sexually-transmitted infections among Afghan National Army recruits**. *BMC Infectious Diseases* 2012, **12 (no pagination)**(196).

124. Adib SM, Akoum S, El-Assaad S, Jurjus A: **Heterosexual awareness and practices among Lebanese male conscripts**. *Eastern Mediterranean Health Journal* 2002, **8**(6):765-775.

125. Royaume du Maroc-Ministere de la Sante, Cooperation Technique Allemande/GTZ: **Enquete connaissances, attitudes et pratiques des jeunes concernant les IST et le SIDA**. In*.*; 2007.

126. Mir AM, Wajid A, Pearson S, Khan M, Masood I: **Exploring urban male non-marital sexual behaviours in Pakistan**. *Reproductive Health* 2013, **10 (1) (no pagination)**(22).

127. Sudan National AIDS Control Programme-Federal Ministry of Health: **HIV/AIDS/STIs knowledge attitude behavioural and practice among university students and military personnel, Sudan 2004**. In*.* Khartoum, Sudan; 2004.

128. National AIDS Control Program, Johns Hopkins University Bloomberg School of Public Health HIV Surveillance Project: **Integrated behavioral & biological surveillance (IBBS) in Afghanistan: Year 1 report**. In*.* Kabul, Afghanistan; 2010.

129. Ministry of Health, National AIDS Program, Family Health International: **HIV/AIDS biological & behavioral surveillance survey round I: Summary report**. In*.* Cairo, Egypt; 2006.

130. Ministry of Health, National AIDS Program, Family Health International, Center for Development Services: **HIV/AIDS biological & behavioral surveillance survey round II: Summary report**. In*.* Egypt; 2010.

131. Navadeh S, Mirzazadeh A, Mousavi L, Haghdoost A, Fahimfar N, Sedaghat A: **HIV, HSV2 and Syphilis Prevalence in Female Sex Workers in Kerman, South-East Iran; Using Respondent-Driven Sampling**. *Iran J Public Health* 2012, **41**(12):60-65.

132. Sajadi L, Mirzazadeh A, Navadeh S, Osooli M, Khajehkazemi R, Gouya MM, Fahimfar N, Zamani O, Haghdoost AA: **HIV prevalence and related risk behaviours among female sex workers in Iran: results of the national biobehavioural survey, 2010**. *Sexually transmitted infections* 2013, **89 Suppl 3**:iii37-40.

133. Kazerooni PA, Motazedian N, Motamedifar M, Sayadi M, Sabet M, Lari MA, Kamali K: **The prevalence of human immunodeficiency virus and sexually transmitted infections among female sex workers in Shiraz, South of Iran: By respondent-driven sampling**. *International Journal of STD and AIDS* 2014, **25**(2):155-161.

134. Moayedi-Nia S, Bayat Jozani Z, Esmaeeli Djavid G, Entekhabi F, Bayanolhagh S, Saatian M, Sedaghat A, Nikzad R, Jahanjoo Aminabad F, Mohraz M: **HIV, HCV, HBV, HSV, and syphilis prevalence among female sex workers in Tehran, Iran, by using respondent-driven sampling**. *AIDS Care* 2016, **28**(4):487-490.

135. Mirzazadeh A, M. Shokoohi, R. Khajehkazemi, et al.: **HIV and sexually transmitted infections among female sex workers in Iran: Findings from the 2010 and 2015 national surveillance surveys**. In: *21st International AIDS Conference, Durban, South Africa, 7/18-22, ePoster, Abstract TUPEC175: 2016*; 2016.

136. Ministry of Health-Hashemite Kingdom of Jordan: **Report to the Secretary General of the United Nations on the United Nations General Assembly Special Session on HIV/AIDS.** In*.*; 2014.

137. Mahfoud Z, Afifi R, Ramia S, Khoury DE, Kassak K, Barbir FE, Ghanem M, El-Nakib M, Dejong J: **HIV/AIDS among female sex workers, injecting drug users and men who have sex with men in Lebanon: Results of the first biobehavioral surveys**. *Aids* 2010, **24**(SUPPL. 2):S45-S54.

138. Valadez JJ, Berendes S, Jeffery C, Thomson J, Ben Othman H, Danon L, Turki AA, Saffialden R, Mirzoyan L: **Filling the Knowledge Gap: Measuring HIV Prevalence and Risk Factors among Men Who Have Sex with Men and Female Sex Workers in Tripoli, Libya**. *PLoS ONE* 2013, **8 (6) (no pagination)**(e66701).

139. Bokhari A, Nizamani NM, Jackson DJ, Rehan NE, Rahman M, Muzaffar R, Mansoor S, Raza H, Qayum K, Girault P *et al*: **HIV risk in Karachi and Lahore, Pakistan: an emerging epidemic in injecting and commercial sex networks**. *International journal of STD & AIDS* 2007, **18**(7):486-492.

140. National AIDS Control Program-Ministry of Health: **HIV second generation surveillance in Pakistan: national report round II**. In*.* Pakistan; 2007.

141. Hawkes S, Collumbien M, Platt L, Lalji N, Rizvi N, Andreasen A, Chow J, Muzaffar R, Ur-Rehman H, Siddiqui N *et al*: **HIV and other sexually transmitted infections among men, transgenders and women selling sex in two cities in Pakistan: A cross-sectional prevalence survey**. *Sexually Transmitted Infections* 2009, **85**(SUPPL. 2):ii8-ii16.

142. National AIDS Control Program-Pakistan Ministry of Health: **Progress report on the Declaration of Commitment on HIV/AIDS for the United Nations General Assembly Special Session on HIV/AIDS**. In*.* Islamabad, Pakistan; 2010.

143. Testa A, Kriitmaa K: **HIV & Syphilis bio-behavioural surveillance survey (BSS) among female transactional sex workers in Hargeisa, Somaliland**. In*.* Somalia; 2008.

144. International Organization for Migration (IOM): **Integrated biological and behavioural surveillance survey among vulnerable women in Hargeisa, Somaliland**. In*.* Geneva, Switzerland; 2017.

145. Elkarim M.A.A. AHA, Ahmed S.M., et al.,: **Situation Analysis: Behavioral & Epidemiological Surveys & Response Analysis - HIV/AIDS Strategic Planning Process**. In*.*; 2002.

146. Abdelrahim MS: **HIV prevalence and risk behaviors of female sex workers in Khartoum, north Sudan**. *Aids* 2010, **24**(SUPPL. 2):S55-S60.

147. Sudan National AIDS Control Programme: **UNGASS report 2008-2009, North Sudan.** In*.*; 2010.

148. Sudan National AIDS Control Program: **Integrated bio-behavioral HIV surveillance (IBBS) among female sex workers and men who have sex with men in 15 states of Sudan, 2011-2012**. In*.*; 2012.

149. Stulhofer A, Bozicevic I: **HIV bio-behavioural survey among FSWs in Aden, Yemen**. In*.*; 2008.

150. Ministry of Health-Republic of Yemen: **UNGASS Country Progress Report 2013**. In*.* Yemen.; 2014.

151. Todd CS, Nasir A, Stanekzai MR, Bautista CT, Botros BA, Scott PT, Strathdee SA, Tjaden J: **HIV, hepatitis B, and hepatitis C prevalence and associated risk behaviors among female sex workers in three Afghan cities**. *Aids* 2010, **24 Suppl 2**:S69-75.

152. Rodier GR, Couzineau B, Gray GC, Omar CS, Fox E, Bouloumie J, Watts D: **Trends of human immunodeficiency virus type-1 infection in female prostitutes and males diagnosed with a sexually transmitted disease in Djibouti, East Africa**. *American Journal of Tropical Medicine and Hygiene* 1993, **48**(5):682-686.

153. Constantine NT, Fox E, Rodier G, Abbatte EA: **Monitoring for HIV-1, HIV-2, HTLV-I sero-progression and sero-conversion in a population at risk in east Africa**. *The Journal of the Egyptian Public Health Association* 1992, **67**(5-6):535-547.

154. Couzineau B, Bouloumie J, Hovette P, Laroche R: **Prevalence of AIDS infection in target people of the Republic of Djibouti. [French]**. *Medecine Tropicale* 1991, **51**(4):485-486.

155. Philippon M, Saada M, Kamil MA, Houmed HM: **Attendance at a health center of clandestine prostitutes in Djibouti. [French]**. *Cahiers Sante* 1997, **7**(1):5-10.

156. Marcelin AG, Grandadam M, Flandre P, Nicand E, Milliancourt C, Koeck JL, Philippon M, Teyssou R, Agut H, Dupin N *et al*: **Kaposi's sarcoma herpesvirus and HIV-1 seroprevalences in prostitutes in Djibouti**. *Journal of Medical Virology* 2002, **68**(2):164-167.

157. Sheba MF, Woody JN, Zaki AM, Morrill JC, Burans J, Farag I, Kashaba S, Madkour S, Mansour M: **The prevalence of HIV infection in Egypt**. *Transactions of the Royal Society of Tropical Medicine and Hygiene* 1988, **82**(4):634.

158. Watts DM, Constantine NT, Sheba MF, Kamal M, Callahan JD, Kilpatrick ME: **Prevalence of HIV infection and AIDS in Egypt over four years of surveillance (1986-1990)**. *The Journal of tropical medicine and hygiene* 1993, **96**(2):113-117.

159. Kabbash IA, Abdul-Rahman I, Shehata YA, Omar AA: **HIV infection and related risk behaviours among female sex workers in greater Cairo, Egypt**. *Eastern Mediterranean health journal = La revue de sante de la Mediterranee orientale = al-Majallah al-sihhiyah li-sharq al-mutawassit* 2012, **18**(9):920-927.

160. Jahani MR, Alavian SM, Shirzad H, Kabir A, Hajarizadeh B: **Distribution and risk factors of hepatitis B, hepatitis C, and HIV infection in a female population with "illegal social behaviour"**. *Sexually transmitted infections* 2005, **81**(2):185.

161. Kassaian N, Ataei B, Yaran M, Babak A, Shoaei P, Ataie M: **HIV and other sexually transmitted infections in women with illegal social behavior in Isfahan, Iran**. *Adv Biomed Res* 2012, **1**:5.

162. Taghizadeh H, Taghizadeh F, Fathi M, Reihani P, Shirdel N, Rezaee SM: **Drug use and high-risk sexual behaviors of women at a drop-in center in mazandaran province, Iran, 2014**. *Iranian Journal of Psychiatry and Behavioral Sciences* 2015, **9**(2):49-55.

163. Asadi-Ali Abadi M, Abolghasemi J, Rimaz S, Majdzadeh R, Shokoohi M, Rostami-Maskopaee F, Merghati-Khoei E: **High-Risk Behaviors Among Regular and Casual Female Sex Workers in Iran: A Report from Western Asia**. *Iran J Psychiatry Behav Sci* 2018, **In Press**(In Press):e9744.

164. Naman RE, Mokhbat JE, Farah AE, Zahar KL, Ghorra FS: **Seroepidemiology of the human immunodeficiency virus in Lebanon. Preliminary evaluation**. *Le Journal medical libanais* 1989, **The Lebanese medical journal. 38**(1):5-8.

165. Royaume du Maroc-Ministere de la Sante: **Etude de prevalence des IST ches les femmes qui consultent pour pertes vaginales et/ou douleurs du bas ventre**. In: *Programme National de lutte contre les IST/SIDA.* Rabat, Maroc; 2008.

166. Iqbal J, Rehan N: **Sero-prevalence of HIV: six years' experience at Shaikh Zayed Hospital, Lahore**. *Jpma* 1996, **The Journal of the Pakistan Medical Association. 46**(11):255-258.

167. Baqi S, Nabi N, Hasan SN, Khan AJ, Pasha O, Kayani N, Haque RA, Haq IU, Khurshid M, Fisher-Hoch S *et al*: **HIV antibody seroprevalence and associated risk factors in sex workers, drug users, and prisoners in Sindh, Pakistan**. *Journal of acquired immune deficiency syndromes and human retrovirology : official publication of the International Retrovirology Association* 1998, **18**(1):73-79.

168. Anwar M, Jaffery G, Rasheed S: **Serological Screening of Female Prostitutes for Anti-HIV and Hepatitis B Surface Antigen**. *Pak J Health* 1998, **35**(3-4):69-73.

169. Shah A.S., Memon M.A., Soomro S., Kazi N., Kristensen S.: **Seroprevelance of HIV, Syphilis, Hepatitis B and Hepatitis C among female commercial sex workers in Hyderabad, Pakistan**. In: *International AIDS Conference, C12368: 2004*; 2004.

170. Shah A.S., Ghauri A.K., Memon M.A., Shaikh S.A., Abbas S.Q., Kristensen S.: **HIV infection trends in the Sindh Province of Pakistan**. In: *International AIDS Conference, C12336: 2004*; 2004.

171. Akhtar A., Aslam M., Arif M., Rehman K.: **Safer sex knowledge and attitude of female sex workers in Pakistan**. In: *International AIDS Conference, THPE0334: 2008*; 2008.

172. Raza M, Ikram N, Saeed N, Waheed U, Kamran M, Iqbal R, Bakar M: **HIV/AIDS and Syphilis Screening Among High Risk Groups**. *J Rawal Med Coll* 2015, **19**(1):11-14.

173. Jama H, Grillner L, Biberfeld G, Osman S, Isse A, Abdirahman M, Bygdeman S: **Sexually transmitted viral infections in various population groups in Mogadishu, Somalia**. *Genitourinary Medicine* 1987, **63**(5):329-332.

174. Burans JP, Fox E, Omar MA, Farah AH, Abbass S, Yusef S, Guled A, Mansour M, Abu-Elyazeed R, Woody JN: **HIV infection surveillance in Mogadishu, Somalia**. *East African medical journal* 1990, **67**(7):466-472.

175. Scott DA, Corwin AL, Constantine NT, Omar MA, Guled A, Yusef M, Roberts CR, Watts DM: **Low prevalence of human immunodeficiency virus-1 (HIV-1), HIV-2, and human T cell lymphotropic virus-1 infection in Somalia**. *American Journal of Tropical Medicine and Hygiene* 1991, **45**(6):653-659.

176. Corwin AL, Olson JG, Omar MA, Razaki A, Watts DM: **HIV-1 in Somalia: Prevalence and knowledge among prostitutes** *Aids* 1991, **5**(7):902-904.

177. Jama Ahmed H, Omar K, Adan SY, Guled AM, Grillner L, Bygdeman S: **Syphilis and human immunodeficiency virus seroconversion during a 6-month follow-up of female prostitutes in Mogadishu, Somalia**. *International Journal of STD and AIDS* 1991, **2**(2):119-123.

178. Burans JP, McCarthy M, el Tayeb SM, el Tigani A, George J, Abu-Elyazeed R, Woody JN: **Serosurvey of prevalence of human immunodeficiency virus amongst high risk groups in Port Sudan, Sudan**. *East African medical journal* 1990, **67**(9):650-655.

179. McCarthy MC, Khalid IO, El Tigani A: **HIV-1 infection in Juba, southern Sudan**. *Journal of Medical Virology* 1995, **46**(1):18-20.

180. Bchir A, Jemni L, Saadi M, Milovanovic A, Brahim H, Catalan F: **Markers of sexually transmitted diseases in prostitutes in central Tunisia**. *Genitourinary medicine* 1988, **64**(6):396-397.

181. Hassen E, Chaieb A, Letaief M, Khairi H, Zakhama A, Remadi S, Chouchane L: **Cervical human papillomavirus infection in Tunisian women**. *Infection* 2003, **31**(3):143-148.

182. Znazen A, Frikha-Gargouri O, Berrajah L, Bellalouna S, Hakim H, Gueddana N, Hammami A: **Sexually transmitted infections among female sex workers in Tunisia: High prevalence of Chlamydia trachomatis**. *Sexually Transmitted Infections* 2010, **86**(7):500-505.

183. Fox E, Haberberger Jr RL, Abbatte EA, Said S, Polycarpe D, Constantine NT: **Observations on sexually transmitted diseases in promiscuous males in Djibouti**. *The Journal of the Egyptian Public Health Association* 1989, **64**(5-6):561-569.

184. Al-Owaish RA, Anwar S, Sharma P, Shah SF: **HIV/AIDS prevalence among male patients in Kuwait**. *Saudi medical journal* 2000, **21**(9):852-859.

185. Alowaish R, Anwar S.: **Sexually transmitted diseases among bachelor community in Kuwait**. In: *International AIDS Conference, C11000: 2002*; 2002.

186. Al-Mutairi N, Joshi A, Nour-Eldin O, Sharma AK, El-Adawy I, Rijhwani M: **Clinical patterns of sexually transmitted diseases, associated sociodemographic characteristics, and sexual practices in the Farwaniya region of Kuwait**. *International Journal of Dermatology* 2007, **46**(6):594-599.

187. Heikel J, Sekkat S, Bouqdir F, Rich H, Takourt B, Radouani F, Hda N, Ibrahimy S, Benslimane A: **The prevalence of sexually transmitted pathogens in patients presenting to a Casablanca STD clinic**. *European journal of epidemiology* 1999, **15**(8):711-715.

188. Manhart LE, A. Zidouh, K. Holmes, et al.: **Sexually Transmitted Disease (STD) in Three Types of Health Clinics in Morocco: Prevalence, Risk Factors, and Syndromic Management**. In: *XI International Conference on AIDS, Vancouver, 7/7-14, Poster MoC1627: 1996*; 1996.

189. Alami K, Mbarek Ait N, Akrim M, Bellaji B, Hansali A, Khattabi H, Sekkat A, El Aouad R, Mahjour J: **Urethral discharge in Morroco: Prevalence of microorganisms and susceptibility of gonococcos**. *Eastern Mediterranean Health Journal* 2002, **8**(6):794-804.

190. Mujeeb SA, Hafeez A: **Prevalence and pattern of HIV infection in Karachi**. *Jpma* 1993, **The Journal of the Pakistan Medical Association. 43**(1):2-4.

191. Memon GM: **Serosurveillance of HIV infection in people at risk in Hyderabad Sindh**. *JPMA The Journal of the Pakistan Medical Association* 1997, **47**(12):302-304.

192. National AIDS Programme: **HIV seroprevalence surveys in Pakistan**. *AIDS* 1996, **10**(8):926-927.

193. Rehan N: **Profile of men suffering from sexually transmitted infections in Pakistan**. *Journal of Ayub Medical College, Abbottabad : JAMC* 2003, **15**(2):15-19.

194. Bhutto AM, Shah AH, Ahuja DK, Solangi AH, Shah SA: **Pattern of sexually transmitted infections in males in interior Sindh: a 10-year-study**. *Journal of Ayub Medical College, Abbottabad : JAMC* 2011, **23**(3):110-114.

195. Razvi SK, Najeeb S, Nazar HS: **Pattern of sexually transmitted diseases in patients presenting at Ayub teaching hospital, Abbottabad**. *Journal of Ayub Medical College, Abbottabad : JAMC* 2014, **26**(4):582-583.

196. National AIDS Control program, Balochistan AIDS Control program, Canada Pakistan HIV/AIDS Surveillance Project: **Bio behavioral survey among mine workers in Balochistan, Pakistan**. In*.* Islamabad, Pakistan; 2012.

197. Ismail A., Ekanem E., Deq S., Arube P., Gboun M.: **Somaliland 2007 HIV/Syphilis sero-prevalence survey: A technical report**. In*.*; 2007.

198. McCarthy MC, Burans JP, Constantine NT, El-Tigani El-Hag AA, El-Saddig El-Tayeb M, El-Dabi MA, Fahkry JG, Woody JN, Hyams KC: **Hepatitis B and HIV in Sudan: A serosurvey for hepatitis B and human immunodeficiency virus antibodies among sexually active heterosexuals**. *American Journal of Tropical Medicine and Hygiene* 1989, **41**(6):726-731.

199. Hashemite Kingdom of Jordan: **Report to the Secretary General of the United Nations on the United Nations General Assembly Special Session on HIV/AIDS**. In*.*; 2010.

200. Ministry of Health-Pakistan National AIDS Control Program: **National study of reproductive tract and sexually transmitted infections: a survey of high risk groups in Lahore and Karachi, Pakistan**. In*.*; 2005.

201. Punjab AIDS Control Program, AP Consultancies, Bridge Consultants Foundation: **Integrated behavioural & biological surveillance among most at-risk population, IBBS study-Punjab 2014**. In*.* Lahore, Pakistan; 2015.

202. Elhadi M, Elbadawi A, Abdelrahman S, Mohammed I, Bozicevic I, Hassan EA, Elmukhtar M, Ahmed S, Abdelraheem MS, Mubarak N *et al*: **Integrated bio-behavioural HIV surveillance surveys among female sex workers in Sudan, 2011-2012**. *Sexually transmitted infections* 2013, **89 Suppl 3**:iii17-22.

203. Ministere de la Sante Publique-Tunisie: **Synthese des enquetes de seroprevalence et serocomportementales aupres de trois populations vulnerables au VIH: Les usages de drogues injectables, les hommes ayant des rapports sexuels avec des hommes, et les travailleuses dy sexe clandestines en Tunisie**. In*.* Tunisia; 2010.

204. Syria National AIDS Control Program: **HIV/AIDS female sex wrokers KABP survey in Syria**. In*.* Damascus, Syria; 2004.

205. Shokoohi M, Noori A, Karamouzian M, Sharifi H, Khajehkazemi R, Fahimfar N, Hosseini-Hooshyar S, Kazerooni PA, Mirzazadeh A: **Remaining Gap in HIV Testing Uptake Among Female Sex Workers in Iran**. *AIDS and behavior* 2017, **21**(8):2401-2411.

206. Mirzazadeh A, Nedjat S, Navadeh S, Haghdoost A, Mansournia MA, McFarland W, Mohammad K: **HIV and related risk behaviors among female sex workers in Iran: bias-adjusted estimates from the 2010 National Bio-Behavoral Survey**. *AIDS and behavior* 2014, **18 Suppl 1**:S19-24.
